# Supplementary material for: Codon optimization underpins generalist parasitism in fungi
Source: eLife. 2017 Feb 3;6:e22472. doi: 10.7554/eLife.22472 (PMC5315462; doi:10.7554/eLife.22472)
Supplement: Figure 1—source data 1. — DOI: http://dx.doi.org/10.7554/eLife.22472.003 [file elife-22472-fig1-data1.docx]

**Equations forming the mathematical model of protein biosynthesis related to protein length and codon optimization parameters.**

| No. | Output parameter | Equation |
| --- | --- | --- |
| (1) | Codon decoding time | $CDT=-60\cdot tAI+90$ |
| (2) | Codon decoding rate | $CDR=\frac{1000}{CDT}$ |
| (3) | Protein synthesis time | $PST=CDT\cdot L$ |
| (4) | Protein synthesis rate | $PSR=\frac{CDR}{L}$ |
| (5) | Variation of the free ribosome pool | $\frac{{dP}_{r}}{dt}=+P_{rb, r}\cdot\frac{{CDR}_{r}}{L_{r}}-(\sum P_{r}\cdot{mRNA}_{i}\cdot Ki)-P_{r}\cdot\mu$  See parameters in **Table below** |
| (6) | variation of the intracellular pool of secreted proteins P(*s*) | $\frac{{dP}_{s}}{dt}=+P_{rb, s}\cdot\frac{{CDR}_{s}}{L_{s}}-P_{s}\cdotѴ-P_{r}\cdot\mu$  With Ѵ the rate of protein secretion, µ the dilution rate due to cell volume growth |
| (7) | variation of the extracellular pool of secreted proteins | $\frac{{dP}_{e}}{dt}=+P_{s}\cdotѴ-P_{e}\cdot\mu$ |
| (8) | The variation of the intracellular pool of intracellular proteins | $\frac{{dP}_{n}}{dt}=+P_{rb, n}\cdot\frac{{CDR}_{n}}{L_{n}}-P_{n}\cdot\mu$ |
| (9) | variation of the ribosomes bound to mRNA(*i*) | $\frac{{dP}_{rb,i}}{dt}=+P_{r}\cdot{mRNA}_{i}\cdot Ki-P_{rb, i}\cdot\frac{{CDR}_{i}}{L_{i}}-P_{rb,i}\cdot\mu$ |
| (10) | variation of the free mRNA(*i*) concentration | $\frac{{dmRNA}_{i}}{dt}=-P_{r}\cdot{mRNA}_{i}\cdot Ki+P_{rb, i}\cdot\frac{{CDR}_{i}}{L_{i}}$ |
| (11) | maximal number of proteins that a finite cell can contain | $\frac{d{PSR}_{i}}{dt}=\frac{{CDR}_{i}}{L_{i}}\cdot(1+\frac{1}{\left( \sum P_{i}\cdot\beta-P_{max}\cdot\beta\right)})$ |

**List of parameters and variables used for modeling of growth rate based on proteome properties**

| ***paramaters*** | ***Class*** | ***symbol*** | ***value*** |
| --- | --- | --- | --- |
| codon decoding rate, ribosomal proteins | parameter | CDR(r ) | median value of the organism |
| codon decoding rate, intracellular proteins | parameter | CDR(n) | median value of the organism |
| codon decoding rate, secreted proteins | parameter | CDR(s) | median value of the organism |
| Length ribosomal proteins | parameter | L(r ) | median value of the organism |
| Length intracellular proteins | parameter | L(n) | median value of the organism |
| Length secreted proteins | parameter | L(s) | median value of the organism |
| association constant of ribosomal mRNA | parameter | K(r ) | 10000 |
| association constant of intracellular proteins mRNA | parameter | K(n ) | 10000 |
| association constant of secreted proteins mRNA | parameter | K(s ) | 10000 |
| growth rate | parameter | µ | fitted parameter |
| secretion rate of secreted proteins | parameter | $Ѵ$ | 0.05 |
| maximal proteins content of the cell | parameter | P(max) | 0.0083 |
| strength of crowding effect | parameter | β | 12048 |
| ***variables*** |  |  | **Intital value** |
| free ribosomal proteins pool | variable | P(r ) | 0.001 |
| not secreted proteins pool | variable | P(n) | 0.0001 |
| intracellular to be secreted proteins pool | variable | P(s) | 0.0001 |
| extracellular secreted proteins pool | variable | P(e ) | 0 |
| ribome bound to mRNAr | variable | P(rb, r) | 0 |
| ribome bound to mRNAn | variable | P(rb, n) | 0 |
| ribome bound to mRNAs | variable | P(rb, s) | 0 |
| messenger ARN of ribosomal proteins | variable | mRNA(r ) | 1.66e-05 |
| messenger ARN of not secreted proteins | variable | mRNA(n) | 5.81e-05 |
| messenger ARN of secreted proteins | variable | mRNA(s) | 8.3e-06 |

**Values of parameters used for modeling of growth rate based on proteome properties**

| Species | Median size of non secreted proteins | Median size of secreted proteins | Median size of Ribosomal proteins |
| --- | --- | --- | --- |
| *Verticilium dahliae* | 413 | 382.5 | 248 |
| *Sclerotinia sclerotiorum* | 392 | 389 | 190 |
| *Rhizoctonia solani* | 409 | 374 | 192 |
| *Rhizopus oryzae* | 255 | 280 | 150 |
| *Pyrenophora tritici-repentis* | 375 | 324 | 189 |
| *Penicillium digitatum* | 375 | 384 | 216 |
| *Metarhizium acridum* | 410 | 374 | 213 |
| *Fusarium graminearum* | 367 | 366.5 | 173 |
| *Cryptococcus neoformans* | 458 | 422 | 227.5 |
| *Colletotrichum graminicola* | 395 | 354 | 157 |
| *Botrytis cinerea* | 425.5 | 394 | 190 |
| *Beauveria bassiana* | 425 | 375 | 202 |
| *Batrachochytrium dendrobatidis* | 369 | 294.5 | 176.5 |
| *Aspergillus fumigatus* | 417 | 395 | 212 |
| *Alternaria brassicicola* | 376 | 364 | 196 |
| **GENERALISTS** | **381** | **365** | **189** |
|  |  |  |  |
| *Wolfiporia cocos* | 346 | 399 | 166 |
| *Rozella allomycis* | 295 | 364.5 | 197 |
| *Puccinia triticina* | 347 | 266 | 193 |
| *Puccinia graminis* | 270 | 235 | 195.5 |
| *Ophiocordyceps unilateralis* | 431 | 389.5 | 217 |
| *Nosema ceranae* | 195 | 255.5 | 155 |
| *Zymoseptoria tritici* | 405 | 370.5 | 256 |
| *Pseudocercospora fijiensis* | 366 | 372 | 175 |
| *Moniliophthora roreri* | 348 | 354 | 173.5 |
| *Erysiphe necator* | 386 | 351.5 | 194 |
| *Dothistroma septosporum* | 337 | 381 | 189 |
| *Passalora fulva* | 361 | 350 | 191 |
| *Blumeria graminis* | 418 | 227.5 | 170 |
| **SPECIALISTS** | **346** | **318** | **192** |
