# Supplementary material for: Codon optimization underpins generalist parasitism in fungi
Source: eLife. 2017 Feb 3;6:e22472. doi: 10.7554/eLife.22472 (PMC5315462; doi:10.7554/eLife.22472)
Supplement: Figure 2—source data 1. — Results of an analysis of variance (ANOVA) considering the number of contigs in genome assemblies (no. contigs, as a descriptor of the quality of assemblies), host range and their interaction as factors; values of host range, number of contigs and codon optimization (S) for each genome. DOI: http://dx.doi.org/10.7554/eLife.22472.005 [file elife-22472-fig2-data1.docx]

**Figure 2 – source data 1.** Codon optimization is dependent on breadth of host range but not genome assembly parameters. Results of an analysis of variance (ANOVA) considering the number of contigs in genome assemblies (no. contigs, as a descriptor of the quality of assemblies), host range and their interaction as factors.

| All genomes (45) |  |  |  |  |  |  |
| --- | --- | --- | --- | --- | --- | --- |
|  | Df | Sum Sq | Mean Sq | F value | Pr(>F) | signif. |
| no. contigs | 1 | 0.1693 | 0.1693 | 4.762 | 0.0344 |  |
| **host range** | 1 | 0.7559 | 0.7559 | 21.26 | **3.32E-05** | ******* |
| no.contigs:host range | 1 | 0.0348 | 0.0348 | 0.978 | 0.3281 |  |
| Residuals | 45 | 1.6 | 0.0356 |  |  |  |
| --- |  |  |  |  |  |  |
| Signif. Codes: | 1.00E-04 | *** | 1.00E-03 | ** | 0.01 | * |
|  |  |  |  |  |  |  |
| Top 36 assemblies (<1000 contigs) | |  |  |  |  |  |
|  | Df | Sum Sq | Mean Sq | F value | Pr(>F) | signif. |
| no. contigs | 1 | 0.0066 | 0.0066 | 0.174 | 0.679 |  |
| **host range** | 1 | 0.7248 | 0.7248 | 19.01 | **9.97E-05** | ******* |
| no.contigs:host range | 1 | 0.0664 | 0.0664 | 1.743 | 0.195 |  |
| Residuals | 37 | 1.4107 | 0.0381 |  |  |  |
| --- |  |  |  |  |  |  |
| Signif. Codes: | 1.00E-04 | *** | 1.00E-03 | ** | 0.01 | * |

Values of host range, number of contigs and codon optimization (S) for each genome

|  | **Host range** | **no contigs** | **S** |
| --- | --- | --- | --- |
| ***Tuber melanosporum*** | **0** | **7** | **0.230** |
| ***Myceliophthora thermophila*** | **0** | **7** | **0.246** |
| ***Magnaporthe oryzae*** | **4** | **8** | **0.437** |
| ***Aspergillus fumigatus*** | **300** | **8** | **0.609** |
| ***Encephalitozoon intestinalis*** | **7** | **11** | **0.313** |
| ***Cryptococcus neoformans*** | **800** | **14** | **0.843** |
| ***sclerotinia sclerotiorum*** | **332** | **16** | **0.524** |
| ***Dothistroma septosporum*** | **1** | **20** | **0.211** |
| ***Zymoseptoria tritici*** | **1** | **21** | **-0.019** |
| ***Agaricus bisporus*** | **0** | **29** | **0.361** |
| ***Fusarium graminearum*** | **72** | **31** | **0.788** |
| ***Serpula lacrymans*** | **0** | **36** | **-0.001** |
| ***Chaetomium globosum*** | **0** | **37** | **0.148** |
| ***Sporisorium reilianum*** | **5** | **44** | **0.476** |
| ***Pyrenophora tritici-repentis*** | **11** | **48** | **0.419** |
| ***Laccaria bicolor*** | **0** | **55** | **-0.026** |
| ***Verticilium dahliae*** | **78** | **55** | **0.537** |
| ***Pseudocercospora fijiensis*** | **1** | **56** | **0.227** |
| ***Batrachochytrium dendrobatidis*** | **500** | **69** | **0.545** |
| ***Rhizopus oryzae*** | **28** | **81** | **0.625** |
| ***Botrytis cinerea*** | **556** | **82** | **0.597** |
| ***Rhodotorula toruloides*** | **0** | **94** | **0.331** |
| ***Oidiodendron maius*** | **0** | **100** | **0.147** |
| ***Penicillium digitatum*** | **17** | **100** | **0.545** |
| ***Stagonospora nodorum*** | **7** | **108** | **0.155** |
| ***Beauveria bassiana*** | **700** | **235** | **0.616** |
| ***Metarhizium acridum*** | **228** | **241** | **0.647** |
| ***Rhizoctonia solani*** | **690** | **326** | **0.432** |
| ***Wolfiporia cocos*** | **2** | **348** | **0.217** |
| ***Gonapodya prolifera*** | **0** | **352** | **0.115** |
| ***Puccinia graminis*** | **2** | **394** | **-0.032** |
| ***Taphrina deformans*** | **4** | **394** | **0.403** |
| ***Melampsora larici-populina*** | **7** | **462** | **0.194** |
| ***Nosema ceranae*** | **1** | **536** | **0.021** |
| ***Colletotrichum graminicola*** | **59** | **654** | **0.572** |
| ***Alternaria brassicicola*** | **16** | **838** | **0.400** |
| *Rozella allomycis* | 0 | 1059 | 0.236 |
| *Pseudogymnoascus destructans (Geomyces destructans)* | 8 | 1846 | 0.484 |
| *Moniliophthora roreri* | 2 | 3280 | 0.406 |
| *Passalora fulva* | 2 | 4865 | -0.011 |
| *Erysiphe necator* | 1 | 5935 | 0.174 |
| *Ophiocordyceps unilateralis* | 1 | 6790 | 0.166 |
| *Blumeria graminis* | 1 | 6843 | 0.116 |
| *Colletotrichum higginsianum* | 6 | 10235 | 0.393 |
| *Puccinia triticina* | 1 | 14820 | 0.204 |
