# Supplementary material for: Codon optimization underpins generalist parasitism in fungi
Source: eLife. 2017 Feb 3;6:e22472. doi: 10.7554/eLife.22472 (PMC5315462; doi:10.7554/eLife.22472)
Supplement: Figure 3—source data 1. — DOI: http://dx.doi.org/10.7554/eLife.22472.009 [file elife-22472-fig3-data1.docx]

**Figure 3 – source data 1.** Overview of host range features for the 45 fungal species analyzed in this work

| **Species** | **Code name** | **NCBI ID** | **Source for host range information** | **host type** | **Number of host genera** |
| --- | --- | --- | --- | --- | --- |
| *Rozella allomycis* | Rozal | 281847 | http://genome.jgi.doe.gov/Rozal1_1/Rozal1_1.home.html | Fungi | 1 |
| *Rhizopus oryzae* | Rhior | 64495 | http://nt.ars-grin.gov/fungaldatabases/fungushost/FungusHost.cfm Plants | | 28 |
| *Nosema ceranae* | Nocse_v2 | 40302 | Maside X, Gómez-Moracho T, Jara L, Martín-Hernández R, De la Rúa P, Higes M, Bartolomé C. Population Genetics of Nosema apis and Nosema ceranae: One Host (Apis mellifera) and Two Different Histories. PLoS One. 2015 Dec 31;10(12):e0145609. | Insects | 1 |
| *Encephalitozoon intestinalis* | Encin | 58839 | Bornay-Llinares FJ, da Silva AJ, Moura H, Schwartz DA, Visvesvara GS, Pieniazek NJ, Cruz-López A, Hernández-Jaúregui P, Guerrero J, Enriquez FJ. Immunologic, microscopic, and molecular evidence of Encephalitozoon intestinalis (Septata intestinalis) infection in mammals other than humans. J Infect Dis. 1998 Sep;178(3):820-6.; Mathis A, Weber R, Deplazes P. Zoonotic potential of the microsporidia. Clin Microbiol Rev. 2005 Jul;18(3):423-45. | Mammals | 7 |
| *Batrachochytrium dendrobatidis* | Batde | 109871 | Olson DH, Aanensen DM, Ronnenberg KL, Powell CI, Walker SF, Bielby J, Garner TW, Weaver G; Bd Mapping Group, Fisher MC. Mapping the global emergence of Batrachochytrium dendrobatidis, the amphibian chytrid fungus. PLoS One. 2013;8(2):e56802. | Animals | 153 |
| *Gonapodya prolifera* | Gonpr | 1E+06 | non pathogenic | None | 0 |
| *Sporisorium reilianum* | Spore | 72558 | http://nt.ars-grin.gov/fungaldatabases/fungushost/FungusHost.cfm | Plants | 5 |
| *Rhodotorula toruloides* | Rhoto | 5286 | non pathogenic | None | 0 |
| *Melampsora larici-populina* | Mellp | 203908 | http://nt.ars-grin.gov/fungaldatabases/fungushost/FungusHost.cfm | Plants | 7 |
| *Puccinia triticina* | Puctr | 208348 | http://nt.ars-grin.gov/fungaldatabases/fungushost/FungusHost.cfm | Plants | 1 |
| *Puccinia graminis* | Pucgr | 5297 | http://nt.ars-grin.gov/fungaldatabases/fungushost/FungusHost.cfm | Plants | 2 |
| *Cryptococcus neoformans* | Cryne | 5207 | The hosts include birds, mammals, amoeba, insects (diptera and lepidoptera), and nematodes based on Lin X, Heitman J. The biology of the Cryptococcus neoformans species complex. Annu Rev Microbiol. 2006;60:69-105. The corresponding number of genera was 2154 for birds (https://en.wikipedia.org/wiki/List_of_bird_genera); 1258 for mammals (https://en.wikipedia.org/wiki/List_of_mammal_genera), 1346 for Diptera (http://bugguide.net/node/view/52/tree/all), 2268 for Lepidoptera (http://bugguide.net/node/view/52/tree/all), 30 for amoeaba (https://en.wikipedia.org/wiki/Amoebozoa), 456 for nematodes (http://plpnemweb.ucdavis.edu/nemaplex/Uppermnus/Classifmnu.htm), giving a total of 7512. We considered that host range covers 8% of these genera (601) as a conservative estimate. | Animals, Insects,… | 800 |
| *Wolfiporia cocos* | Wolco | 81056 | http://nt.ars-grin.gov/fungaldatabases/fungushost/FungusHost.cfm Plants | | 2 |
| *Rhizoctonia solani* | Rhiso_v2 | 456999 | http://nt.ars-grin.gov/fungaldatabases/fungushost/FungusHost.cfm Plants | | 690 |
| *Serpula lacrymans* | Serla | 85982 | non pathogenic | None | 0 |
| *Moniliophthora roreri* | Monro | 221103 | http://nt.ars-grin.gov/fungaldatabases/fungushost/FungusHost.cfm | Plants | 2 |
| *Laccaria bicolor* | Lacbi | 29883 | non pathogenic | None | 0 |
| *Agaricus bisporus* | Agabi | 5341 | non pathogenic | None | 0 |
| *Taphrina deformans* | Tapde | 5011 | http://nt.ars-grin.gov/fungaldatabases/fungushost/FungusHost.cfm | Plants | 4 |
| *Tuber melanosporum* | Tubme | 39416 | non pathogenic | None | 0 |
| *Penicillium digitatum* | Pendi | 36651 | http://nt.ars-grin.gov/fungaldatabases/fungushost/FungusHost.cfm Plants | | 17 |
| *Aspergillus fumigatus* | Aspfu | 746128 | The host includes "Humans, cows, dolphins, birds, and horses" as per http://www.phac-aspc.gc.ca/lab-bio/res/psds-ftss/aspergillus-spp-eng.php:. The corresponding number of genera was 2154 for birds (https://en.wikipedia.org/wiki/List_of_bird_genera); 22 for dolphins (https://en.wikipedia.org/wiki/Dolphin); 10 for cows (https://en.wikipedia.org/wiki/Bovinae) and 1 for horses and human, giving a total of 2188 genera. We considered that host range covers 8% of these genera (175) as a conservative estimate. | Animals | 175 |
| *Stagonospora nodorum* | Stano | 13684 | http://nt.ars-grin.gov/fungaldatabases/fungushost/FungusHost.cfm | Plants | 7 |
| *Alternaria brassicicola* | Altbr | 29001 | http://nt.ars-grin.gov/fungaldatabases/fungushost/FungusHost.cfm | Plants | 16 |
| *Pyrenophora tritici-repentis* | Pyrtr | 45151 | http://nt.ars-grin.gov/fungaldatabases/fungushost/FungusHost.cfm | Plants | 11 |
| *Dothistroma septosporum* | Dotse | 64363 | http://nt.ars-grin.gov/fungaldatabases/fungushost/FungusHost.cfm | Plants | 1 |
| *Pseudocercospora fijiensis (Mycosphaerella fijiensis)* | Mycfi | 83344 | http://nt.ars-grin.gov/fungaldatabases/fungushost/FungusHost.cfm | Plants | 1 |
| *Zymoseptoria tritici (Mycosphaerella graminicola)* | Mycgr | 1E+06 | http://nt.ars-grin.gov/fungaldatabases/fungushost/FungusHost.cfm | Plants | 1 |
| *Passalora fulva* | Clafu | 5499 | http://nt.ars-grin.gov/fungaldatabases/fungushost/FungusHost.cfm | Plants | 2 |
| *Blumeria graminis* | Blugr | 34373 | http://nt.ars-grin.gov/fungaldatabases/fungushost/FungusHost.cfm | Plants | 1 |
| *Erysiphe necator* | Eryne | 52586 | http://nt.ars-grin.gov/fungaldatabases/fungushost/FungusHost.cfm | Plants | 1 |
| *Botrytis cinerea* | Botci_v2 | 40559 | http://nt.ars-grin.gov/fungaldatabases/fungushost/FungusHost.cfm | Plants | 556 |
| *Sclerotinia sclerotiorum* | Sclsc_v2 | 5180 | http://nt.ars-grin.gov/fungaldatabases/fungushost/FungusHost.cfm | Plants | 332 |
| *Oidiodendron maius* | Oidma | 78148 | non pathogenic | None | 0 |
| *Pseudogymnoascus destructans (Geomyces destructans)* | Geode | 655981 | https://en.wikipedia.org/wiki/Pseudogymnoascus_destructans | Animals | 8 |
| *Magnaporthe oryzae* | Maggr | 318829 | http://nt.ars-grin.gov/fungaldatabases/fungushost/FungusHost.cfm | Plants | 4 |
| *Myceliophthora thermophila* | Mycth | 78579 | non pathogenic | None | 0 |
| *Chaetomium globosum* | Chagl | 38033 | non pathogenic | None | 0 |
| *Verticilium dahliae* | Verda | 27337 | http://nt.ars-grin.gov/fungaldatabases/fungushost/FungusHost.cfm | Plants | 78 |
| *Colletotrichum higginsianum* | Colhi | 80884 | http://nt.ars-grin.gov/fungaldatabases/fungushost/FungusHost.cfm | Plants | 6 |
| *Colletotrichum graminicola* | Colgr | 31870 | http://nt.ars-grin.gov/fungaldatabases/fungushost/FungusHost.cfm | Plants | 59 |
| *Ophiocordyceps unilateralis* | Ophun | 268505 | de Bekker C, Quevillon LE, Smith PB, Fleming KR, Ghosh D, Patterson AD, Hughes DP. Species-specific ant brain manipulation by a specialized fungal parasite. BMC Evol Biol. 2014 Aug 29;14:166. | insects | 1 |
| *Beauveria bassiana* | Beaba | 176275 | The list of host families was obtained from Marcos R. de Faria, Stephen P. Wraight, Mycoinsecticides and Mycoacaricides: A comprehensive list with worldwide coverage and international classification of formulation types, Biological Control 2007 Dec;43(3):237-56.; the list of genus in these families (a total of 3366) was obtained from http://bugguide.net/node/view/52/tree/all. We considered that host range covers 8% of these genera (269) so that the number of infected genera does not exceeds that of infected species (700) reported in Xiao G, Ying SH, Zheng P, Wang ZL, Zhang S, Xie XQ, Shang Y, St Leger RJ, Zhao GP, Wang C, Feng MG Genomic perspectives on the evolution of fungal entomopathogenicity in Beauveria bassiana. Sci Rep. 2012;2:483. | Insects | 269 |
| *Fusarium graminearum* | Fusgr | 5518 | http://nt.ars-grin.gov/fungaldatabases/fungushost/FungusHost.cfm | Plants | 72 |
| *Metarhizium acridum* | Metac | 92637 | Insects from order orthoptera as per Hu X, Xiao G, Zheng P, Shang Y, Su Y, Zhang X, Liu X, Zhan S, St Leger RJ, Wang C. Trajectory and genomic determinants of fungal-pathogen speciation and host adaptation. Proc Natl Acad Sci U S A. 2014 Nov 25;111(47):16796-801. The list of genus from this order was obtained from http://bugguide.net/node/view/52/tree/all. | Insects | 228 |

Overview of genome features for the 45 fungal species analyzed in this work

| **Species** | **Genome sequence reference** | **Sequence files source** | **No. contigs** | **CDS** | **Full CDS** |
| --- | --- | --- | --- | --- | --- |
| *Rozella allomycis* | James TY, Pelin A, Bonen L, Ahrendt S, Sain D, Corradi N, Stajich JE Shared signatures of parasitism and phylogenomics unite Cryptomycota and microsporidia. Curr Biol. 2013 Aug 19;23(16):1548-53. | http://genome.jgi.doe.gov/Rozal1_1/Rozal1_1.download.html | 1059 | 6350 | 5932 |
| *Rhizopus oryzae* | Ma LJ, Ibrahim AS, Skory C, Grabherr MG, Burger G, Butler M, Elias M, Idnurm A, Lang BF, Sone T, Abe A, Calvo SE, Corrochano LM, Engels R, Fu J, Hansberg W, Kim JM, Kodira CD, Koehrsen MJ, Liu B, Miranda-Saavedra D, O'Leary S, Ortiz-Castellanos L, Poulter R, Rodriguez-Romero J, Ruiz-Herrera J, Shen YQ, Zeng Q, Galagan J, Birren BW, Cuomo CA, Wickes BL Genomic analysis of the basal lineage fungus Rhizopus oryzae reveals a whole-genome duplication. PLoS Genet. 2009 Jul;5(7):e1000549. | http://genome.jgi.doe.gov/Rhior3/Rhior3.home.html | 81 | 17467 | 17412 |
| *Nosema ceranae* | Cornman RS, Chen YP, Schatz MC, Street C, Zhao Y, Desany B, Egholm M, Hutchison S, Pettis JS, Lipkin WI, Evans JD Genomic analyses of the microsporidian Nosema ceranae, an emergent pathogen of honey bees. PLoS Pathog. 2009 Jun;5(6):e1000466. | http://www.ebi.ac.uk/ena/data/view/GCA_000988165.1 | 536 | 3208 | 2932 |
| *Encephalitozoon intestinalis* | Corradi N, Pombert JF, Farinelli L, Didier ES, Keeling PJ The complete sequence of the smallest known nuclear genome from the microsporidian Encephalitozoon intestinalis. Nat Commun. 2010 Sep 21;1:77. | http://genome.jgi.doe.gov/pages/dynamicOrganismDownload.jsf?organism=Encin1 | 11 | 1833 | 1830 |
| *Batrachochytrium dendrobatidis* | Batrachochytrium dendrobatidis Sequencing Project, Broad Institute of Harvard and MIT (http://www.broadinstitute.org/) | https://www.broadinstitute.org/annotation/genome/batrachochytrium_dendrobatidis/MultiDownloads.html | 69 | 8819 | 8805 |
| *Gonapodya prolifera* | Chang Y, Wang S, Sekimoto S, Aerts AL, Choi C, Clum A, LaButti KM, Lindquist EA, Yee Ngan C, Ohm RA, Salamov AA, Grigoriev IV, Spatafora JW, Berbee ML Phylogenomic Analyses Indicate that Early Fungi Evolved Digesting Cell Walls of Algal Ancestors of Land Plants. Genome Biol Evol. 2015 May 14;7(6):1590-601. | http://genome.jgi.doe.gov/Ganpr1/Ganpr1.download.html | 352 | 13902 | 13140 |
| *Sporisorium reilianum* | Schirawski J, Mannhaupt G, Münch K, Brefort T, Schipper K, Doehlemann G, Di Stasio M, Rössel N, Mendoza-Mendoza A, Pester D, Müller O, Winterberg B, Meyer E, Ghareeb H, Wollenberg T, Münsterkötter M, Wong P, Walter M, Stukenbrock E, Güldener U, Kahmann R. Pathogenicity determinants in smut fungi revealed by genome comparison. Science. 2010 Dec 10;330(6010):1546-8. | http://genome.jgi.doe.gov/pages/dynamicOrganismDownload.jsf?organism=Spore1 | 44 | 6648 | 6614 |
| *Rhodotorula toruloides* | Zhu Z, Zhang S, Liu H, Shen H, Lin X, Yang F, Zhou YJ, Jin G, Ye M, Zou H, Zhao ZK A multi-omic map of the lipid-producing yeast Rhodosporidium toruloides. Nat Commun. 2012;3:1112. | http://genome.jgi.doe.gov/Rhoto1/Rhoto1.home.html | 94 | 8140 | 8117 |
| *Melampsora larici-populina* | Duplessis S, Cuomo CA, Lin YC, Aerts A, Tisserant E, Veneault-Fourrey C, Joly DL, Hacquard S, Amselem J, Cantarel BL, Chiu R, Coutinho PM, Feau N, Field M, Frey P, Gelhaye E, Goldberg J, Grabherr MG, Kodira CD, Kohler A, Kües U, Lindquist EA, Lucas SM, Mago R, Mauceli E, Morin E, Murat C, Pangilinan JL, Park R, Pearson M, Quesneville H, Rouhier N, Sakthikumar S, Salamov AA, Schmutz J, Selles B, Shapiro H, Tanguay P, Tuskan GA, Henrissat B, Van de Peer Y, Rouzé P, Ellis JG, Dodds PN, Schein JE, Zhong S, Hamelin RC, Grigoriev IV, Szabo LJ, Martin F. Obligate biotrophy features unraveled by the genomic analysis of rust fungi. Proc Natl Acad Sci U S A. 2011 May 31;108(22):9166-71. | http://genome.jgi.doe.gov/pages/dynamicOrganismDownload.jsf?organism=Mellp2_3 | 462 | 16399 | 15525 |
| *Puccinia triticina* | “Puccinia Group Sequencing Project, Broad Institute of Harvard and MIT (http://www.broadinstitute.org/)” | https://www.broadinstitute.org/annotation/genome/puccinia_group/MultiDownloads.html | 14820 | 15585 | 13576 |
| *Puccinia graminis* | Duplessis S, Cuomo CA, Lin YC, Aerts A, Tisserant E, Veneault-Fourrey C, Joly DL, Hacquard S, Amselem J, Cantarel BL, Chiu R, Coutinho PM, Feau N, Field M, Frey P, Gelhaye E, Goldberg J, Grabherr MG, Kodira CD, Kohler A, Kües U, Lindquist EA, Lucas SM, Mago R, Mauceli E, Morin E, Murat C, Pangilinan JL, Park R, Pearson M, Quesneville H, Rouhier N, Sakthikumar S, Salamov AA, Schmutz J, Selles B, Shapiro H, Tanguay P, Tuskan GA, Henrissat B, Van de Peer Y, Rouzé P, Ellis JG, Dodds PN, Schein JE, Zhong S, Hamelin RC, Grigoriev IV, Szabo LJ, Martin F. Obligate biotrophy features unraveled by the genomic analysis of rust fungi. Proc Natl Acad Sci U S A. 2011 May 31;108(22):9166-71. | http://genome.jgi.doe.gov/pages/dynamicOrganismDownload.jsf?organism=Pucgr2 | 394 | 22534 | 15820 - 20534 ATG STOP unknown |
| *Cryptococcus neoformans* | Loftus BJ, Fung E, Roncaglia P, Rowley D, Amedeo P, Bruno D, Vamathevan J, Miranda M, Anderson IJ, Fraser JA, Allen JE, Bosdet IE, Brent MR, Chiu R, Doering TL, Donlin MJ, D'Souza CA, Fox DS, Grinberg V, Fu J, Fukushima M, Haas BJ, Huang JC, Janbon G, Jones SJ, Koo HL, Krzywinski MI, Kwon-Chung JK, Lengeler KB, Maiti R, Marra MA, Marra RE, Mathewson CA, Mitchell TG, Pertea M, Riggs FR, Salzberg SL, Schein JE, Shvartsbeyn A, Shin H, Shumway M, Specht CA, Suh BB, Tenney A, Utterback TR, Wickes BL, Wortman JR, Wye NH, Kronstad JW, Lodge JK, Heitman J, Davis RW, Fraser CM, Hyman RW The genome of the basidiomycetous yeast and human pathogen Cryptococcus neoformans. Science. 2005 Feb 25;307(5713):1321-4. | http://genome.jgi.doe.gov/Cryne_JEC21_1/Cryne_JEC21_1.download.html | 14 | 6475 | 6475 |
| *Wolfiporia cocos* | Floudas D, Binder M, Riley R, Barry K, Blanchette RA, Henrissat B, Martinez AT, Otillar R, Spatafora JW, Yadav JS, Aerts A, Benoit I, Boyd A, Carlson A, Copeland A, Coutinho PM, de Vries RP, Ferreira P, Findley K, Foster B, Gaskell J, Glotzer D, Gorecki P, Heitman J, Hesse C, Hori C, Igarashi K, Jurgens JA, Kallen N, Kersten P, Kohler A, Kues U, Kumar TK, Kuo A, LaButti K, Larrondo LF, Lindquist E, Ling A, Lombard V, Lucas S, Lundell T, Martin R, McLaughlin DJ, Morgenstern I, Morin E, Murat C, Nagy LG, Nolan M, Ohm RA, Patyshakuliyeva A, Rokas A, Ruiz-Duenas FJ, Sabat G, Salamov A, Samejima M, Schmutz J, Slot JC, St John F, Stenlid J, Sun H, Sun S, Syed K, Tsang A, Wiebenga A, Young D, Pisabarro A, Eastwood DC, Martin F, Cullen D, Grigoriev IV, Hibbett DS. The Paleozoic origin of enzymatic lignin decomposition reconstructed from 31 fungal genomes. Science. 2012 Jun 29;336(6089):1715-9. | http://genome.jgi-psf.org/Wolco1/Wolco1.home.html | 348 | 12746 | 11568 |
| *Rhizoctonia solani* | Cubeta MA, Thomas E, Dean RA, Jabaji S, Neate SM, Tavantzis S, Toda T, Vilgalys R, Bharathan N, Fedorova-Abrams N, Pakala SB, Pakala SM, Zafar N, Joardar V, Losada L, Nierman WC. Draft Genome Sequence of the Plant-Pathogenic Soil Fungus Rhizoctonia solani Anastomosis Group 3 Strain Rhs1AP. Genome Announc. 2014 Oct 30;2(5). pii: e01072-14. | http://www.ebi.ac.uk/ena/data/view/GCA_000524645.1 | 326 | 12737 | 9470 |
| *Serpula lacrymans* | Eastwood DC, Floudas D, Binder M, Majcherczyk A, Schneider P, Aerts A, Asiegbu FO, Baker SE, Barry K, Bendiksby M, Blumentritt M, Coutinho PM, Cullen D, de Vries RP, Gathman A, Goodell B, Henrissat B, Ihrmark K, Kauserud H, Kohler A, LaButti K, Lapidus A, Lavin JL, Lee YH, Lindquist E, Lilly W, Lucas S, Morin E, Murat C, Oguiza JA, Park J, Pisabarro AG, Riley R, Rosling A, Salamov A, Schmidt O, Schmutz J, Skrede I, Stenlid J, Wiebenga A, Xie X, Kues U, Hibbett DS, Hoffmeister D, Hogberg N, Martin F, Grigoriev IV, Watkinson SC. The plant cell wall-decomposing machinery underlies the functional diversity of forest fungi. Science. 2011 Aug 5;333(6043):762-5. | http://genome.jgi-psf.org/SerlaS7_9_2/SerlaS7_9_2.home.html | 36 | 12789 | 10740 - 11618 ATG no STOP |
| *Moniliophthora roreri* | Meinhardt LW, Costa GG, Thomazella DP, Teixeira PJ, Carazzolle MF, Schuster SC, Carlson JE, Guiltinan MJ, Mieczkowski P, Farmer A, Ramaraj T, Crozier J, Davis RE, Shao J, Melnick RL, Pereira GA, Bailey BA. Genome and secretome analysis of the hemibiotrophic fungal pathogen, Moniliophthora roreri, which causes frosty pod rot disease of cacao: mechanisms of the biotrophic and necrotrophic phases. BMC Genomics. 2014 Feb 27;15:164. | http://0-www.ncbi.nlm.nih.gov.elis.tmu.edu.tw/Traces/wgs/?val=AWSO01 | 3280 | 17910 | 16792 |
| *Laccaria bicolor* | Martin F, Aerts A, Ahrén D, Brun A, Danchin EG, Duchaussoy F, Gibon J, Kohler A, Lindquist E, Pereda V, Salamov A, Shapiro HJ, Wuyts J, Blaudez D, Buée M, Brokstein P, Canbäck B, Cohen D, Courty PE, Coutinho PM, Delaruelle C, Detter JC, Deveau A, DiFazio S, Duplessis S, Fraissinet-Tachet L, Lucic E, Frey-Klett P, Fourrey C, Feussner I, Gay G, Grimwood J, Hoegger PJ, Jain P, Kilaru S, Labbé J, Lin YC, Legué V, Le Tacon F, Marmeisse R, Melayah D, Montanini B, Muratet M, Nehls U, Niculita-Hirzel H, Oudot-Le Secq MP, Peter M, Quesneville H, Rajashekar B, Reich M, Rouhier N, Schmutz J, Yin T, Chalot M, Henrissat B, Kües U, Lucas S, Van de Peer Y, Podila GK, Polle A, Pukkila PJ, Richardson PM, Rouzé P, Sanders IR, Stajich JE, Tunlid A, Tuskan G, Grigoriev IV. The genome of Laccaria bicolor provides insights into mycorrhizal symbiosis. Nature. 2008 Mar 6;452(7183):88-92. | http://genome.jgi.doe.gov/pages/dynamicOrganismDownload.jsf?organism=Lacbi2 | 55 | 23130 | 20235 |
| *Agaricus bisporus* | Morin E, Kohler A, Baker AR, Foulongne-Oriol M, Lombard V, Nagy LG, Ohm RA, Patyshakuliyeva A, Brun A, Aerts AL, Bailey AM, Billette C, Coutinho PM, Deakin G, Doddapaneni H, Floudas D, Grimwood J, Hilden K, Kues U, Labutti KM, Lapidus A, Lindquist EA, Lucas SM, Murat C, Riley RW, Salamov AA, Schmutz J, Subramanian V, Wosten HA, Xu J, Eastwood DC, Foster GD, Sonnenberg AS, Cullen D, de Vries RP, Lundell T, Hibbett DS, Henrissat B, Burton KS, Kerrigan RW, Challen MP, Grigoriev IV, Martin F Genome sequence of the button mushroom Agaricus bisporus reveals mechanisms governing adaptation to a humic-rich ecological niche. Proc Natl Acad Sci U S A. 2012 Oct 23;109(43):17501-6. | http://genome.jgi.doe.gov/Agabi_varbisH97_2/Agabi_varbisH97_2.download.html | 29 | 10438 | 9411 |
| *Taphrina deformans* | Cissé OH, Almeida JM, Fonseca A, Kumar AA, Salojärvi J, Overmyer K, Hauser PM, Pagni M. Genome sequencing of the plant pathogen Taphrina deformans, the causal agent of peach leaf curl. MBio. 2013 Apr 30;4(3):e00055-13. | http://genome.jgi.doe.gov/pages/dynamicOrganismDownload.jsf?organism=Tapde1_1 | 394 | 4609 | 4528 |
| *Tuber melanosporum* | Martin F, Aerts A, Ahrén D, Brun A, Danchin EG, Duchaussoy F, Gibon J, Kohler A, Lindquist E, Pereda V, Salamov A, Shapiro HJ, Wuyts J, Blaudez D, Buée M, Brokstein P, Canbäck B, Cohen D, Courty PE, Coutinho PM, Delaruelle C, Detter JC, Deveau A, DiFazio S, Duplessis S, Fraissinet-Tachet L, Lucic E, Frey-Klett P, Fourrey C, Feussner I, Gay G, Grimwood J, Hoegger PJ, Jain P, Kilaru S, Labbé J, Lin YC, Legué V, Le Tacon F, Marmeisse R, Melayah D, Montanini B, Muratet M, Nehls U, Niculita-Hirzel H, Oudot-Le Secq MP, Peter M, Quesneville H, Rajashekar B, Reich M, Rouhier N, Schmutz J, Yin T, Chalot M, Henrissat B, Kües U, Lucas S, Van de Peer Y, Podila GK, Polle A, Pukkila PJ, Richardson PM, Rouzé P, Sanders IR, Stajich JE, Tunlid A, Tuskan G, Grigoriev IV. The genome of Laccaria bicolor provides insights into mycorrhizal symbiosis. Nature. 2008 Mar 6;452(7183):88-92. | http://genome.jgi.doe.gov/pages/dynamicOrganismDownload.jsf?organism=Tubme1 | 7 | 7496 | 7468 |
| *Penicillium digitatum* | Marcet-Houben M, Ballester AR, de la Fuente B, Harries E, Marcos JF, González-Candelas L, Gabaldón T. Genome sequence of the necrotrophic fungus Penicillium digitatum, the main postharvest pathogen of citrus. BMC Genomics. 2012 Nov 21;13:646. | http://genome.jgi.doe.gov/pages/dynamicOrganismDownload.jsf?organism=Pendi1 | 100 | 9118 | 9118 |
| *Aspergillus fumigatus* | Nierman WC, Pain A, Anderson MJ, Wortman JR, Kim HS, Arroyo J, Berriman M, Abe K, Archer DB, Bermejo C, Bennett J, Bowyer P, Chen D, Collins M, Coulsen R, Davies R, Dyer PS, Farman M, Fedorova N, Fedorova N, Feldblyum TV, Fischer R, Fosker N, Fraser A, Garcia JL, Garcia MJ, Goble A, Goldman GH, Gomi K, Griffith-Jones S, Gwilliam R, Haas B, Haas H, Harris D, Horiuchi H, Huang J, Humphray S, Jimenez J, Keller N, Khouri H, Kitamoto K, Kobayashi T, Konzack S, Kulkarni R, Kumagai T, Lafon A, Latge JP, Li W, Lord A, Lu C, Majoros WH, May GS, Miller BL, Mohamoud Y, Molina M, Monod M, Mouyna I, Mulligan S, Murphy L, O'Neil S, Paulsen I, Penalva MA, Pertea M, Price C, Pritchard BL, Quail MA, Rabbinowitsch E, Rawlins N, Rajandream MA, Reichard U, Renauld H, Robson GD, Rodriguez de Cordoba S, Rodriguez-Pena JM, Ronning CM, Rutter S, Salzberg SL, Sanchez M, Sanchez-Ferrero JC, Saunders D, Seeger K, Squares R, Squares S, Takeuchi M, Tekaia F, Turner G, Vazquez de Aldana CR, Weidman J, White O, Woodward J, Yu JH, Fraser C, Galagan JE, Asai K, Machida M, Hall N, Barrell B, Denning DW Genomic sequence of the pathogenic and allergenic filamentous fungus Aspergillus fumigatus. Nature. 2005 Dec 22;438(7071):1151-6. | http://genome.jgi.doe.gov/Aspfu1/Aspfu1.home.html | 8 | 9781 | 9749 |
| *Stagonospora nodorum* | Hane JK, Lowe RG, Solomon PS, Tan KC, Schoch CL, Spatafora JW, Crous PW, Kodira C, Birren BW, Galagan JE, Torriani SF, McDonald BA, Oliver RP. Dothideomycete plant interactions illuminated by genome sequencing and EST analysis of the wheat pathogen Stagonospora nodorum. Plant Cell. 2007 Nov;19(11):3347-68. | http://genome.jgi.doe.gov/pages/dynamicOrganismDownload.jsf?organism=Stano2 | 108 | 12380 | 12371 |
| *Alternaria brassicicola* | Ohm RA, Feau N, Henrissat B, Schoch CL, Horwitz BA, Barry KW, Condon BJ, Copeland AC, Dhillon B, Glaser F, Hesse CN, Kosti I, LaButti K, Lindquist EA, Lucas S, Salamov AA, Bradshaw RE, Ciuffetti L, Hamelin RC, Kema GH, Lawrence C, Scott JA, Spatafora JW, Turgeon BG, de Wit PJ, Zhong S, Goodwin SB, Grigoriev IV. Diverse lifestyles and strategies of plant pathogenesis encoded in the genomes of eighteen Dothideomycetes fungi. PLoS Pathog. 2012;8(12):e1003037. | http://genome.jgi.doe.gov/Altbr1/Altbr1.download.html | 838 | 10688 | 9115 |
| *Pyrenophora tritici-repentis* | Manning VA, Pandelova I, Dhillon B, Wilhelm LJ, Goodwin SB, Berlin AM, Figueroa M, Freitag M, Hane JK, Henrissat B, Holman WH, Kodira CD, Martin J, Oliver RP, Robbertse B, Schackwitz W, Schwartz DC, Spatafora JW, Turgeon BG, Yandava C, Young S, Zhou S, Zeng Q, Grigoriev IV, Ma LJ, Ciuffetti LM Comparative genomics of a plant-pathogenic fungus, Pyrenophora tritici-repentis, reveals transduplication and the impact of repeat elements on pathogenicity and population divergence. G3 (Bethesda). 2013 Jan;3(1):41-63. | http://www.broadinstitute.org/annotation/genome/pyrenophora_tritici_repentis | 48 | 12169 | 12138 |
| *Dothistroma septosporum* | de Wit PJ, van der Burgt A, Okmen B, Stergiopoulos I, Abd-Elsalam KA, Aerts AL, Bahkali AH, Beenen HG, Chettri P, Cox MP, Datema E, de Vries RP, Dhillon B, Ganley AR, Griffiths SA, Guo Y, Hamelin RC, Henrissat B, Kabir MS, Jashni MK, Kema G, Klaubauf S, Lapidus A, Levasseur A, Lindquist E, Mehrabi R, Ohm RA, Owen TJ, Salamov A, Schwelm A, Schijlen E, Sun H, van den Burg HA, van Ham RC, Zhang S, Goodwin SB, Grigoriev IV, Collemare J, Bradshaw RE The genomes of the fungal plant pathogens Cladosporium fulvum and Dothistroma septosporum reveal adaptation to different hosts and lifestyles but also signatures of common ancestry. PLoS Genet. 2012;8(11):e1003088 | http://genome.jgi.doe.gov/Dotse1/Dotse1.home.html | 20 | 12580 | 11759 |
| *Pseudocercospora fijiensis (Mycosphaerella fijiensis)* | Ohm RA, Feau N, Henrissat B, Schoch CL, Horwitz BA, Barry KW, Condon BJ, Copeland AC, Dhillon B, Glaser F, Hesse CN, Kosti I, LaButti K, Lindquist EA, Lucas S, Salamov AA, Bradshaw RE, Ciuffetti L, Hamelin RC, Kema GH, Lawrence C, Scott JA, Spatafora JW, Turgeon BG, de Wit PJ, Zhong S, Goodwin SB, Grigoriev IV. Diverse lifestyles and strategies of plant pathogenesis encoded in the genomes of eighteen Dothideomycetes fungi. PLoS Pathog. 2012;8(12):e1003037. doi: 10.1371/journal.ppat.1003037. | ftp://ftp.ncbi.nlm.nih.gov/genomes/all/GCA_000340215.1_Mycfi2/ | 56 | 13130 | 9217 |
| *Zymoseptoria tritici (Mycosphaerella graminicola)* | Goodwin SB, M'barek SB, Dhillon B, Wittenberg AH, Crane CF, Hane JK, Foster AJ, Van der Lee TA, Grimwood J, Aerts A, Antoniw J, Bailey A, Bluhm B, Bowler J, Bristow J, van der Burgt A, Canto-Canche B, Churchill AC, Conde-Ferraez L, Cools HJ, Coutinho PM, Csukai M, Dehal P, De Wit P, Donzelli B, van de Geest HC, van Ham RC, Hammond-Kosack KE, Henrissat B, Kilian A, Kobayashi AK, Koopmann E, Kourmpetis Y, Kuzniar A, Lindquist E, Lombard V, Maliepaard C, Martins N, Mehrabi R, Nap JP, Ponomarenko A, Rudd JJ, Salamov A, Schmutz J, Schouten HJ, Shapiro H, Stergiopoulos I, Torriani SF, Tu H, de Vries RP, Waalwijk C, Ware SB, Wiebenga A, Zwiers LH, Oliver RP, Grigoriev IV, Kema GH Finished genome of the fungal wheat pathogen Mycosphaerella graminicola reveals dispensome structure, chromosome plasticity, and stealth pathogenesis. PLoS Genet. 2011 Jun;7(6):e1002070. | http://genome.jgi.doe.gov/Mycgr3/Mycgr3.home.html | 21 | 10933 | 9302 |
| *Passalora fulva* | de Wit PJ, van der Burgt A, Okmen B, Stergiopoulos I, Abd-Elsalam KA, Aerts AL, Bahkali AH, Beenen HG, Chettri P, Cox MP, Datema E, de Vries RP, Dhillon B, Ganley AR, Griffiths SA, Guo Y, Hamelin RC, Henrissat B, Kabir MS, Jashni MK, Kema G, Klaubauf S, Lapidus A, Levasseur A, Lindquist E, Mehrabi R, Ohm RA, Owen TJ, Salamov A, Schwelm A, Schijlen E, Sun H, van den Burg HA, van Ham RC, Zhang S, Goodwin SB, Grigoriev IV, Collemare J, Bradshaw RE The genomes of the fungal plant pathogens Cladosporium fulvum and Dothistroma septosporum reveal adaptation to different hosts and lifestyles but also signatures of common ancestry. PLoS Genet. 2012;8(11):e1003088 | http://genome.jgi.doe.gov/pages/dynamicOrganismDownload.jsf?organism=Clafu1 | 4865 | 14127 | 13655 |
| *Blumeria graminis* | Spanu PD, Abbott JC, Amselem J, Burgis TA, Soanes DM, Stüber K, Ver Loren van Themaat E, Brown JK, Butcher SA, Gurr SJ, Lebrun MH, Ridout CJ, Schulze-Lefert P, Talbot NJ, Ahmadinejad N, Ametz C, Barton GR, Benjdia M, Bidzinski P, Bindschedler LV, Both M, Brewer MT, Cadle-Davidson L, Cadle-Davidson MM, Collemare J, Cramer R, Frenkel O, Godfrey D, Harriman J, Hoede C, King BC, Klages S, Kleemann J, Knoll D, Koti PS, Kreplak J, López-Ruiz FJ, Lu X, Maekawa T, Mahanil S, Micali C, Milgroom MG, Montana G, Noir S, O'Connell RJ, Oberhaensli S, Parlange F, Pedersen C, Quesneville H, Reinhardt R, Rott M, Sacristán S, Schmidt SM, Schön M, Skamnioti P, Sommer H, Stephens A, Takahara H, Thordal-Christensen H, Vigouroux M, Wessling R, Wicker T, Panstruga R. Genome expansion and gene loss in powdery mildew fungi reveal tradeoffs in extreme parasitism. Science. 2010 Dec 10;330(6010):1543-6. | http://genome.jgi.doe.gov/pages/dynamicOrganismDownload.jsf?organism=Blugr1 | 6843 | 6470 | 6248 |
| *Erysiphe necator* | Jones L, Riaz S, Morales-Cruz A, Amrine KC, McGuire B, Gubler WD, Walker MA, Cantu D. Adaptive genomic structural variation in the grape powdery mildew pathogen, Erysiphe necator. BMC Genomics. 2014 Dec 9;15:1081. doi:10.1186/1471-2164-15-1081. PubMed PMID: 25487071; PubMed Central PMCID:PMC4298948. | http://www.ncbi.nlm.nih.gov/Traces/wgs/?val=JNVN01#contigs | 5935 | 6483 | 6483 |
| *Botrytis cinerea* | van Kan JA, Stassen JH, Mosbach A, van der Lee TA, Faino L, Farmer AD, Papasotiriou D, Zhou S, Seidl MF, Cottam E, Edel D, Hahn M, Schwartz DC, Dietrich RA, Widdison S, Scalliet G. A gapless genome sequence of the fungus Botrytis cinerea. Mol Plant Pathol. 2016 Feb 23. | http://fungi.ensembl.org/Botrytis_cinerea/Info/Index | 82 | 10351 | 10351 |
| *Sclerotinia sclerotiorum* | Derbyshire M, Denton-Giles M, Hegedus D, Rollins J, van Kan J, Seidl MF, Mbengue M, Navaud O, Raffaele S, Hammond-Kosack K, Heard S, Oliver RP. Improvement of the Sclerotinia sclerotiorum reference genome and gene models using PacBio and Illumina sequencing followed by characterisation of potential infection-related small RNAs. 13th European Conference on Fungal Genetics, Paris La Villette, April 3-6 2016. | https://www.ncbi.nlm.nih.gov/bioproject/PRJNA348385/ | 16 | 10953 | 10953 |
| *Oidiodendron maius* | Kohler A, Kuo A, Nagy LG, Morin E, Barry KW, Buscot F, Canback B, Choi C, Cichocki N, Clum A, Colpaert J, Copeland A, Costa MD, Dore J, Floudas D, Gay G, Girlanda M, Henrissat B, Herrmann S, Hess J, Hogberg N, Johansson T, Khouja HR, LaButti K, Lahrmann U, Levasseur A, Lindquist EA, Lipzen A, Marmeisse R, Martino E, Murat C, Ngan CY, Nehls U, Plett JM, Pringle A, Ohm RA, Perotto S, Peter M, Riley R, Rineau F, Ruytinx J, Salamov A, Shah F, Sun H, Tarkka M, Tritt A, Veneault-Fourrey C, Zuccaro A, Tunlid A, Grigoriev IV, Hibbett DS, Martin F Convergent losses of decay mechanisms and rapid turnover of symbiosis genes in mycorrhizal mutualists. Nat Genet. 2015 Apr;47(4):410-5. | http://genome.jgi.doe.gov/pages/dynamicOrganismDownload.jsf?organism=Oidma1 | 100 | 16703 | 15620 |
| *Pseudogymnoascus destructans (Geomyces destructans)* | Geomyces destructans Sequencing Project, Broad Institute of Harvard and MIT (http://www.broadinstitute.org/) | http://www.broadinstitute.org/annotation/genome/Geomyces_destructans/MultiHome.html | 1846 | 9075 | 8707 |
| *Magnaporthe oryzae* | Dean RA, Talbot NJ, Ebbole DJ, Farman ML, Mitchell TK, Orbach MJ, Thon M, Kulkarni R, Xu JR, Pan H, Read ND, Lee YH, Carbone I, Brown D, Oh YY, Donofrio N, Jeong JS, Soanes DM, Djonovic S, Kolomiets E, Rehmeyer C, Li W, Harding M, Kim S, Lebrun MH, Bohnert H, Coughlan S, Butler J, Calvo S, Ma LJ, Nicol R, Purcell S, Nusbaum C, Galagan JE, Birren BW. The genome sequence of the rice blast fungus Magnaporthe grisea. Nature. 2005 Apr 21;434(7036):980-6. | http://genome.jgi.doe.gov/pages/dynamicOrganismDownload.jsf?organism=Maggr1 | 8 | 11054 | 11052 |
| *Myceliophthora thermophila* | Berka RM, Grigoriev IV, Otillar R, Salamov A, Grimwood J, Reid I, Ishmael N, John T, Darmond C, Moisan MC, Henrissat B, Coutinho PM, Lombard V, Natvig DO, Lindquist E, Schmutz J, Lucas S, Harris P, Powlowski J, Bellemare A, Taylor D, Butler G, de Vries RP, Allijn IE, van den Brink J, Ushinsky S, Storms R, Powell AJ, Paulsen IT, Elbourne LD, Baker SE, Magnuson J, Laboissiere S, Clutterbuck AJ, Martinez D, Wogulis M, de Leon AL, Rey MW, Tsang A Comparative genomic analysis of the thermophilic biomass-degrading fungi Myceliophthora thermophila and Thielavia terrestris. Nat Biotechnol. 2011 Oct 2;29(10):922-7. | http://genome.jgi.doe.gov/Spoth2/Spoth2.download.html | 7 | 9110 | 8728 |
| *Chaetomium globosum* | Berka RM, Grigoriev IV, Otillar R, Salamov A, Grimwood J, Reid I, Ishmael N, John T, Darmond C, Moisan MC, Henrissat B, Coutinho PM, Lombard V, Natvig DO, Lindquist E, Schmutz J, Lucas S, Harris P, Powlowski J, Bellemare A, Taylor D, Butler G, de Vries RP, Allijn IE, van den Brink J, Ushinsky S, Storms R, Powell AJ, Paulsen IT, Elbourne LD, Baker SE, Magnuson J, Laboissiere S, Clutterbuck AJ, Martinez D, Wogulis M, de Leon AL, Rey MW, Tsang A Comparative genomic analysis of the thermophilic biomass-degrading fungi Myceliophthora thermophila and Thielavia terrestris. Nat Biotechnol. 2011 Oct 2;29(10):922-7. | http://genome.jgi.doe.gov/pages/dynamicOrganismDownload.jsf?organism=Chagl_1 | 37 | 11124 | 11084 |
| *Verticilium dahliae* | Faino L, Seidl MF, Datema E, van den Berg GC, Janssen A, Wittenberg AH, Thomma BP. Single-Molecule Real-Time Sequencing Combined with Optical Mapping Yields Completely Finished Fungal Genome. MBio. 2015 Aug 18;6(4). pii: e00936-15. | http://www.ncbi.nlm.nih.gov/genome/?term=GCA_000952015.1 | 55 | 10581 | 8071 |
| *Colletotrichum higginsianum* | O'Connell RJ, Thon MR, Hacquard S, Amyotte SG, Kleemann J, Torres MF, Damm U, Buiate EA, Epstein L, Alkan N, Altmuller J, Alvarado-Balderrama L, Bauser CA, Becker C, Birren BW, Chen Z, Choi J, Crouch JA, Duvick JP, Farman MA, Gan P, Heiman D, Henrissat B, Howard RJ, Kabbage M, Koch C, Kracher B, Kubo Y, Law AD, Lebrun MH, Lee YH, Miyara I, Moore N, Neumann U, Nordstrom K, Panaccione DG, Panstruga R, Place M, Proctor RH, Prusky D, Rech G, Reinhardt R, Rollins JA, Rounsley S, Schardl CL, Schwartz DC, Shenoy N, Shirasu K, Sikhakolli UR, Stuber K, Sukno SA, Sweigard JA, Takano Y, Takahara H, Trail F, van der Does HC, Voll LM, Will I, Young S, Zeng Q, Zhang J, Zhou S, Dickman MB, Schulze-Lefert P, Ver Loren van Themaat E, Ma LJ, Vaillancourt LJ. Lifestyle transitions in plant pathogenic Colletotrichum fungi deciphered by genome and transcriptome analyses. Nat Genet. 2012 Sep;44(9):1060-5. doi: 10.1038/ng.2372. Epub 2012 Aug 12. | http://genome.jgi.doe.gov/Colhi1/Colhi1.home.html | 10235 | 16172 | 12790 - 14913 ATG no STOP |
| *Colletotrichum graminicola* | Colletotrichum Sequencing Project, Broad Institute of Harvard and MIT (http://www.broadinstitute.org/) | http://www.broadinstitute.org/annotation/genome/colletotrichum_group/MultiHome.html | 654 | 12006 | 11849 |
| *Ophiocordyceps unilateralis* | de Bekker C, Ohm RA, Loreto RG, Sebastian A, Albert I, Merrow M, Brachmann A, Hughes DP. Gene expression during zombie ant biting behavior reflects the complexity underlying fungal parasitic behavioral manipulation. BMC Genomics. 2015 Aug 19;16:620. | http://www.ncbi.nlm.nih.gov/nuccore/LAZP00000000.1/ | 6790 | 7820 | 7820 |
| *Beauveria bassiana* | Xiao G, Ying SH, Zheng P, Wang ZL, Zhang S, Xie XQ, Shang Y, St Leger RJ, Zhao GP, Wang C, Feng MG Genomic perspectives on the evolution of fungal entomopathogenicity in Beauveria bassiana. Sci Rep. 2012;2:483. | http://genome.jgi.doe.gov/pages/dynamicOrganismDownload.jsf?organism=Beaba1 | 235 | 10364 | 10364 |
| *Fusarium graminearum* | Ma LJ, van der Does HC, Borkovich KA, Coleman JJ, Daboussi MJ, Di Pietro A, Dufresne M, Freitag M, Grabherr M, Henrissat B, Houterman PM, Kang S, Shim WB, Woloshuk C, Xie X, Xu JR, Antoniw J, Baker SE, Bluhm BH, Breakspear A, Brown DW, Butchko RA, Chapman S, Coulson R, Coutinho PM, Danchin EG, Diener A, Gale LR, Gardiner DM, Goff S, Hammond-Kosack KE, Hilburn K, Hua-Van A, Jonkers W, Kazan K, Kodira CD, Koehrsen M, Kumar L, Lee YH, Li L, Manners JM, Miranda-Saavedra D, Mukherjee M, Park G, Park J, Park SY, Proctor RH, Regev A, Ruiz-Roldan MC, Sain D, Sakthikumar S, Sykes S, Schwartz DC, Turgeon BG, Wapinski I, Yoder O, Young S, Zeng Q, Zhou S, Galagan J, Cuomo CA, Kistler HC, Rep M. Comparative genomics reveals mobile pathogenicity chromosomes in Fusarium. Nature. 2010 Mar 18;464(7287):367-73. | http://genome.jgi.doe.gov/pages/dynamicOrganismDownload.jsf?organism=Fusgr1 | 31 | 13322 | 13287 |
| *Metarhizium acridum* | Gao Q, Jin K, Ying SH, Zhang Y, Xiao G, Shang Y, Duan Z, Hu X, Xie XQ, Zhou G, Peng G, Luo Z, Huang W, Wang B, Fang W, Wang S, Zhong Y, Ma LJ, St Leger RJ, Zhao GP, Pei Y, Feng MG, Xia Y, Wang C Genome sequencing and comparative transcriptomics of the model entomopathogenic fungi Metarhizium anisopliae and M. acridum. PLoS Genet. 2011 Jan 6;7(1):e1001264. | http://genome.jgi.doe.gov/pages/dynamicOrganismDownload.jsf?organism=Metac1 | 241 | 9849 | 9845 |
