# Supplementary material for: Codon optimization underpins generalist parasitism in fungi
Source: eLife. 2017 Feb 3;6:e22472. doi: 10.7554/eLife.22472 (PMC5315462; doi:10.7554/eLife.22472)
Supplement: Figure 4—source data 1. — DOI: http://dx.doi.org/10.7554/eLife.22472.012 [file elife-22472-fig4-data1.docx]

**Figure 4 – source data 1.** Codon statistics for *S. sclerotiorum* genome.

| Codon | AA | Fraction | Frequency | Number | Synonym. mutation rate(%) | Non syn.  mutation rate(%) | Adjusted Non syn. Rate(%) | tRNAs | % tRNA pool  (wobble) | optimal? |
| --- | --- | --- | --- | --- | --- | --- | --- | --- | --- | --- |
| GCA | A | 0.308 | 23.539 | 125745 | 0.172571 | 0.341167 | 0.170583 | 4 | 2.083333 | yes |
| GCC | A | 0.206 | 15.729 | 84025 | 0.323713 | 0.376079 | 0.188039 | 0 | 1.822917 | - |
| GCG | A | 0.15 | 11.461 | 61223 | 0.393643 | 0.429577 | 0.214789 | 2 | 1.041667 | no |
| GCT | A | 0.336 | 25.663 | 137091 | 0.190384 | 0.302719 | 0.151359 | 7 | 1.822917 | - |
| TGC | C | 0.426 | 5.024 | 26836 | 0.227307 | 0.450887 | 0.056361 | 3 | 0.78125 | no |
| TGT | C | 0.574 | 6.764 | 36133 | 0.202031 | 0.470484 | 0.058811 | 0 | 0.78125 | yes |
| GAC | D | 0.287 | 15.867 | 84761 | 0.338599 | 0.423544 | 0.052943 | 8 | 2.083333 | no |
| GAT | D | 0.713 | 39.362 | 210269 | 0.116042 | 0.269655 | 0.033707 | 0 | 2.083333 | yes |
| GAA | E | 0.578 | 38.035 | 203181 | 0.134363 | 0.286444 | 0.035806 | 5 | 2.604167 | no |
| GAG | E | 0.422 | 27.823 | 148627 | 0.19041 | 0.257019 | 0.032127 | 7 | 3.645833 | yes |
| TTC | F | 0.51 | 18.902 | 100976 | 0.204999 | 0.217874 | 0.027234 | 6 | 1.5625 | yes |
| TTT | F | 0.49 | 18.144 | 96924 | 0.200157 | 0.259998 | 0.0325 | 0 | 1.5625 | no |
| GGA | G | 0.348 | 23.468 | 125365 | 0.200215 | 0.331033 | 0.165517 | 6 | 3.125 | yes |
| GGC | G | 0.184 | 12.415 | 66322 | 0.35584 | 0.384488 | 0.192244 | 7 | 1.822917 | - |
| GGG | G | 0.143 | 9.619 | 51385 | 0.492362 | 0.474847 | 0.237423 | 1 | 0.520833 | no |
| GGT | G | 0.325 | 21.877 | 116868 | 0.209638 | 0.319164 | 0.159582 | 0 | 1.822917 | - |
| CAC | H | 0.356 | 8.119 | 43371 | 0.272071 | 0.500334 | 0.062542 | 4 | 1.041667 | no |
| CAT | H | 0.644 | 14.703 | 78541 | 0.133688 | 0.421436 | 0.052679 | 0 | 1.041667 | yes |
| ATA | I | 0.212 | 11.749 | 62765 | 0.076476 | 0.742452 | 0.212129 | 1 | 0.520833 | no |
| ATC | I | 0.356 | 19.721 | 105349 | 0.288565 | 0.29426 | 0.084074 | 0 | 1.5625 | - |
| ATT | I | 0.432 | 23.964 | 128014 | 0.190604 | 0.350743 | 0.100212 | 6 | 1.5625 | yes |
| AAA | K | 0.512 | 28.345 | 151418 | 0.134726 | 0.321626 | 0.040203 | 3 | 3.125 | no |
| AAG | K | 0.488 | 26.976 | 144104 | 0.147116 | 0.275495 | 0.034437 | 10 | 3.645833 | yes |
| CTA | L | 0.112 | 9.497 | 50733 | 0.528256 | 0.291723 | 0.364654 | 2 | 1.041667 | - |
| CTC | L | 0.197 | 16.797 | 89730 | 0.248523 | 0.171626 | 0.214532 | 0 | 1.302083 | - |
| CTG | L | 0.089 | 7.603 | 40617 | 0.566265 | 0.253588 | 0.316985 | 1 | 0.520833 | no |
| CTT | L | 0.226 | 19.238 | 102767 | 0.160557 | 0.206292 | 0.257865 | 5 | 1.302083 | - |
| TTA | L | 0.156 | 13.281 | 70944 | 0.41723 | 0.177605 | 0.222006 | 1 | 0.520833 | - |
| TTG | L | 0.22 | 18.74 | 100106 | 0.297684 | 0.156834 | 0.196042 | 5 | 2.604167 | yes |
| ATG | M | 1 | 22.023 | 117646 | 0 | 0.258402 | 0.258402 | 7 | 3.645833 | - |
| AAC | N | 0.375 | 16 | 85470 | 0.30888 | 0.34164 | 0.042705 | 6 | 1.5625 | no |
| AAT | N | 0.625 | 26.653 | 142377 | 0.146793 | 0.332919 | 0.041615 | 0 | 1.5625 | yes |
| CCA | P | 0.376 | 21.648 | 115642 | 0.175542 | 0.37789 | 0.188945 | 6 | 3.125 | yes |
| CCC | P | 0.175 | 10.06 | 53741 | 0.403789 | 0.360991 | 0.180495 | 0 | 1.041667 | - |
| CCG | P | 0.133 | 7.687 | 41063 | 0.533327 | 0.372598 | 0.186299 | 1 | 0.520833 | no |
| CCT | P | 0.316 | 18.242 | 97448 | 0.205238 | 0.298621 | 0.14931 | 3 | 1.041667 | - |
| CAA | Q | 0.68 | 26.393 | 140992 | 0.103552 | 0.195047 | 0.024381 | 5 | 2.604167 | yes |
| CAG | Q | 0.32 | 12.414 | 66314 | 0.233736 | 0.242784 | 0.030348 | 2 | 1.041667 | no |
| AGA | R | 0.283 | 16.2 | 86538 | 0.160623 | 0.333957 | 0.417447 | 6 | 3.125 | yes |
| AGG | R | 0.155 | 8.876 | 47416 | 0.316349 | 0.499831 | 0.624789 | 2 | 1.041667 | - |
| CGA | R | 0.202 | 11.583 | 61874 | 0.161619 | 0.244044 | 0.305055 | 4 | 2.083333 | - |
| CGC | R | 0.127 | 7.243 | 38690 | 0.266219 | 0.356681 | 0.445852 | 0 | 2.083333 | - |
| CGG | R | 0.069 | 3.947 | 21087 | 0.39835 | 0.611751 | 0.764689 | 1 | 0.520833 | no |
| CGT | R | 0.164 | 9.392 | 50170 | 0.147499 | 0.452462 | 0.565577 | 5 | 2.604167 | - |
| AGC | S | 0.121 | 10.558 | 56399 | 0.246458 | 0.404262 | 0.505328 | 5 | 1.302083 | no |
| AGT | S | 0.18 | 15.694 | 83837 | 0.141942 | 0.446104 | 0.55763 | 0 | 1.302083 | - |
| TCA | S | 0.206 | 17.936 | 95815 | 0.19308 | 0.289099 | 0.361373 | 3 | 1.5625 | yes |
| TCC | S | 0.158 | 13.786 | 73646 | 0.362545 | 0.320452 | 0.400565 | 0 | 1.5625 | - |
| TCG | S | 0.118 | 10.299 | 55015 | 0.376261 | 0.274471 | 0.343088 | 3 | 1.5625 | - |
| TCT | S | 0.216 | 18.804 | 100452 | 0.229961 | 0.272767 | 0.340959 | 6 | 1.5625 | - |
| ACA | T | 0.306 | 18.871 | 100810 | 0.212281 | 0.448368 | 0.224184 | 3 | 1.5625 | yes |
| ACC | T | 0.24 | 14.761 | 78855 | 0.3703 | 0.35635 | 0.178175 | 0 | 1.5625 | - |
| ACG | T | 0.156 | 9.604 | 51302 | 0.460021 | 0.450275 | 0.225137 | 1 | 0.520833 | no |
| ACT | T | 0.298 | 18.368 | 98119 | 0.251735 | 0.415822 | 0.207911 | 6 | 1.5625 | - |
| GTA | V | 0.196 | 11.093 | 59258 | 0.261568 | 0.538324 | 0.269162 | 1 | 0.520833 | no |
| GTC | V | 0.254 | 14.374 | 76783 | 0.272196 | 0.423271 | 0.211635 | 0 | 2.083333 | - |
| GTG | V | 0.19 | 10.79 | 57638 | 0.288004 | 0.525695 | 0.262847 | 2 | 1.041667 | - |
| GTT | V | 0.36 | 20.399 | 108970 | 0.18629 | 0.456089 | 0.228044 | 8 | 2.083333 | yes |
| TGG | W | 1 | 13.729 | 73339 | 0 | 0.06954 | 0.06954 | 6 | 1.5625 | - |
| TAC | Y | 0.423 | 11.884 | 63486 | 0.294553 | 0.187443 | 0.02343 | 5 | 1.302083 | no |
| TAT | Y | 0.577 | 16.183 | 86449 | 0.211686 | 0.300755 | 0.037594 | 0 | 1.302083 | yes |
| TAA | * | 0.316 | 0.659 | 3522 | 0.397501 | 0.170358 | 0.048674 | 0 | 0.520833 | - |
| TAG | * | 0.303 | 0.631 | 3373 | 0.20753 | 0.326119 | 0.093177 | 0 | 0.520833 | - |
| TGA | * | 0.381 | 0.793 | 4235 | 0.118064 | 0.188902 | 0.053972 | 0 | 1.5625 | - |

Codon statistics for *Z. tritici* genome.

| Codon | AA | Fraction | Frequency | Number | Synonym. mutation rate(%) | Non syn. mutation rate(%) | Adjusted Non syn. rate(%) | tRNAs | % tRNA pool  (wobble) | optimal? |
| --- | --- | --- | --- | --- | --- | --- | --- | --- | --- | --- |
| GCA | A | 0.213 | 19.31 | 91754 | 2.765002 | 1.205397 | 0.602699 | 0 | 0.454545 | no |
| GCC | A | 0.3 | 27.173 | 129116 | 1.536603 | 1.013043 | 0.506521 | 0 | 0.909091 | - |
| GCG | A | 0.266 | 24.078 | 114409 | 2.275171 | 1.178229 | 0.589114 | 1 | 0.454545 | - |
| GCT | A | 0.221 | 20.018 | 95119 | 2.188837 | 0.900977 | 0.450488 | 2 | 0.909091 | yes |
| TGC | C | 0.661 | 8.373 | 39785 | 1.721754 | 1.080809 | 0.135101 | 0 | 25.45455 | no |
| TGT | C | 0.339 | 4.3 | 20431 | 3.161862 | 1.218736 | 0.152342 | 56 | 25.45455 | yes |
| GAC | D | 0.579 | 33.539 | 159365 | 2.671226 | 0.954413 | 0.119302 | 0 | 0.242424 | yes |
| GAT | D | 0.421 | 24.418 | 116027 | 3.479363 | 0.762753 | 0.095344 | 0 | 0.242424 | no |
| GAA | E | 0.367 | 23.014 | 109354 | 1.053459 | 0.634636 | 0.07933 | 0 | 0.454545 | no |
| GAG | E | 0.633 | 39.69 | 188595 | 0.49312 | 0.775206 | 0.096901 | 1 | 0.454545 | yes |
| TTC | F | 0.738 | 26.791 | 127303 | 0.604071 | 0.466603 | 0.058325 | 0 | 0.242424 | yes |
| TTT | F | 0.262 | 9.488 | 45082 | 1.770108 | 0.314982 | 0.039373 | 0 | 0.242424 | no |
| GGA | G | 0.264 | 18.519 | 87998 | 2.437555 | 0.32728 | 0.16364 | 0 | 0.454545 | no |
| GGC | G | 0.394 | 27.566 | 130985 | 3.207237 | 0.378669 | 0.189335 | 3 | 1.363636 | yes |
| GGG | G | 0.126 | 8.822 | 41918 | 2.127964 | 0.45088 | 0.22544 | 1 | 0.909091 | - |
| GGT | G | 0.216 | 15.12 | 71843 | 5.374219 | 0.463511 | 0.231755 | 0 | 1.363636 | - |
| CAC | H | 0.604 | 14.745 | 70062 | 2.012503 | 1.40447 | 0.175559 | 2 | 0.909091 | yes |
| CAT | H | 0.396 | 9.656 | 45883 | 2.794063 | 1.453697 | 0.181712 | 0 | 0.909091 | no |
| ATA | I | 0.094 | 4.398 | 20898 | 2.43564 | 3.081635 | 0.880467 | 0 | 1.363636 | yes |
| ATC | I | 0.631 | 29.385 | 139627 | 1.297027 | 1.214665 | 0.347047 | 0 | 0.242424 | - |
| ATT | I | 0.275 | 12.815 | 60895 | 2.402496 | 1.23327 | 0.352363 | 0 | 0.242424 | no |
| AAA | K | 0.304 | 14.864 | 70629 | 0.824024 | 0.547934 | 0.068492 | 0 | 0.454545 | no |
| AAG | K | 0.696 | 34.076 | 161919 | 0.353263 | 0.508896 | 0.063612 | 1 | 0.454545 | yes |
| CTA | L | 0.065 | 5.657 | 26878 | 6.276509 | 0.636208 | 0.79526 | 0 | 0.454545 | - |
| CTC | L | 0.342 | 29.948 | 142305 | 1.603598 | 0.466603 | 0.583254 | 0 | 0.242424 | - |
| CTG | L | 0.243 | 21.324 | 101325 | 3.208488 | 0.43622 | 0.545275 | 1 | 0.454545 | yes |
| CTT | L | 0.134 | 11.756 | 55859 | 2.146476 | 0.443975 | 0.554969 | 0 | 0.242424 | no |
| TTA | L | 0.027 | 2.396 | 11387 | 6.542549 | 1.176781 | 1.470976 | 0 | 0.454545 | - |
| TTG | L | 0.189 | 16.581 | 78788 | 1.931766 | 0.483576 | 0.60447 | 1 | 0.454545 | - |
| ATG | M | 1 | 22.451 | 106678 | 0 | 1.374229 | 1.374229 | 3 | 1.363636 | - |
| AAC | N | 0.638 | 22.714 | 107930 | 2.989901 | 0.765311 | 0.095664 | 3 | 1.363636 | yes |
| AAT | N | 0.362 | 12.874 | 61171 | 4.644358 | 0.866424 | 0.108303 | 0 | 1.363636 | no |
| CCA | P | 0.264 | 15.287 | 72641 | 2.999683 | 0.476315 | 0.238158 | 0 | 0.454545 | - |
| CCC | P | 0.231 | 13.401 | 63677 | 1.212369 | 0.502536 | 0.251268 | 0 | 0.454545 | - |
| CCG | P | 0.286 | 16.593 | 78843 | 2.743427 | 0.43631 | 0.218155 | 1 | 0.454545 | yes |
| CCT | P | 0.218 | 12.657 | 60141 | 2.171563 | 0.427329 | 0.213665 | 1 | 0.454545 | no |
| CAA | Q | 0.444 | 17.821 | 84681 | 0.849069 | 0.767587 | 0.095948 | 0 | 0.242424 | no |
| CAG | Q | 0.556 | 22.316 | 106039 | 0.700686 | 0.949651 | 0.118706 | 0 | 0.242424 | yes |
| AGA | R | 0.128 | 7.931 | 37686 | 1.228573 | 0.875657 | 1.094571 | 6 | 5.454545 | yes |
| AGG | R | 0.136 | 8.389 | 39861 | 1.713454 | 0.654775 | 0.818469 | 1 | 0.909091 | - |
| CGA | R | 0.199 | 12.314 | 58510 | 2.196206 | 0.42215 | 0.527688 | 1 | 0.909091 | - |
| CGC | R | 0.274 | 16.944 | 80514 | 2.266687 | 0.681869 | 0.852336 | 0 | 0.909091 | - |
| CGG | R | 0.131 | 8.12 | 38584 | 2.661725 | 0.57796 | 0.72245 | 1 | 0.909091 | no |
| CGT | R | 0.13 | 8.054 | 38270 | 4.222629 | 0.95375 | 1.192187 | 2 | 1.818182 | - |
| AGC | S | 0.213 | 16.812 | 79886 | 1.907718 | 0.900033 | 1.125041 | 0 | 0.242424 | - |
| AGT | S | 0.117 | 9.238 | 43894 | 3.091539 | 1.214289 | 1.517861 | 0 | 0.227273 | no |
| TCA | S | 0.119 | 9.427 | 44792 | 3.319789 | 0.94883 | 1.186038 | 2 | 1.818182 | - |
| TCC | S | 0.232 | 18.315 | 87029 | 1.484563 | 0.934171 | 1.167714 | 0 | 0.454545 | - |
| TCG | S | 0.187 | 14.795 | 70303 | 2.287242 | 0.70694 | 0.883675 | 2 | 1.818182 | yes |
| TCT | S | 0.133 | 10.501 | 49899 | 2.138319 | 0.909838 | 1.137297 | 1 | 0.454545 | - |
| ACA | T | 0.209 | 12.683 | 60265 | 2.799303 | 1.440305 | 0.720153 | 1 | 0.227273 | - |
| ACC | T | 0.334 | 20.244 | 96192 | 1.518837 | 1.89101 | 0.945505 | 0 | 0.227273 | - |
| ACG | T | 0.268 | 16.213 | 77038 | 2.567564 | 1.342195 | 0.671097 | 1 | 0.909091 | yes |
| ACT | T | 0.188 | 11.414 | 54237 | 2.496451 | 1.416008 | 0.708004 | 0 | 0.227273 | no |
| GTA | V | 0.088 | 5.379 | 25561 | 5.457533 | 2.542937 | 1.271468 | 0 | 0.909091 | - |
| GTC | V | 0.429 | 26.299 | 124963 | 1.868553 | 1.35 | 0.675 | 0 | 0.454545 | - |
| GTG | V | 0.321 | 19.666 | 93445 | 2.289047 | 1.335545 | 0.667772 | 2 | 0.909091 | yes |
| GTT | V | 0.163 | 9.968 | 47363 | 2.485062 | 1.534953 | 0.767477 | 1 | 0.454545 | no |
| TGG | W | 1 | 14.582 | 69288 | 0 | 0.4171 | 0.4171 | 0 | 0.242424 | - |
| TAC | Y | 0.706 | 18.884 | 89733 | 2.049413 | 1.128905 | 0.141113 | 1 | 0.909091 | no |
| TAT | Y | 0.294 | 7.873 | 37410 | 3.988238 | 1.264368 | 0.158046 | 7 | 6.363636 | yes |
| TAA | * | 0.13 | 0.264 | 1254 | 2.392344 | 2.472089 | 0.706311 | 0 | 0.242424 | - |
| TAG | * | 0.297 | 0.6 | 2851 | 0.140302 | 1.999298 | 0.571228 | 0 | 0.242424 | - |
| TGA | * | 0.573 | 1.159 | 5507 | 0.435809 | 1.307427 | 0.373551 | 0 | 0.242424 | - |
