# Supplementary material for: Codon optimization underpins generalist parasitism in fungi
Source: eLife. 2017 Feb 3;6:e22472. doi: 10.7554/eLife.22472 (PMC5315462; doi:10.7554/eLife.22472)
Supplement: Figure 4—source data 2. — Ref. indicates codons in the reference genome (isolate 1980 for S. sclerotiorum and isolate IPO323 for Z. tritici) tog ether with their total number, Var. indicates variant codons. DOI: http://dx.doi.org/10.7554/eLife.22472.013 [file elife-22472-fig4-data2.docx]

**Figure 4 – source data 2.** Frequency of codon substitutions in *S. sclerotiorum* population (as % of all codons). Ref. indicates codons in the reference genome (isolate 1980) together with their total number, Var. indicates variant codons.

|  |  | **Var.** |  |  |  |  |  |  |  |  |  |  |  |  |  |  |  |  |  |  |  |  |  |  |  |  |  |  |  |  |  |  |
| --- | --- | --- | --- | --- | --- | --- | --- | --- | --- | --- | --- | --- | --- | --- | --- | --- | --- | --- | --- | --- | --- | --- | --- | --- | --- | --- | --- | --- | --- | --- | --- | --- |
| Ref. |  | **GCA** | **GCC** | **GCG** | **GCT** | **TGC** | **TGT** | **GAC** | **GAT** | **GAA** | **GAG** | **TTC** | **TTT** | **GGA** | **GGC** | **GGG** | **GGT** | **CAC** | **CAT** | **ATA** | **ATC** | **ATT** | **AAA** | **AAG** | **CTA** | **CTC** | **CTG** | **CTT** | **TTA** | **TTG** | **ATG** | **AAC** |
| 125745 | GCA | 0 | 0.019086 | 0.104974 | 0.0167 | 0 | 0 | 0 | 0 | 0.010338 | 0 | 0 | 0 | 0.011929 | 0 | 0 | 0 | 0 | 0 | 0 | 0 | 0 | 0 | 0 | 0 | 0 | 0 | 0 | 0.000795 | 0 | 0 | 0 |
| 84025 | GCC | 0.030943 | 0 | 0.023802 | 0.19518 | 0 | 0 | 0.005951 | 0.00119 | 0 | 0 | 0 | 0 | 0 | 0.013091 | 0 | 0 | 0 | 0 | 0 | 0 | 0 | 0 | 0 | 0 | 0 | 0 | 0 | 0 | 0 | 0 | 0 |
| 61223 | GCG | 0.223772 | 0.027767 | 0 | 0.039201 | 0 | 0 | 0 | 0.003267 | 0 | 0.011434 | 0 | 0 | 0 | 0 | 0.011434 | 0 | 0 | 0 | 0 | 0 | 0 | 0 | 0 | 0 | 0 | 0 | 0.001633 | 0 | 0.001633 | 0 | 0 |
| 137091 | GCT | 0.008024 | 0.121817 | 0.011671 | 0 | 0 | 0 | 0 | 0.005106 | 0 | 0 | 0 | 0 | 0 | 0 | 0 | 0.011671 | 0 | 0 | 0 | 0 | 0 | 0 | 0 | 0 | 0 | 0 | 0 | 0 | 0 | 0 | 0 |
| 26836 | TGC | 0 | 0.029811 | 0 | 0 | 0 | 0.171412 | 0 | 0 | 0 | 0 | 0.018632 | 0 | 0 | 0.011179 | 0 | 0 | 0.007453 | 0 | 0 | 0 | 0 | 0 | 0 | 0 | 0 | 0 | 0 | 0 | 0 | 0 | 0 |
| 36133 | TGT | 0 | 0 | 0 | 0 | 0.157751 | 0 | 0 | 0 | 0 | 0 | 0 | 0.019373 | 0 | 0 | 0 | 0.033211 | 0 | 0 | 0 | 0 | 0 | 0 | 0 | 0 | 0 | 0 | 0 | 0 | 0 | 0 | 0 |
| 84761 | GAC | 0 | 0 | 0 | 0 | 0 | 0 | 0 | 0.239497 | 0.048371 | 0.017697 | 0 | 0 | 0 | 0.103821 | 0 | 0 | 0.003539 | 0 | 0 | 0 | 0 | 0.00118 | 0.00118 | 0 | 0 | 0 | 0 | 0 | 0 | 0 | 0.089664 |
| 210269 | GAT | 0 | 0 | 0 | 0.005707 | 0 | 0 | 0.087983 | 0 | 0.009987 | 0.015694 | 0 | 0 | 0 | 0 | 0 | 0.041376 | 0 | 0.010463 | 0.000951 | 0 | 0 | 0 | 0 | 0 | 0 | 0 | 0 | 0 | 0.004756 | 0 | 0 |
| 203181 | GAA | 0.007383 | 0 | 0 | 0 | 0 | 0 | 0.019687 | 0.013289 | 0 | 0.097942 | 0 | 0 | 0.062998 | 0 | 0 | 0 | 0 | 0 | 0 | 0.000492 | 0 | 0.071857 | 0.002953 | 0 | 0 | 0 | 0 | 0 | 0 | 0 | 0.001477 |
| 148627 | GAG | 0 | 0 | 0.003364 | 0 | 0 | 0 | 0.015475 | 0.017493 | 0.134565 | 0 | 0 | 0 | 0.000673 | 0 | 0.04037 | 0 | 0 | 0 | 0 | 0.000673 | 0 | 0.000673 | 0.055844 | 0 | 0 | 0 | 0 | 0.000673 | 0 | 0 | 0 |
| 100976 | TTC | 0 | 0 | 0 | 0 | 0.00099 | 0 | 0 | 0.002971 | 0 | 0 | 0 | 0.146569 | 0 | 0 | 0 | 0 | 0 | 0 | 0 | 0.003961 | 0 | 0 | 0 | 0 | 0.047536 | 0 | 0 | 0.009903 | 0.019807 | 0 | 0 |
| 96924 | TTT | 0 | 0 | 0 | 0.002063 | 0 | 0.007222 | 0 | 0 | 0 | 0 | 0.147538 | 0 | 0 | 0 | 0 | 0.002063 | 0 | 0 | 0 | 0 | 0.009286 | 0 | 0 | 0.002063 | 0.002063 | 0 | 0.052619 | 0.002063 | 0.02992 | 0 | 0 |
| 125365 | GGA | 0.011167 | 0 | 0 | 0 | 0 | 0 | 0 | 0.000798 | 0.11646 | 0 | 0 | 0 | 0 | 0.01356 | 0.121246 | 0.012763 | 0 | 0 | 0 | 0 | 0 | 0.000798 | 0.000798 | 0 | 0 | 0 | 0 | 0 | 0 | 0 | 0 |
| 66322 | GGC | 0 | 0.016586 | 0.003016 | 0 | 0.012062 | 0 | 0.10253 | 0 | 0 | 0 | 0 | 0 | 0.019601 | 0 | 0.022617 | 0.224661 | 0 | 0 | 0 | 0 | 0 | 0 | 0 | 0 | 0 | 0 | 0 | 0 | 0 | 0 | 0 |
| 51385 | GGG | 0 | 0 | 0.033084 | 0 | 0 | 0 | 0 | 0 | 0.001946 | 0.144011 | 0 | 0 | 0.307483 | 0.025299 | 0 | 0.052545 | 0 | 0 | 0.005838 | 0 | 0 | 0 | 0.003892 | 0 | 0 | 0 | 0 | 0 | 0 | 0 | 0 |
| 116868 | GGT | 0 | 0 | 0 | 0.011979 | 0 | 0.013691 | 0 | 0.080433 | 0 | 0 | 0 | 0.000856 | 0.022247 | 0.110381 | 0.017113 | 0 | 0 | 0 | 0 | 0 | 0 | 0 | 0 | 0 | 0 | 0 | 0 | 0 | 0 | 0 | 0 |
| 43371 | CAC | 0 | 0 | 0 | 0 | 0 | 0 | 0.006917 | 0.004611 | 0 | 0 | 0 | 0.002306 | 0 | 0 | 0 | 0 | 0 | 0.200595 | 0 | 0 | 0 | 0 | 0 | 0 | 0 | 0 | 0 | 0 | 0 | 0 | 0.01614 |
| 78541 | CAT | 0 | 0 | 0 | 0 | 0 | 0 | 0.005093 | 0.019098 | 0 | 0 | 0 | 0 | 0.001273 | 0 | 0 | 0 | 0.100584 | 0 | 0 | 0 | 0 | 0.001273 | 0 | 0 | 0 | 0 | 0.011459 | 0 | 0 | 0 | 0 |
| 62765 | ATA | 0 | 0 | 0 | 0 | 0 | 0 | 0 | 0.003186 | 0 | 0 | 0 | 0 | 0 | 0 | 0 | 0.003186 | 0 | 0 | 0 | 0.043018 | 0.015932 | 0.007966 | 0 | 0.017526 | 0 | 0 | 0 | 0.009559 | 0 | 0.157731 | 0 |
| 105349 | ATC | 0 | 0 | 0 | 0.000949 | 0 | 0.001898 | 0 | 0 | 0 | 0 | 0.006645 | 0 | 0 | 0 | 0 | 0.000949 | 0 | 0 | 0.028477 | 0 | 0.169911 | 0 | 0 | 0 | 0.007594 | 0 | 0 | 0 | 0 | 0.019934 | 0.003797 |
| 128014 | ATT | 0.000781 | 0 | 0 | 0 | 0 | 0 | 0 | 0 | 0 | 0 | 0 | 0.003125 | 0 | 0 | 0 | 0.000781 | 0 | 0 | 0.014842 | 0.121081 | 0 | 0 | 0 | 0 | 0 | 0 | 0.034371 | 0 | 0 | 0.009374 | 0 |
| 151418 | AAA | 0 | 0 | 0 | 0 | 0 | 0 | 0.001321 | 0 | 0.087836 | 0 | 0 | 0 | 0.001321 | 0 | 0 | 0 | 0 | 0 | 0.003302 | 0 | 0.00066 | 0 | 0.094441 | 0.00066 | 0 | 0 | 0 | 0.001321 | 0 | 0 | 0.018492 |
| 144104 | AAG | 0 | 0 | 0 | 0 | 0 | 0 | 0 | 0 | 0.002776 | 0.064537 | 0 | 0 | 0 | 0 | 0 | 0 | 0 | 0 | 0 | 0 | 0 | 0.101316 | 0 | 0 | 0 | 0 | 0 | 0 | 0 | 0.004858 | 0.010409 |
| 50733 | CTA | 0 | 0 | 0 | 0 | 0 | 0 | 0 | 0 | 0 | 0 | 0 | 0 | 0 | 0 | 0 | 0 | 0 | 0 | 0.015769 | 0 | 0 | 0 | 0 | 0 | 0.045335 | 0.147833 | 0.015769 | 0.16163 | 0.005913 | 0 | 0.001971 |
| 89730 | CTC | 0 | 0 | 0 | 0 | 0 | 0 | 0 | 0 | 0 | 0 | 0.053494 | 0.004458 | 0 | 0 | 0 | 0 | 0.002229 | 0 | 0 | 0.014488 | 0 | 0 | 0 | 0.025632 | 0 | 0.014488 | 0.124819 | 0 | 0 | 0.001114 | 0 |
| 40617 | CTG | 0 | 0 | 0 | 0 | 0 | 0 | 0 | 0 | 0 | 0 | 0 | 0 | 0 | 0 | 0 | 0 | 0 | 0 | 0 | 0 | 0 | 0 | 0 | 0.160032 | 0.034468 | 0 | 0.017234 | 0.002462 | 0.174804 | 0.039392 | 0 |
| 102767 | CTT | 0 | 0 | 0 | 0 | 0.001946 | 0 | 0 | 0 | 0 | 0 | 0.001946 | 0.026273 | 0 | 0 | 0 | 0 | 0 | 0.003892 | 0.001946 | 0 | 0.022381 | 0 | 0 | 0.008758 | 0.107038 | 0.010704 | 0 | 0 | 0 | 0 | 0 |
| 70944 | TTA | 0 | 0 | 0 | 0 | 0 | 0 | 0 | 0.004229 | 0 | 0 | 0.007048 | 0.002819 | 0 | 0 | 0 | 0 | 0 | 0 | 0.012686 | 0 | 0 | 0 | 0 | 0.118403 | 0.00141 | 0.002819 | 0 | 0 | 0.197339 | 0 | 0 |
| 100106 | TTG | 0 | 0 | 0 | 0 | 0 | 0 | 0 | 0 | 0 | 0 | 0.013985 | 0.019979 | 0 | 0 | 0.000999 | 0 | 0 | 0 | 0.000999 | 0 | 0 | 0 | 0 | 0 | 0 | 0.074921 | 0 | 0.137854 | 0 | 0.006993 | 0 |
| 117646 | ATG | 0 | 0 | 0.00085 | 0 | 0 | 0 | 0 | 0 | 0 | 0 | 0.0017 | 0 | 0 | 0 | 0 | 0 | 0.00085 | 0 | 0.111351 | 0.0085 | 0.0289 | 0 | 0.0034 | 0 | 0 | 0.0085 | 0 | 0.0017 | 0.00765 | 0 | 0 |
| 85470 | AAC | 0.00117 | 0 | 0 | 0 | 0 | 0 | 0.06552 | 0.00117 | 0.00234 | 0.00117 | 0 | 0 | 0.00117 | 0 | 0 | 0 | 0.00819 | 0 | 0.00117 | 0.00234 | 0 | 0.04563 | 0.02691 | 0 | 0 | 0 | 0 | 0 | 0 | 0 | 0 |
| 142377 | AAT | 0.001405 | 0 | 0 | 0 | 0 | 0 | 0 | 0.08639 | 0.003512 | 0.000702 | 0 | 0 | 0 | 0 | 0.001405 | 0 | 0 | 0.020368 | 0 | 0 | 0.006321 | 0.008428 | 0.02669 | 0 | 0 | 0 | 0 | 0 | 0 | 0 | 0.106759 |
| 115642 | CCA | 0.010377 | 0 | 0.000865 | 0 | 0 | 0 | 0 | 0 | 0 | 0 | 0.000865 | 0 | 0 | 0 | 0 | 0 | 0 | 0 | 0 | 0 | 0 | 0 | 0 | 0.089933 | 0 | 0 | 0 | 0 | 0 | 0 | 0 |
| 53741 | CCC | 0 | 0.014886 | 0 | 0 | 0 | 0 | 0 | 0 | 0 | 0 | 0 | 0 | 0 | 0 | 0 | 0 | 0.009304 | 0 | 0 | 0 | 0 | 0 | 0 | 0 | 0.093039 | 0 | 0 | 0 | 0 | 0 | 0 |
| 41063 | CCG | 0 | 0 | 0.004871 | 0 | 0 | 0 | 0 | 0 | 0 | 0 | 0 | 0 | 0 | 0 | 0 | 0 | 0 | 0 | 0 | 0 | 0 | 0 | 0 | 0 | 0 | 0.092541 | 0 | 0 | 0 | 0 | 0 |
| 97448 | CCT | 0 | 0 | 0 | 0.017445 | 0 | 0 | 0 | 0 | 0 | 0 | 0 | 0 | 0 | 0 | 0 | 0 | 0 | 0.015393 | 0 | 0 | 0 | 0 | 0 | 0 | 0 | 0 | 0.060545 | 0 | 0 | 0 | 0 |
| 140992 | CAA | 0 | 0 | 0 | 0 | 0 | 0 | 0 | 0 | 0.026243 | 0 | 0 | 0 | 0 | 0 | 0 | 0 | 0.008511 | 0.003546 | 0 | 0 | 0 | 0.024115 | 0 | 0.002128 | 0 | 0 | 0 | 0 | 0 | 0 | 0 |
| 66314 | CAG | 0 | 0 | 0 | 0 | 0 | 0 | 0 | 0 | 0.006032 | 0.021112 | 0 | 0 | 0 | 0 | 0 | 0 | 0.006032 | 0.006032 | 0 | 0 | 0 | 0 | 0.021112 | 0 | 0 | 0.003016 | 0 | 0 | 0 | 0 | 0 |
| 86538 | AGA | 0 | 0.001156 | 0 | 0 | 0 | 0 | 0 | 0 | 0 | 0 | 0 | 0 | 0.091289 | 0 | 0.002311 | 0 | 0 | 0 | 0.013867 | 0 | 0 | 0.108623 | 0.001156 | 0 | 0 | 0 | 0 | 0 | 0 | 0 | 0 |
| 47416 | AGG | 0 | 0 | 0 | 0 | 0 | 0 | 0 | 0.004218 | 0 | 0 | 0 | 0 | 0.004218 | 0 | 0.094905 | 0 | 0 | 0 | 0 | 0 | 0 | 0 | 0.170828 | 0 | 0 | 0 | 0 | 0 | 0 | 0.023199 | 0 |
| 61874 | CGA | 0 | 0 | 0 | 0 | 0 | 0 | 0 | 0 | 0 | 0 | 0 | 0 | 0.025859 | 0 | 0 | 0 | 0 | 0 | 0 | 0 | 0 | 0 | 0 | 0.004849 | 0 | 0 | 0 | 0 | 0 | 0 | 0 |
| 38690 | CGC | 0 | 0 | 0 | 0 | 0.064616 | 0.005169 | 0 | 0 | 0 | 0 | 0 | 0 | 0 | 0.005169 | 0 | 0 | 0.121478 | 0 | 0 | 0 | 0 | 0 | 0 | 0 | 0.007754 | 0 | 0 | 0 | 0.005169 | 0 | 0 |
| 21087 | CGG | 0 | 0 | 0 | 0 | 0 | 0 | 0 | 0 | 0 | 0 | 0 | 0 | 0 | 0 | 0.037938 | 0 | 0 | 0 | 0 | 0 | 0 | 0 | 0 | 0 | 0 | 0.037938 | 0 | 0 | 0 | 0 | 0 |
| 50170 | CGT | 0 | 0 | 0 | 0 | 0 | 0.065776 | 0 | 0 | 0 | 0 | 0 | 0 | 0 | 0.001993 | 0 | 0.003986 | 0 | 0.125573 | 0 | 0 | 0 | 0 | 0 | 0 | 0 | 0 | 0.027905 | 0 | 0 | 0 | 0 |
| 56399 | AGC | 0 | 0 | 0 | 0 | 0.007092 | 0 | 0 | 0 | 0 | 0 | 0 | 0 | 0 | 0.140073 | 0 | 0.007092 | 0 | 0 | 0 | 0.017731 | 0 | 0 | 0.003546 | 0 | 0 | 0 | 0 | 0 | 0 | 0 | 0.102839 |
| 83837 | AGT | 0 | 0 | 0 | 0 | 0 | 0.00835 | 0 | 0 | 0 | 0 | 0 | 0 | 0 | 0.001193 | 0 | 0.12405 | 0 | 0 | 0 | 0 | 0.023856 | 0 | 0 | 0 | 0 | 0 | 0 | 0 | 0 | 0 | 0 |
| 95815 | TCA | 0.018786 | 0 | 0.003131 | 0 | 0 | 0 | 0 | 0 | 0 | 0 | 0 | 0 | 0 | 0 | 0 | 0 | 0 | 0 | 0 | 0 | 0.001044 | 0 | 0 | 0 | 0 | 0 | 0 | 0.057402 | 0 | 0 | 0 |
| 73646 | TCC | 0 | 0.010863 | 0.004074 | 0 | 0.005431 | 0 | 0 | 0.001358 | 0 | 0 | 0.086902 | 0 | 0 | 0 | 0 | 0 | 0 | 0 | 0 | 0 | 0 | 0 | 0 | 0 | 0 | 0 | 0 | 0 | 0 | 0 | 0 |
| 55015 | TCG | 0 | 0 | 0.009088 | 0 | 0 | 0 | 0 | 0 | 0 | 0 | 0 | 0 | 0 | 0 | 0 | 0 | 0 | 0 | 0 | 0 | 0 | 0 | 0 | 0 | 0 | 0 | 0 | 0 | 0.10179 | 0 | 0 |
| 100452 | TCT | 0.001991 | 0 | 0 | 0.017919 | 0 | 0.007964 | 0 | 0 | 0 | 0 | 0 | 0.033847 | 0 | 0 | 0 | 0 | 0 | 0 | 0 | 0 | 0 | 0 | 0 | 0.001991 | 0 | 0.004978 | 0 | 0 | 0 | 0 | 0 |
| 100810 | ACA | 0.109116 | 0 | 0.000992 | 0 | 0 | 0 | 0 | 0 | 0 | 0 | 0 | 0 | 0 | 0 | 0 | 0 | 0.00992 | 0 | 0.112092 | 0 | 0 | 0.033727 | 0 | 0 | 0 | 0 | 0 | 0 | 0 | 0 | 0 |
| 78855 | ACC | 0 | 0.083698 | 0 | 0 | 0 | 0 | 0 | 0 | 0 | 0 | 0 | 0 | 0 | 0 | 0 | 0 | 0 | 0 | 0 | 0.101452 | 0 | 0 | 0 | 0 | 0 | 0 | 0 | 0 | 0 | 0 | 0.011413 |
| 51302 | ACG | 0 | 0 | 0.116955 | 0 | 0 | 0 | 0 | 0 | 0 | 0 | 0 | 0 | 0 | 0 | 0 | 0 | 0 | 0 | 0 | 0 | 0 | 0 | 0.021442 | 0 | 0 | 0 | 0 | 0 | 0 | 0.124751 | 0 |
| 98119 | ACT | 0.001019 | 0 | 0 | 0.112109 | 0 | 0 | 0 | 0.001019 | 0 | 0 | 0 | 0 | 0.003058 | 0 | 0 | 0 | 0 | 0 | 0 | 0 | 0.076438 | 0 | 0 | 0 | 0 | 0 | 0 | 0 | 0 | 0 | 0 |
| 59258 | GTA | 0.133315 | 0 | 0 | 0 | 0 | 0 | 0 | 0 | 0.008438 | 0 | 0 | 0 | 0.016875 | 0 | 0 | 0 | 0 | 0 | 0.151878 | 0 | 0 | 0 | 0 | 0.011813 | 0 | 0 | 0 | 0.050626 | 0.001688 | 0 | 0 |
| 76783 | GTC | 0 | 0.088561 | 0 | 0 | 0 | 0 | 0.005209 | 0 | 0 | 0 | 0.026047 | 0 | 0 | 0.003907 | 0 | 0 | 0 | 0 | 0 | 0.130237 | 0.005209 | 0 | 0 | 0 | 0.015628 | 0 | 0 | 0.002605 | 0 | 0 | 0 |
| 57638 | GTG | 0 | 0 | 0.144002 | 0 | 0 | 0 | 0 | 0 | 0 | 0.00347 | 0 | 0 | 0 | 0 | 0.019085 | 0 | 0 | 0 | 0.00694 | 0.001735 | 0.001735 | 0 | 0 | 0 | 0 | 0.015615 | 0 | 0 | 0.019085 | 0.150942 | 0 |
| 108970 | GTT | 0 | 0 | 0 | 0.085345 | 0 | 0 | 0 | 0.003671 | 0 | 0 | 0 | 0.02386 | 0 | 0 | 0 | 0.017436 | 0 | 0 | 0.000918 | 0.001835 | 0.158759 | 0 | 0 | 0 | 0 | 0 | 0.018354 | 0 | 0.001835 | 0 | 0 |
| 73339 | TGG | 0 | 0 | 0 | 0 | 0.005454 | 0.006818 | 0 | 0 | 0 | 0 | 0 | 0.002727 | 0.001364 | 0 | 0.009545 | 0 | 0 | 0 | 0 | 0 | 0 | 0 | 0 | 0 | 0 | 0.001364 | 0 | 0 | 0.010908 | 0 | 0 |
| 63486 | TAC | 0 | 0 | 0 | 0 | 0.026778 | 0 | 0.007876 | 0 | 0.001575 | 0 | 0.004725 | 0 | 0 | 0 | 0 | 0 | 0.053555 | 0 | 0 | 0 | 0 | 0 | 0 | 0 | 0 | 0 | 0 | 0 | 0 | 0 | 0.004725 |
| 86449 | TAT | 0 | 0 | 0 | 0 | 0 | 0.056681 | 0 | 0.028919 | 0 | 0 | 0.002314 | 0.016195 | 0 | 0 | 0 | 0 | 0 | 0.077502 | 0.001157 | 0 | 0 | 0 | 0 | 0 | 0 | 0 | 0 | 0 | 0 | 0 | 0 |
| 3522 | TAA | 0 | 0 | 0 | 0 | 0 | 0 | 0 | 0 | 0.085179 | 0 | 0 | 0 | 0 | 0 | 0 | 0 | 0 | 0 | 0 | 0 | 0 | 0.056786 | 0 | 0 | 0 | 0 | 0 | 0.028393 | 0 | 0 | 0 |
| 3373 | TAG | 0 | 0 | 0 | 0 | 0 | 0 | 0 | 0 | 0 | 0.088942 | 0 | 0 | 0 | 0 | 0 | 0 | 0 | 0 | 0 | 0 | 0 | 0 | 0.118589 | 0 | 0 | 0 | 0 | 0 | 0 | 0 | 0 |
| 4235 | TGA | 0 | 0 | 0 | 0 | 0.047226 | 0 | 0 | 0 | 0 | 0 | 0 | 0 | 0 | 0 | 0 | 0 | 0 | 0 | 0 | 0 | 0 | 0 | 0 | 0 | 0 | 0 | 0 | 0.047226 | 0 | 0 | 0 |

|  |  | **Var.** |  |  |  |  |  |  |  |  |  |  |  |  |  |  |  |  |  |  |  |  |  |  |  |  |  |  |  |  |  |  |  |  |
| --- | --- | --- | --- | --- | --- | --- | --- | --- | --- | --- | --- | --- | --- | --- | --- | --- | --- | --- | --- | --- | --- | --- | --- | --- | --- | --- | --- | --- | --- | --- | --- | --- | --- | --- |
| Ref. |  | **AAT** | **CCA** | **CCC** | **CCG** | **CCT** | **CAA** | **CAG** | **AGA** | **AGG** | **CGA** | **CGC** | **CGG** | **CGT** | **AGC** | **AGT** | **TCA** | **TCC** | **TCG** | **TCT** | **ACA** | **ACC** | **ACG** | **ACT** | **GTA** | **GTC** | **GTG** | **GTT** | **TGG** | **TAC** | **TAT** | **TAA** | **TAG** | TGA |
| 125745 | GCA | 0 | 0.007157 | 0 | 0 | 0 | 0 | 0 | 0 | 0 | 0 | 0 | 0 | 0 | 0 | 0 | 0.019882 | 0 | 0 | 0 | 0.097817 | 0.000795 | 0 | 0 | 0.066802 | 0 | 0 | 0 | 0 | 0 | 0 | 0 | 0 | 0 |
| 84025 | GCC | 0 | 0 | 0.008331 | 0 | 0 | 0.00238 | 0 | 0.00119 | 0 | 0 | 0 | 0 | 0 | 0 | 0 | 0 | 0.015472 | 0 | 0 | 0 | 0.107111 | 0 | 0.00238 | 0 | 0.086879 | 0 | 0 | 0 | 0 | 0 | 0 | 0 | 0 |
| 61223 | GCG | 0 | 0 | 0 | 0.011434 | 0 | 0 | 0 | 0 | 0 | 0 | 0 | 0 | 0 | 0.001633 | 0 | 0 | 0 | 0.029401 | 0 | 0 | 0 | 0.109436 | 0 | 0 | 0 | 0.109436 | 0 | 0 | 0 | 0 | 0 | 0 | 0 |
| 137091 | GCT | 0 | 0 | 0 | 0 | 0.004377 | 0 | 0 | 0 | 0 | 0 | 0 | 0 | 0 | 0 | 0 | 0 | 0 | 0 | 0.013859 | 0 | 0.000729 | 0 | 0.09191 | 0 | 0 | 0 | 0.072215 | 0 | 0 | 0 | 0 | 0 | 0 |
| 26836 | TGC | 0 | 0 | 0 | 0 | 0 | 0 | 0 | 0 | 0 | 0 | 0.119243 | 0 | 0 | 0 | 0.011179 | 0 | 0.011179 | 0 | 0 | 0 | 0 | 0 | 0 | 0 | 0 | 0 | 0 | 0.007453 | 0.085706 | 0 | 0 | 0 | 0 |
| 36133 | TGT | 0 | 0 | 0 | 0 | 0 | 0 | 0 | 0 | 0 | 0 | 0 | 0 | 0.083027 | 0 | 0.02214 | 0 | 0 | 0 | 0.008303 | 0 | 0 | 0 | 0 | 0 | 0 | 0 | 0 | 0.024908 | 0 | 0.127307 | 0 | 0 | 0 |
| 84761 | GAC | 0.00236 | 0 | 0 | 0 | 0 | 0 | 0.00118 | 0 | 0 | 0 | 0 | 0 | 0 | 0 | 0 | 0 | 0 | 0 | 0 | 0.003539 | 0 | 0 | 0 | 0 | 0.00236 | 0 | 0 | 0 | 0.011798 | 0 | 0 | 0 | 0 |
| 210269 | GAT | 0.074666 | 0.000476 | 0 | 0 | 0 | 0 | 0 | 0 | 0 | 0 | 0 | 0 | 0 | 0 | 0 | 0 | 0 | 0 | 0 | 0.000951 | 0 | 0.000476 | 0 | 0 | 0 | 0 | 0.003329 | 0 | 0 | 0.013792 | 0 | 0 | 0 |
| 203181 | GAA | 0.001969 | 0 | 0 | 0 | 0 | 0.010828 | 0 | 0.000492 | 0 | 0 | 0 | 0 | 0 | 0 | 0 | 0 | 0 | 0 | 0 | 0 | 0 | 0 | 0.000492 | 0.002953 | 0 | 0 | 0 | 0 | 0 | 0 | 0.005906 | 0 | 0 |
| 148627 | GAG | 0 | 0 | 0 | 0 | 0 | 0 | 0.012784 | 0 | 0.002691 | 0 | 0 | 0 | 0 | 0 | 0 | 0 | 0 | 0 | 0 | 0 | 0 | 0 | 0 | 0 | 0 | 0.004037 | 0 | 0 | 0 | 0 | 0 | 0.004037 | 0 |
| 100976 | TTC | 0 | 0 | 0 | 0 | 0.001981 | 0 | 0 | 0 | 0 | 0 | 0 | 0 | 0 | 0 | 0 | 0 | 0.044565 | 0 | 0.00099 | 0 | 0 | 0 | 0 | 0 | 0.021787 | 0 | 0.001981 | 0 | 0.006932 | 0 | 0 | 0 | 0 |
| 96924 | TTT | 0 | 0 | 0 | 0 | 0 | 0 | 0 | 0 | 0 | 0 | 0 | 0 | 0 | 0 | 0 | 0 | 0 | 0 | 0.040238 | 0 | 0 | 0 | 0 | 0 | 0 | 0 | 0.027857 | 0 | 0 | 0.018571 | 0 | 0 | 0 |
| 125365 | GGA | 0 | 0 | 0 | 0 | 0 | 0 | 0 | 0.069397 | 0 | 0.011167 | 0 | 0 | 0 | 0 | 0 | 0 | 0 | 0 | 0 | 0 | 0 | 0 | 0 | 0.006381 | 0 | 0 | 0 | 0 | 0 | 0 | 0 | 0 | 0 |
| 66322 | GGC | 0 | 0 | 0 | 0 | 0 | 0 | 0 | 0.003016 | 0 | 0 | 0.007539 | 0 | 0 | 0.105546 | 0.003016 | 0 | 0 | 0 | 0 | 0 | 0 | 0 | 0 | 0 | 0.004523 | 0 | 0 | 0.001508 | 0 | 0 | 0 | 0 | 0 |
| 51385 | GGG | 0 | 0 | 0 | 0 | 0 | 0 | 0 | 0.00973 | 0.091466 | 0 | 0 | 0.017515 | 0 | 0 | 0 | 0 | 0 | 0 | 0 | 0 | 0 | 0 | 0 | 0 | 0 | 0.005838 | 0 | 0.015569 | 0 | 0 | 0 | 0 | 0 |
| 116868 | GGT | 0 | 0 | 0 | 0 | 0 | 0 | 0 | 0.000856 | 0 | 0 | 0 | 0 | 0.003423 | 0.000856 | 0.079577 | 0 | 0 | 0 | 0 | 0 | 0 | 0 | 0 | 0 | 0 | 0.000856 | 0.012835 | 0 | 0.001711 | 0 | 0 | 0 | 0 |
| 43371 | CAC | 0.002306 | 0 | 0.004611 | 0 | 0 | 0.034585 | 0.027668 | 0 | 0 | 0 | 0.092228 | 0 | 0 | 0 | 0 | 0 | 0 | 0 | 0 | 0 | 0 | 0 | 0 | 0 | 0 | 0 | 0 | 0 | 0.138341 | 0.002306 | 0 | 0 | 0 |
| 78541 | CAT | 0.021645 | 0 | 0 | 0 | 0.025464 | 0.015279 | 0.014005 | 0 | 0 | 0 | 0 | 0 | 0.053475 | 0 | 0 | 0 | 0 | 0 | 0 | 0 | 0 | 0 | 0 | 0 | 0 | 0 | 0 | 0 | 0 | 0.105677 | 0 | 0 | 0 |
| 62765 | ATA | 0 | 0 | 0 | 0 | 0 | 0 | 0 | 0.020712 | 0 | 0 | 0 | 0 | 0 | 0 | 0 | 0 | 0 | 0 | 0 | 0.164104 | 0 | 0 | 0 | 0.144985 | 0 | 0.006373 | 0 | 0 | 0 | 0 | 0 | 0 | 0 |
| 105349 | ATC | 0 | 0 | 0 | 0 | 0 | 0 | 0 | 0 | 0 | 0 | 0 | 0 | 0 | 0.000949 | 0 | 0 | 0 | 0 | 0 | 0 | 0.070243 | 0 | 0 | 0 | 0.107263 | 0 | 0.000949 | 0 | 0 | 0 | 0 | 0 | 0 |
| 128014 | ATT | 0.008593 | 0 | 0.000781 | 0 | 0 | 0 | 0 | 0 | 0 | 0 | 0 | 0 | 0 | 0 | 0.014061 | 0 | 0 | 0 | 0 | 0 | 0 | 0 | 0.057025 | 0 | 0 | 0 | 0.128111 | 0 | 0 | 0 | 0 | 0 | 0 |
| 151418 | AAA | 0.013869 | 0 | 0 | 0 | 0 | 0.0317 | 0 | 0.058778 | 0 | 0 | 0 | 0 | 0 | 0 | 0 | 0 | 0 | 0 | 0 | 0.017171 | 0 | 0 | 0 | 0 | 0 | 0 | 0 | 0 | 0 | 0 | 0.001981 | 0 | 0 |
| 144104 | AAG | 0.022206 | 0 | 0 | 0 | 0 | 0 | 0.004858 | 0.002082 | 0.053434 | 0 | 0 | 0 | 0 | 0 | 0 | 0 | 0 | 0 | 0 | 0 | 0 | 0.015267 | 0 | 0 | 0 | 0 | 0 | 0 | 0 | 0 | 0 | 0.000694 | 0 |
| 50733 | CTA | 0 | 0.151775 | 0.001971 | 0 | 0 | 0.011827 | 0 | 0 | 0 | 0.019711 | 0 | 0 | 0 | 0 | 0 | 0 | 0 | 0 | 0 | 0 | 0 | 0 | 0 | 0.019711 | 0 | 0 | 0 | 0 | 0 | 0 | 0 | 0 | 0 |
| 89730 | CTC | 0 | 0 | 0.032319 | 0 | 0 | 0 | 0 | 0 | 0 | 0 | 0.003343 | 0 | 0 | 0 | 0 | 0 | 0 | 0 | 0 | 0 | 0 | 0 | 0 | 0 | 0.004458 | 0 | 0 | 0 | 0 | 0.001114 | 0 | 0 | 0 |
| 40617 | CTG | 0.002462 | 0 | 0 | 0.100943 | 0 | 0 | 0.009848 | 0 | 0 | 0 | 0 | 0.017234 | 0 | 0 | 0 | 0 | 0 | 0 | 0 | 0 | 0 | 0 | 0 | 0 | 0 | 0.014772 | 0 | 0 | 0 | 0 | 0 | 0 | 0 |
| 102767 | CTT | 0 | 0 | 0 | 0 | 0.054492 | 0 | 0 | 0 | 0 | 0 | 0 | 0 | 0.010704 | 0 | 0 | 0 | 0 | 0 | 0.000973 | 0 | 0 | 0 | 0 | 0 | 0 | 0 | 0.010704 | 0.000973 | 0 | 0 | 0 | 0 | 0 |
| 70944 | TTA | 0 | 0 | 0.002819 | 0 | 0.00141 | 0 | 0 | 0 | 0 | 0 | 0 | 0 | 0 | 0 | 0 | 0.074707 | 0 | 0 | 0 | 0 | 0 | 0 | 0 | 0.025372 | 0 | 0 | 0 | 0 | 0 | 0 | 0.004229 | 0 | 0.00141 |
| 100106 | TTG | 0 | 0 | 0 | 0 | 0 | 0.001998 | 0 | 0 | 0 | 0 | 0 | 0 | 0 | 0 | 0 | 0 | 0 | 0.047949 | 0 | 0 | 0 | 0 | 0 | 0 | 0 | 0.005994 | 0 | 0.001998 | 0 | 0 | 0.003996 | 0.003996 | 0 |
| 117646 | ATG | 0 | 0 | 0 | 0 | 0 | 0 | 0 | 0 | 0.0051 | 0 | 0 | 0 | 0 | 0 | 0 | 0 | 0 | 0 | 0 | 0 | 0 | 0.062901 | 0 | 0.00085 | 0 | 0.0527 | 0.0034 | 0 | 0 | 0 | 0 | 0 | 0 |
| 85470 | AAC | 0.234 | 0 | 0 | 0 | 0 | 0 | 0 | 0 | 0 | 0 | 0 | 0 | 0 | 0.07371 | 0 | 0 | 0 | 0 | 0 | 0 | 0.01287 | 0 | 0.00234 | 0 | 0.00117 | 0 | 0 | 0 | 0 | 0 | 0 | 0 | 0 |
| 142377 | AAT | 0 | 0 | 0 | 0 | 0.000702 | 0 | 0 | 0 | 0 | 0 | 0 | 0 | 0 | 0.000702 | 0.061808 | 0 | 0 | 0 | 0 | 0 | 0 | 0 | 0.013345 | 0 | 0.000702 | 0 | 0 | 0 | 0 | 0.004917 | 0.004214 | 0 | 0 |
| 115642 | CCA | 0 | 0 | 0.010377 | 0.104633 | 0.01643 | 0.014701 | 0 | 0 | 0 | 0.004324 | 0 | 0 | 0 | 0 | 0 | 0.10031 | 0.001729 | 0 | 0.000865 | 0.019889 | 0 | 0 | 0 | 0 | 0 | 0 | 0 | 0 | 0 | 0 | 0.000865 | 0 | 0 |
| 53741 | CCC | 0 | 0.031633 | 0 | 0.022329 | 0.24004 | 0 | 0 | 0 | 0 | 0 | 0.005582 | 0 | 0 | 0 | 0 | 0.001861 | 0.09676 | 0 | 0.005582 | 0.001861 | 0.016747 | 0 | 0 | 0 | 0 | 0 | 0 | 0 | 0 | 0 | 0 | 0 | 0 |
| 41063 | CCG | 0 | 0.284928 | 0.029223 | 0 | 0.060882 | 0 | 0.014612 | 0 | 0 | 0 | 0 | 0.007306 | 0.009741 | 0 | 0 | 0.002435 | 0.002435 | 0.0828 | 0 | 0 | 0 | 0.012176 | 0 | 0 | 0 | 0 | 0 | 0 | 0 | 0 | 0 | 0 | 0 |
| 97448 | CCT | 0 | 0.009236 | 0.132378 | 0.014367 | 0 | 0 | 0 | 0 | 0 | 0 | 0.001026 | 0 | 0.004105 | 0 | 0 | 0 | 0 | 0 | 0.079017 | 0.001026 | 0 | 0 | 0.019498 | 0 | 0 | 0 | 0 | 0 | 0 | 0 | 0 | 0 | 0 |
| 140992 | CAA | 0 | 0.005674 | 0 | 0 | 0 | 0 | 0.079437 | 0 | 0 | 0.043974 | 0 | 0 | 0 | 0 | 0 | 0 | 0 | 0 | 0 | 0 | 0 | 0 | 0 | 0 | 0 | 0 | 0 | 0 | 0 | 0 | 0.017732 | 0 | 0 |
| 66314 | CAG | 0 | 0 | 0 | 0.006032 | 0 | 0.174925 | 0 | 0 | 0 | 0 | 0 | 0.075399 | 0 | 0 | 0 | 0 | 0 | 0 | 0 | 0 | 0 | 0 | 0 | 0 | 0 | 0 | 0 | 0 | 0 | 0.001508 | 0 | 0.019604 | 0 |
| 86538 | AGA | 0 | 0 | 0 | 0 | 0 | 0 | 0 | 0 | 0.095912 | 0.018489 | 0 | 0 | 0 | 0.006933 | 0.005778 | 0 | 0 | 0 | 0 | 0.013867 | 0 | 0 | 0 | 0 | 0 | 0 | 0 | 0 | 0 | 0 | 0 | 0 | 0 |
| 47416 | AGG | 0 | 0 | 0 | 0 | 0 | 0 | 0 | 0.215117 | 0 | 0.002109 | 0 | 0.014763 | 0 | 0.02109 | 0.004218 | 0 | 0 | 0 | 0 | 0 | 0 | 0.037962 | 0 | 0 | 0 | 0 | 0 | 0.004218 | 0 | 0 | 0 | 0 | 0 |
| 61874 | CGA | 0 | 0.009697 | 0 | 0.001616 | 0 | 0.074345 | 0 | 0.037172 | 0 | 0 | 0.006465 | 0.072728 | 0.009697 | 0 | 0 | 0 | 0 | 0 | 0 | 0 | 0 | 0 | 0 | 0 | 0 | 0 | 0 | 0 | 0 | 0 | 0 | 0 | 0.00162 |
| 38690 | CGC | 0 | 0 | 0.007754 | 0 | 0 | 0 | 0 | 0 | 0 | 0.010339 | 0 | 0.007754 | 0.165417 | 0.012923 | 0 | 0 | 0 | 0 | 0 | 0 | 0 | 0 | 0 | 0 | 0 | 0 | 0 | 0 | 0 | 0 | 0 | 0 | 0 |
| 21087 | CGG | 0 | 0 | 0 | 0.037938 | 0 | 0 | 0.180206 | 0 | 0.023711 | 0.227628 | 0.028454 | 0 | 0.028454 | 0 | 0 | 0 | 0 | 0 | 0 | 0 | 0 | 0 | 0 | 0 | 0 | 0 | 0 | 0.109072 | 0 | 0 | 0 | 0 | 0 |
| 50170 | CGT | 0 | 0 | 0 | 0 | 0.00598 | 0 | 0 | 0 | 0 | 0.003986 | 0.095675 | 0.009966 | 0 | 0 | 0.021925 | 0 | 0 | 0 | 0 | 0 | 0 | 0 | 0 | 0 | 0 | 0 | 0 | 0 | 0 | 0.011959 | 0 | 0 | 0 |
| 56399 | AGC | 0 | 0 | 0 | 0 | 0 | 0 | 0 | 0.003546 | 0.003546 | 0 | 0.001773 | 0 | 0 | 0 | 0.177308 | 0 | 0 | 0 | 0.001773 | 0 | 0.014185 | 0 | 0 | 0 | 0 | 0 | 0 | 0 | 0 | 0 | 0 | 0 | 0 |
| 83837 | AGT | 0.121665 | 0 | 0 | 0 | 0 | 0 | 0 | 0.005964 | 0.00835 | 0 | 0 | 0 | 0.00835 | 0.115701 | 0 | 0 | 0 | 0 | 0 | 0 | 0 | 0 | 0.027434 | 0 | 0.001193 | 0 | 0 | 0 | 0 | 0 | 0 | 0 | 0 |
| 95815 | TCA | 0 | 0.105411 | 0 | 0 | 0 | 0 | 0.001044 | 0 | 0 | 0 | 0 | 0 | 0 | 0 | 0 | 0 | 0.020874 | 0.12211 | 0.017743 | 0.006262 | 0.002087 | 0 | 0 | 0 | 0 | 0 | 0 | 0 | 0 | 0 | 0.002087 | 0 | 0.00104 |
| 73646 | TCC | 0 | 0.008147 | 0.078755 | 0 | 0.004074 | 0.001358 | 0 | 0 | 0 | 0 | 0 | 0 | 0 | 0 | 0 | 0.033946 | 0 | 0.013578 | 0.186025 | 0 | 0.010863 | 0 | 0 | 0 | 0 | 0 | 0 | 0 | 0.006789 | 0 | 0.001358 | 0 | 0 |
| 55015 | TCG | 0 | 0 | 0 | 0.052713 | 0 | 0 | 0 | 0 | 0 | 0 | 0 | 0 | 0 | 0 | 0 | 0.210852 | 0.036354 | 0 | 0.030901 | 0 | 0 | 0.018177 | 0 | 0 | 0 | 0 | 0 | 0.003635 | 0 | 0 | 0 | 0 | 0 |
| 100452 | TCT | 0 | 0 | 0 | 0 | 0.101541 | 0 | 0 | 0 | 0 | 0 | 0 | 0 | 0 | 0 | 0 | 0.00896 | 0.155298 | 0.013937 | 0 | 0 | 0.000996 | 0 | 0.010951 | 0 | 0 | 0 | 0 | 0 | 0 | 0.018915 | 0 | 0 | 0 |
| 100810 | ACA | 0 | 0.007936 | 0 | 0 | 0 | 0 | 0 | 0.007936 | 0 | 0 | 0 | 0 | 0 | 0 | 0 | 0.019839 | 0 | 0 | 0 | 0 | 0.027775 | 0.122012 | 0.018847 | 0 | 0 | 0 | 0 | 0 | 0.006944 | 0 | 0 | 0 | 0 |
| 78855 | ACC | 0 | 0 | 0.015218 | 0 | 0 | 0 | 0 | 0 | 0 | 0 | 0 | 0 | 0 | 0.01395 | 0 | 0 | 0.001268 | 0 | 0 | 0.041849 | 0 | 0.02029 | 0.216854 | 0 | 0 | 0 | 0 | 0 | 0 | 0 | 0 | 0 | 0 |
| 51302 | ACG | 0 | 0 | 0 | 0.003898 | 0 | 0 | 0 | 0 | 0.013645 | 0 | 0 | 0.001949 | 0 | 0 | 0 | 0 | 0 | 0.009746 | 0 | 0.274843 | 0.029239 | 0 | 0.046782 | 0 | 0 | 0 | 0 | 0 | 0 | 0 | 0 | 0 | 0 |
| 98119 | ACT | 0.031594 | 0 | 0 | 0 | 0.021403 | 0 | 0 | 0 | 0 | 0 | 0 | 0 | 0 | 0 | 0.026498 | 0 | 0 | 0 | 0.011211 | 0.010192 | 0.16001 | 0.014268 | 0 | 0 | 0 | 0 | 0 | 0 | 0 | 0 | 0 | 0 | 0 |
| 59258 | GTA | 0 | 0 | 0 | 0 | 0 | 0 | 0 | 0 | 0 | 0 | 0 | 0 | 0 | 0 | 0 | 0 | 0 | 0 | 0 | 0 | 0 | 0 | 0 | 0 | 0.030376 | 0.150191 | 0.028688 | 0 | 0 | 0 | 0 | 0 | 0 |
| 76783 | GTC | 0 | 0 | 0 | 0 | 0 | 0 | 0 | 0 | 0 | 0 | 0 | 0.001302 | 0 | 0 | 0 | 0 | 0 | 0 | 0 | 0 | 0 | 0 | 0 | 0.014326 | 0 | 0.024745 | 0.158889 | 0 | 0 | 0 | 0 | 0 | 0 |
| 57638 | GTG | 0 | 0 | 0 | 0 | 0 | 0 | 0 | 0 | 0 | 0 | 0 | 0 | 0 | 0 | 0 | 0 | 0 | 0 | 0 | 0 | 0 | 0 | 0 | 0.159617 | 0.02429 | 0 | 0.034699 | 0 | 0 | 0 | 0 | 0.00694 | 0 |
| 108970 | GTT | 0.000918 | 0 | 0 | 0 | 0.000918 | 0 | 0 | 0 | 0 | 0 | 0 | 0 | 0 | 0 | 0.000918 | 0 | 0 | 0 | 0 | 0 | 0.000918 | 0 | 0 | 0.010095 | 0.110122 | 0.013765 | 0 | 0 | 0 | 0 | 0 | 0 | 0 |
| 73339 | TGG | 0 | 0 | 0 | 0 | 0 | 0 | 0 | 0 | 0 | 0 | 0.002727 | 0.039542 | 0 | 0 | 0 | 0 | 0 | 0.009545 | 0 | 0 | 0 | 0 | 0 | 0 | 0 | 0 | 0 | 0 | 0 | 0 | 0.001364 | 0.051814 | 0.00273 |
| 63486 | TAC | 0 | 0 | 0 | 0 | 0 | 0 | 0 | 0 | 0 | 0 | 0 | 0 | 0 | 0 | 0 | 0 | 0.006301 | 0 | 0 | 0 | 0 | 0 | 0 | 0 | 0 | 0 | 0 | 0 | 0 | 0.201619 | 0.006301 | 0.00315 | 0 |
| 86449 | TAT | 0.011568 | 0 | 0 | 0 | 0 | 0 | 0 | 0 | 0 | 0 | 0 | 0 | 0 | 0 | 0 | 0 | 0 | 0 | 0.008097 | 0 | 0 | 0 | 0 | 0 | 0 | 0.001157 | 0 | 0 | 0.153848 | 0 | 0.00347 | 0.001157 | 0 |
| 3522 | TAA | 0 | 0 | 0 | 0 | 0 | 0.312323 | 0 | 0 | 0 | 0 | 0 | 0 | 0 | 0 | 0 | 0 | 0 | 0 | 0 | 0 | 0 | 0 | 0 | 0 | 0 | 0 | 0 | 0 | 0.028393 | 0 | 0 | 0.141965 | 0.25554 |
| 3373 | TAG | 0 | 0 | 0 | 0 | 0 | 0 | 0.20753 | 0 | 0 | 0 | 0 | 0 | 0 | 0 | 0 | 0 | 0 | 0.059294 | 0 | 0 | 0 | 0 | 0 | 0 | 0 | 0 | 0 | 0.266825 | 0.029647 | 0 | 0.088942 | 0 | 0 |
| 4235 | TGA | 0 | 0 | 0 | 0 | 0 | 0 | 0 | 0.047226 | 0 | 0.165289 | 0 | 0 | 0 | 0 | 0 | 0.094451 | 0 | 0 | 0 | 0 | 0 | 0 | 0 | 0 | 0 | 0 | 0 | 0.118064 | 0 | 0 | 0.141677 | 0 | 0 |

Frequency of codon substitutions in *Z. tritici* population (as % of all codons). Ref. indicates codons in the reference genome (isolate IPO323) together with their total number, Var. indicates variant codons.

|  |  | **Var.** |  |  |  |  |  |  |  |  |  |  |  |  |  |  |  |  |  |  |  |  |  |  |  |  |  |  |  |  |  |  |
| --- | --- | --- | --- | --- | --- | --- | --- | --- | --- | --- | --- | --- | --- | --- | --- | --- | --- | --- | --- | --- | --- | --- | --- | --- | --- | --- | --- | --- | --- | --- | --- | --- |
| Ref. |  | **GCA** | **GCC** | **GCG** | **GCT** | **TGC** | **TGT** | **GAC** | **GAT** | **GAA** | **GAG** | **TTC** | **TTT** | **GGA** | **GGC** | **GGG** | **GGT** | **CAC** | **CAT** | **ATA** | **ATC** | **ATT** | **AAA** | **AAG** | **CTA** | **CTC** | **CTG** | **CTT** | **TTA** | **TTG** | **ATG** | **AAC** |
| 91754 | GCA | 0 | 0.40979 | 1.81028 | 0.54494 | 0 | 0 | 0 | 0 | 0.05667 | 0 | 0 | 0 | 0.05558 | 0 | 0 | 0 | 0 | 0 | 0 | 0 | 0 | 0 | 0 | 0 | 0 | 0 | 0 | 0 | 0 | 0 | 0 |
| 129116 | GCC | 0.29199 | 0 | 0.33768 | 0.90694 | 0 | 0 | 0.04337 | 0 | 0 | 0 | 0 | 0 | 0 | 0.04724 | 0 | 0 | 0 | 0 | 0 | 0 | 0 | 0 | 0 | 0 | 0 | 0 | 0 | 0 | 0 | 0 | 0 |
| 114409 | GCG | 1.45093 | 0.34613 | 0 | 0.47811 | 0 | 0 | 0 | 0 | 0 | 0.14597 | 0 | 0 | 0 | 0 | 0.03758 | 0 | 0 | 0 | 0 | 0 | 0 | 0 | 0 | 0 | 0 | 0 | 0 | 0 | 0 | 0 | 0 |
| 95119 | GCT | 0.53197 | 1.03344 | 0.62343 | 0 | 0 | 0 | 0 | 0.12931 | 0 | 0 | 0 | 0 | 0 | 0 | 0 | 0.082 | 0 | 0 | 0 | 0 | 0 | 0 | 0 | 0 | 0 | 0 | 0 | 0 | 0 | 0 | 0 |
| 39785 | TGC | 0 | 0 | 0 | 0 | 0 | 1.72175 | 0 | 0 | 0 | 0 | 0.07038 | 0 | 0 | 0.06032 | 0 | 0 | 0 | 0 | 0 | 0 | 0 | 0 | 0 | 0 | 0 | 0 | 0 | 0 | 0 | 0 | 0 |
| 20431 | TGT | 0 | 0 | 0 | 0 | 3.16186 | 0 | 0 | 0 | 0 | 0 | 0 | 0.02937 | 0 | 0 | 0 | 0.10278 | 0 | 0 | 0 | 0 | 0 | 0 | 0 | 0 | 0 | 0 | 0 | 0 | 0 | 0 | 0 |
| 159365 | GAC | 0 | 0.23782 | 0 | 0 | 0 | 0 | 0 | 2.67123 | 0.0822 | 0.2259 | 0 | 0 | 0 | 0.12675 | 0 | 0 | 0.05459 | 0 | 0 | 0 | 0 | 0 | 0 | 0 | 0 | 0 | 0 | 0 | 0 | 0 | 0.1324 |
| 116027 | GAT | 0 | 0 | 0 | 0.11118 | 0 | 0 | 3.47936 | 0 | 0.06464 | 0.18789 | 0 | 0 | 0 | 0 | 0 | 0.13445 | 0 | 0.08791 | 0 | 0 | 0 | 0 | 0 | 0 | 0 | 0 | 0 | 0 | 0 | 0 | 0 |
| 109354 | GAA | 0.04298 | 0 | 0 | 0 | 0 | 0 | 0.14814 | 0.09328 | 0 | 1.05346 | 0 | 0 | 0.10516 | 0 | 0 | 0 | 0 | 0 | 0 | 0 | 0 | 0.07316 | 0 | 0 | 0 | 0 | 0 | 0 | 0 | 0 | 0 |
| 188595 | GAG | 0 | 0 | 0.11718 | 0 | 0 | 0 | 0.20573 | 0.14157 | 0.49312 | 0 | 0 | 0 | 0 | 0 | 0.04507 | 0 | 0 | 0 | 0 | 0 | 0 | 0 | 0.09385 | 0 | 0 | 0 | 0 | 0 | 0 | 0 | 0 |
| 127303 | TTC | 0 | 0 | 0 | 0 | 0.01728 | 0 | 0 | 0 | 0 | 0 | 0 | 0.60407 | 0 | 0 | 0 | 0 | 0 | 0 | 0 | 0.03064 | 0 | 0 | 0 | 0 | 0.08562 | 0 | 0 | 0.0432 | 0.11233 | 0 | 0 |
| 45082 | TTT | 0 | 0 | 0 | 0 | 0 | 0.01331 | 0 | 0 | 0 | 0 | 1.77011 | 0 | 0 | 0 | 0 | 0 | 0 | 0 | 0 | 0 | 0.02884 | 0 | 0 | 0 | 0 | 0 | 0.06433 | 0.00887 | 0.03993 | 0 | 0 |
| 87998 | GGA | 0.07841 | 0 | 0 | 0 | 0 | 0 | 0 | 0 | 0.10114 | 0 | 0 | 0 | 0 | 1.02843 | 0.61024 | 0.79888 | 0 | 0 | 0 | 0 | 0 | 0 | 0 | 0 | 0 | 0 | 0 | 0 | 0 | 0 | 0 |
| 130985 | GGC | 0 | 0.04504 | 0 | 0 | 0.01603 | 0 | 0.14124 | 0 | 0 | 0 | 0 | 0 | 0.70771 | 0 | 0.22064 | 2.27889 | 0 | 0 | 0 | 0 | 0 | 0 | 0 | 0 | 0 | 0 | 0 | 0 | 0 | 0 | 0 |
| 41918 | GGG | 0 | 0 | 0.06203 | 0 | 0 | 0 | 0 | 0 | 0 | 0.22663 | 0 | 0 | 1.22859 | 0.49859 | 0 | 0.40078 | 0 | 0 | 0 | 0 | 0 | 0 | 0 | 0 | 0 | 0 | 0 | 0 | 0 | 0 | 0 |
| 71843 | GGT | 0 | 0 | 0 | 0.0515 | 0 | 0.01531 | 0 | 0.17956 | 0 | 0 | 0 | 0 | 0.92006 | 3.68164 | 0.77252 | 0 | 0 | 0 | 0 | 0 | 0 | 0 | 0 | 0 | 0 | 0 | 0 | 0 | 0 | 0 | 0 |
| 70062 | CAC | 0 | 0 | 0 | 0 | 0 | 0 | 0.0942 | 0 | 0 | 0 | 0 | 0 | 0 | 0 | 0 | 0 | 0 | 2.0125 | 0 | 0 | 0 | 0 | 0 | 0 | 0.08992 | 0 | 0 | 0 | 0 | 0 | 0.05281 |
| 45883 | CAT | 0 | 0 | 0 | 0 | 0 | 0 | 0 | 0.1591 | 0 | 0 | 0 | 0 | 0 | 0 | 0 | 0 | 2.79406 | 0 | 0 | 0 | 0 | 0 | 0 | 0 | 0 | 0 | 0.03487 | 0 | 0 | 0 | 0 |
| 20898 | ATA | 0 | 0 | 0 | 0 | 0 | 0 | 0 | 0 | 0 | 0 | 0 | 0 | 0 | 0 | 0 | 0 | 0 | 0 | 0 | 2.09589 | 0.33975 | 0.05742 | 0 | 0.13877 | 0 | 0 | 0 | 0.03828 | 0 | 0.90918 | 0 |
| 139627 | ATC | 0 | 0 | 0 | 0 | 0 | 0 | 0 | 0 | 0 | 0 | 0.02149 | 0 | 0 | 0 | 0 | 0 | 0 | 0 | 0.36383 | 0 | 0.9332 | 0 | 0 | 0 | 0.11459 | 0 | 0 | 0 | 0 | 0.11746 | 0.02507 |
| 60895 | ATT | 0 | 0 | 0 | 0 | 0 | 0 | 0 | 0 | 0 | 0 | 0 | 0.02956 | 0 | 0 | 0 | 0 | 0 | 0 | 0.20527 | 2.19722 | 0 | 0 | 0 | 0 | 0 | 0 | 0.13466 | 0 | 0 | 0.10346 | 0 |
| 70629 | AAA | 0 | 0 | 0 | 0 | 0 | 0 | 0 | 0 | 0.15574 | 0 | 0 | 0 | 0 | 0 | 0 | 0 | 0 | 0 | 0.02124 | 0 | 0 | 0 | 0.82402 | 0 | 0 | 0 | 0 | 0 | 0 | 0 | 0.06796 |
| 161919 | AAG | 0 | 0 | 0 | 0 | 0 | 0 | 0 | 0 | 0 | 0.12043 | 0 | 0 | 0 | 0 | 0 | 0 | 0 | 0 | 0 | 0 | 0 | 0.35326 | 0 | 0 | 0 | 0 | 0 | 0 | 0 | 0.02223 | 0.12784 |
| 26878 | CTA | 0 | 0 | 0 | 0 | 0 | 0 | 0 | 0 | 0 | 0 | 0 | 0 | 0 | 0 | 0 | 0 | 0 | 0 | 0.10417 | 0 | 0 | 0 | 0 | 0 | 1.53285 | 4.02188 | 0.30136 | 0.42042 | 0 | 0 | 0 |
| 142305 | CTC | 0 | 0 | 0 | 0 | 0 | 0 | 0 | 0 | 0 | 0 | 0.08362 | 0 | 0 | 0 | 0 | 0 | 0.04849 | 0 | 0 | 0.11033 | 0 | 0 | 0 | 0.3106 | 0 | 0.70482 | 0.58817 | 0 | 0 | 0 | 0 |
| 101325 | CTG | 0 | 0 | 0 | 0 | 0 | 0 | 0 | 0 | 0 | 0 | 0 | 0 | 0 | 0 | 0 | 0 | 0 | 0 | 0 | 0 | 0 | 0 | 0 | 1.22477 | 0.97113 | 0 | 0.21811 | 0 | 0.79447 | 0.13027 | 0 |
| 55859 | CTT | 0 | 0 | 0 | 0 | 0 | 0 | 0 | 0 | 0 | 0 | 0 | 0.05729 | 0 | 0 | 0 | 0 | 0 | 0.02864 | 0 | 0 | 0.13427 | 0 | 0 | 0.18618 | 1.53243 | 0.42786 | 0 | 0 | 0 | 0 | 0 |
| 11387 | TTA | 0 | 0 | 0 | 0 | 0 | 0 | 0 | 0 | 0 | 0 | 0.43032 | 0.01756 | 0 | 0 | 0 | 0 | 0 | 0 | 0.11417 | 0 | 0 | 0 | 0 | 1.01871 | 0 | 0 | 0 | 0 | 5.52384 | 0 | 0 |
| 78788 | TTG | 0 | 0 | 0 | 0 | 0 | 0 | 0 | 0 | 0 | 0 | 0.14215 | 0.04823 | 0 | 0 | 0 | 0 | 0 | 0 | 0 | 0 | 0 | 0 | 0 | 0 | 0 | 0.97858 | 0 | 0.95319 | 0 | 0.06219 | 0 |
| 106678 | ATG | 0 | 0 | 0 | 0 | 0 | 0 | 0 | 0 | 0 | 0 | 0 | 0 | 0 | 0 | 0 | 0 | 0 | 0 | 0.27747 | 0.10968 | 0.03468 | 0 | 0.0225 | 0 | 0 | 0.10968 | 0 | 0 | 0.05249 | 0 | 0 |
| 107930 | AAC | 0 | 0 | 0 | 0 | 0 | 0 | 0.21032 | 0 | 0 | 0 | 0 | 0 | 0 | 0 | 0 | 0 | 0.04818 | 0 | 0 | 0.03521 | 0 | 0.03335 | 0.14732 | 0 | 0 | 0 | 0 | 0 | 0 | 0 | 0 |
| 61171 | AAT | 0 | 0 | 0 | 0 | 0 | 0 | 0 | 0.2681 | 0 | 0 | 0 | 0 | 0 | 0 | 0 | 0 | 0 | 0.06703 | 0 | 0 | 0.01962 | 0.04904 | 0.19781 | 0 | 0 | 0 | 0 | 0 | 0 | 0 | 4.64436 |
| 72641 | CCA | 0.06195 | 0 | 0 | 0 | 0 | 0 | 0 | 0 | 0 | 0 | 0 | 0 | 0 | 0 | 0 | 0 | 0 | 0 | 0 | 0 | 0 | 0 | 0 | 0.17896 | 0 | 0 | 0 | 0 | 0 | 0 | 0 |
| 63677 | CCC | 0 | 0.05496 | 0 | 0 | 0 | 0 | 0 | 0 | 0 | 0 | 0 | 0 | 0 | 0 | 0 | 0 | 0.03926 | 0 | 0 | 0 | 0 | 0 | 0 | 0 | 0.12406 | 0 | 0 | 0 | 0 | 0 | 0 |
| 78843 | CCG | 0 | 0 | 0.05961 | 0 | 0 | 0 | 0 | 0 | 0 | 0 | 0 | 0 | 0 | 0 | 0 | 0 | 0 | 0 | 0 | 0 | 0 | 0 | 0 | 0 | 0 | 0.11796 | 0 | 0 | 0 | 0 | 0 |
| 60141 | CCT | 0 | 0 | 0 | 0.06318 | 0 | 0 | 0 | 0 | 0 | 0 | 0 | 0 | 0 | 0 | 0 | 0 | 0 | 0.03159 | 0 | 0 | 0 | 0 | 0 | 0 | 0 | 0 | 0.04323 | 0 | 0 | 0 | 0 |
| 84681 | CAA | 0 | 0 | 0 | 0 | 0 | 0 | 0 | 0 | 0.16296 | 0 | 0 | 0 | 0 | 0 | 0 | 0 | 0.09211 | 0.04606 | 0 | 0 | 0 | 0.08975 | 0 | 0.01299 | 0 | 0 | 0 | 0 | 0 | 0 | 0 |
| 106039 | CAG | 0 | 0 | 0 | 0 | 0 | 0 | 0 | 0 | 0 | 0.13674 | 0 | 0 | 0 | 0 | 0 | 0 | 0.13391 | 0.08959 | 0 | 0 | 0 | 0 | 0.06507 | 0 | 0 | 0.11034 | 0 | 0 | 0 | 0 | 0 |
| 37686 | AGA | 0 | 0 | 0 | 0 | 0 | 0 | 0 | 0 | 0 | 0 | 0 | 0 | 0.12471 | 0 | 0 | 0 | 0 | 0 | 0.08757 | 0 | 0 | 0.1884 | 0 | 0 | 0 | 0 | 0 | 0 | 0 | 0 | 0 |
| 39861 | AGG | 0 | 0 | 0 | 0 | 0 | 0 | 0 | 0 | 0 | 0 | 0 | 0 | 0 | 0 | 0.07024 | 0 | 0 | 0 | 0 | 0 | 0 | 0 | 0.25087 | 0 | 0 | 0 | 0 | 0 | 0 | 0.05268 | 0 |
| 58510 | CGA | 0 | 0 | 0 | 0 | 0 | 0 | 0 | 0 | 0 | 0 | 0 | 0 | 0.07007 | 0 | 0 | 0 | 0 | 0 | 0 | 0 | 0 | 0 | 0 | 0.06666 | 0 | 0 | 0 | 0 | 0 | 0 | 0 |
| 80514 | CGC | 0 | 0 | 0 | 0 | 0.13165 | 0 | 0 | 0 | 0 | 0 | 0 | 0 | 0 | 0.02111 | 0 | 0 | 0.35894 | 0 | 0 | 0 | 0 | 0 | 0 | 0 | 0.08694 | 0 | 0 | 0 | 0 | 0 | 0 |
| 38584 | CGG | 0 | 0 | 0 | 0 | 0 | 0 | 0 | 0 | 0 | 0 | 0 | 0 | 0 | 0 | 0.02333 | 0 | 0 | 0 | 0 | 0 | 0 | 0 | 0 | 0 | 0 | 0.04406 | 0 | 0 | 0 | 0 | 0 |
| 38270 | CGT | 0 | 0 | 0 | 0 | 0 | 0.16723 | 0 | 0 | 0 | 0 | 0 | 0 | 0 | 0 | 0 | 0.02613 | 0 | 0.56964 | 0 | 0 | 0 | 0 | 0 | 0 | 0 | 0 | 0.05749 | 0 | 0 | 0 | 0 |
| 79886 | AGC | 0 | 0 | 0 | 0 | 0.06885 | 0 | 0 | 0 | 0 | 0 | 0 | 0 | 0 | 0.2078 | 0 | 0 | 0 | 0 | 0 | 0.09263 | 0 | 0 | 0 | 0 | 0 | 0 | 0 | 0 | 0 | 0 | 0.2103 |
| 43894 | AGT | 0 | 0 | 0 | 0 | 0 | 0.06835 | 0 | 0 | 0 | 0 | 0 | 0 | 0 | 0 | 0 | 0.18681 | 0 | 0 | 0 | 0 | 0.02962 | 0 | 0 | 0 | 0 | 0 | 0 | 0 | 0 | 0 | 0 |
| 44792 | TCA | 0.28353 | 0 | 0 | 0 | 0 | 0 | 0 | 0 | 0 | 0 | 0 | 0 | 0 | 0 | 0 | 0 | 0 | 0 | 0 | 0 | 0 | 0 | 0 | 0 | 0 | 0 | 0 | 0.18084 | 0 | 0 | 0 |
| 87029 | TCC | 0 | 0.28266 | 0 | 0 | 0.03792 | 0 | 0 | 0 | 0 | 0 | 0.09652 | 0 | 0 | 0 | 0 | 0 | 0 | 0 | 0 | 0 | 0 | 0 | 0 | 0 | 0 | 0 | 0 | 0 | 0 | 0 | 0 |
| 70303 | TCG | 0 | 0 | 0.21621 | 0 | 0 | 0 | 0 | 0 | 0 | 0 | 0 | 0 | 0 | 0 | 0 | 0 | 0 | 0 | 0 | 0 | 0 | 0 | 0 | 0 | 0 | 0 | 0 | 0 | 0.12375 | 0 | 0 |
| 49899 | TCT | 0 | 0 | 0 | 0.29259 | 0 | 0.12225 | 0 | 0 | 0 | 0 | 0 | 0.04609 | 0 | 0 | 0 | 0 | 0 | 0 | 0 | 0 | 0 | 0 | 0 | 0 | 0 | 0 | 0 | 0 | 0 | 0 | 0 |
| 60265 | ACA | 0.53099 | 0 | 0 | 0 | 0 | 0 | 0 | 0 | 0 | 0 | 0 | 0 | 0 | 0 | 0 | 0 | 0 | 0 | 0.51937 | 0 | 0 | 0.03982 | 0 | 0 | 0 | 0 | 0 | 0 | 0 | 0 | 0 |
| 96192 | ACC | 0 | 0.64766 | 0 | 0 | 0 | 0 | 0 | 0 | 0 | 0 | 0 | 0 | 0 | 0 | 0 | 0 | 0 | 0 | 0 | 0.22455 | 0 | 0 | 0 | 0 | 0 | 0 | 0 | 0 | 0 | 0 | 0.06445 |
| 77038 | ACG | 0 | 0 | 0.47379 | 0 | 0 | 0 | 0 | 0 | 0 | 0 | 0 | 0 | 0 | 0 | 0 | 0 | 0 | 0 | 0 | 0 | 0 | 0 | 0.05192 | 0 | 0 | 0 | 0 | 0 | 0 | 0.57764 | 0 |
| 54237 | ACT | 0 | 0 | 0 | 0.55866 | 0 | 0 | 0 | 0 | 0 | 0 | 0 | 0 | 0 | 0 | 0 | 0 | 0 | 0 | 0 | 0 | 0.12538 | 0 | 0 | 0 | 0 | 0 | 0 | 0 | 0 | 0 | 0 |
| 25561 | GTA | 1.30668 | 0 | 0 | 0 | 0 | 0 | 0 | 0 | 0.05477 | 0 | 0 | 0 | 0.33254 | 0 | 0 | 0 | 0 | 0 | 0.59466 | 0 | 0 | 0 | 0 | 0.14866 | 0 | 0 | 0 | 0.10563 | 0 | 0 | 0 |
| 124963 | GTC | 0 | 0.21606 | 0 | 0 | 0 | 0 | 0.06402 | 0 | 0 | 0 | 0.03761 | 0 | 0 | 0.17285 | 0 | 0 | 0 | 0 | 0 | 0.76183 | 0 | 0 | 0 | 0 | 0.09763 | 0 | 0 | 0 | 0 | 0 | 0 |
| 93445 | GTG | 0 | 0 | 0.57574 | 0 | 0 | 0 | 0 | 0 | 0 | 0.10594 | 0 | 0 | 0 | 0 | 0.19798 | 0 | 0 | 0 | 0 | 0 | 0 | 0 | 0 | 0 | 0 | 0.09417 | 0 | 0 | 0.04602 | 0.31569 | 0 |
| 47363 | GTT | 0 | 0 | 0 | 0.21536 | 0 | 0 | 0 | 0.0549 | 0 | 0 | 0 | 0.03167 | 0 | 0 | 0 | 0.10134 | 0 | 0 | 0 | 0 | 0.98178 | 0 | 0 | 0 | 0 | 0 | 0.14991 | 0 | 0 | 0 | 0 |
| 69288 | TGG | 0 | 0 | 0 | 0 | 0.01588 | 0.0101 | 0 | 0 | 0 | 0 | 0 | 0 | 0 | 0 | 0.06639 | 0 | 0 | 0 | 0 | 0 | 0 | 0 | 0 | 0 | 0 | 0 | 0 | 0 | 0.01876 | 0 | 0 |
| 89733 | TAC | 0 | 0 | 0 | 0 | 0.25632 | 0 | 0.11813 | 0 | 0 | 0 | 0.06575 | 0 | 0 | 0 | 0 | 0 | 0.26523 | 0 | 0 | 0 | 0 | 0 | 0 | 0 | 0 | 0 | 0 | 0 | 0 | 0 | 0.01003 |
| 37410 | TAT | 0 | 0 | 0 | 0 | 0 | 0.29137 | 0 | 0.21919 | 0 | 0 | 0 | 0.03208 | 0 | 0 | 0 | 0 | 0 | 0.47581 | 0 | 0 | 0 | 0 | 0 | 0 | 0 | 0 | 0 | 0 | 0 | 0 | 0 |
| 1254 | TAA | 0 | 0 | 0 | 0 | 0 | 0 | 0 | 0 | 0.23923 | 0 | 0 | 0 | 0 | 0 | 0 | 0 | 0 | 0 | 0 | 0 | 0 | 0 | 0 | 0 | 0 | 0 | 0 | 0 | 0 | 0 | 0 |
| 2851 | TAG | 0 | 0 | 0 | 0 | 0 | 0 | 0 | 0 | 0 | 0.21045 | 0 | 0 | 0 | 0 | 0 | 0 | 0 | 0 | 0 | 0 | 0 | 0 | 0.03508 | 0 | 0 | 0 | 0 | 0 | 0.03508 | 0 | 0 |
| 5507 | TGA | 0 | 0 | 0 | 0 | 0.10895 | 0.01816 | 0 | 0 | 0 | 0 | 0 | 0 | 0.19975 | 0 | 0 | 0 | 0 | 0 | 0 | 0 | 0 | 0 | 0 | 0 | 0 | 0 | 0 | 0.03632 | 0 | 0 | 0 |

|  |  | **Var.** |  |  |  |  |  |  |  |  |  |  |  |  |  |  |  |  |  |  |  |  |  |  |  |  |  |  |  |  |  |  |  |  |
| --- | --- | --- | --- | --- | --- | --- | --- | --- | --- | --- | --- | --- | --- | --- | --- | --- | --- | --- | --- | --- | --- | --- | --- | --- | --- | --- | --- | --- | --- | --- | --- | --- | --- | --- |
| Ref. |  | **AAT** | **CCA** | **CCC** | **CCG** | **CCT** | **CAA** | **CAG** | **AGA** | **AGG** | **CGA** | **CGC** | **CGG** | **CGT** | **AGC** | **AGT** | **TCA** | **TCC** | **TCG** | **TCT** | **ACA** | **ACC** | **ACG** | **ACT** | **GTA** | **GTC** | **GTG** | **GTT** | **TGG** | **TAC** | **TAT** | **TAA** | **TAG** | TGA |
| 91754 | GCA | 0 | 0.08283 | 0 | 0 | 0 | 0 | 0 | 0 | 0 | 0 | 0 | 0 | 0 | 0 | 0 | 0.1188 | 0 | 0 | 0 | 0.35748 | 0 | 0 | 0 | 0.53404 | 0 | 0 | 0 | 0 | 0 | 0 | 0 | 0 | 0 |
| 129116 | GCC | 0 | 0 | 0.05267 | 0 | 0 | 0 | 0 | 0 | 0 | 0 | 0 | 0 | 0 | 0 | 0 | 0 | 0.17349 | 0 | 0 | 0 | 0.47322 | 0 | 0 | 0 | 0.22306 | 0 | 0 | 0 | 0 | 0 | 0 | 0 | 0 |
| 114409 | GCG | 0 | 0 | 0 | 0.02884 | 0 | 0 | 0 | 0 | 0 | 0 | 0 | 0 | 0 | 0 | 0 | 0 | 0 | 0.10576 | 0 | 0 | 0 | 0.32865 | 0 | 0 | 0 | 0.53143 | 0 | 0 | 0 | 0 | 0 | 0 | 0 |
| 95119 | GCT | 0 | 0 | 0 | 0 | 0.07675 | 0 | 0 | 0 | 0 | 0 | 0 | 0 | 0 | 0 | 0 | 0 | 0 | 0 | 0.11459 | 0 | 0 | 0 | 0.38688 | 0 | 0 | 0 | 0.11144 | 0 | 0 | 0 | 0 | 0 | 0 |
| 39785 | TGC | 0 | 0 | 0 | 0 | 0 | 0 | 0 | 0 | 0 | 0 | 0.19605 | 0 | 0 | 0.07792 | 0 | 0 | 0.07792 | 0 | 0 | 0 | 0 | 0 | 0 | 0 | 0 | 0 | 0 | 0.05027 | 0.51527 | 0 | 0 | 0 | 0.03268 |
| 20431 | TGT | 0 | 0 | 0 | 0 | 0 | 0 | 0 | 0 | 0 | 0 | 0 | 0 | 0.21046 | 0 | 0.06363 | 0 | 0 | 0 | 0.21536 | 0 | 0 | 0 | 0 | 0 | 0 | 0 | 0 | 0.0881 | 0 | 0.47477 | 0 | 0 | 0.03426 |
| 159365 | GAC | 0 | 0 | 0 | 0 | 0 | 0 | 0 | 0 | 0 | 0 | 0 | 0 | 0 | 0 | 0 | 0 | 0 | 0 | 0 | 0 | 0 | 0 | 0 | 0 | 0.05083 | 0 | 0 | 0 | 0.04392 | 0 | 0 | 0 | 0 |
| 116027 | GAT | 0.12669 | 0 | 0 | 0 | 0 | 0 | 0 | 0 | 0 | 0 | 0 | 0 | 0 | 0 | 0 | 0 | 0 | 0 | 0 | 0 | 0 | 0 | 0 | 0 | 0 | 0 | 0.01465 | 0 | 0 | 0.03534 | 0 | 0 | 0 |
| 109354 | GAA | 0 | 0 | 0 | 0 | 0 | 0.13991 | 0 | 0 | 0 | 0 | 0 | 0 | 0 | 0 | 0 | 0 | 0 | 0 | 0 | 0 | 0 | 0 | 0 | 0.01097 | 0 | 0 | 0 | 0 | 0 | 0 | 0.02103 | 0 | 0 |
| 188595 | GAG | 0 | 0 | 0 | 0 | 0 | 0 | 0.09703 | 0 | 0 | 0 | 0 | 0 | 0 | 0 | 0 | 0 | 0 | 0 | 0 | 0 | 0 | 0 | 0 | 0 | 0 | 0.06151 | 0 | 0 | 0 | 0 | 0 | 0.01326 | 0 |
| 127303 | TTC | 0 | 0 | 0 | 0 | 0 | 0 | 0 | 0 | 0 | 0 | 0 | 0 | 0 | 0 | 0 | 0 | 0.06677 | 0 | 0 | 0 | 0 | 0 | 0 | 0 | 0.04399 | 0 | 0 | 0 | 0.06677 | 0 | 0 | 0 | 0 |
| 45082 | TTT | 0 | 0 | 0 | 0 | 0 | 0 | 0 | 0 | 0 | 0 | 0 | 0 | 0 | 0 | 0 | 0 | 0 | 0 | 0.06655 | 0 | 0 | 0 | 0 | 0 | 0 | 0 | 0.04436 | 0 | 0 | 0.0488 | 0 | 0 | 0 |
| 87998 | GGA | 0 | 0 | 0 | 0 | 0 | 0 | 0 | 0.08296 | 0 | 0.03523 | 0 | 0 | 0 | 0 | 0 | 0 | 0 | 0 | 0 | 0 | 0 | 0 | 0 | 0.02046 | 0 | 0 | 0 | 0 | 0 | 0 | 0 | 0 | 0.00909 |
| 130985 | GGC | 0 | 0 | 0 | 0 | 0 | 0 | 0 | 0 | 0 | 0 | 0.02061 | 0 | 0 | 0.13513 | 0 | 0 | 0 | 0 | 0 | 0 | 0 | 0 | 0 | 0 | 0.02061 | 0 | 0 | 0 | 0 | 0 | 0 | 0 | 0 |
| 41918 | GGG | 0 | 0 | 0 | 0 | 0 | 0 | 0 | 0 | 0.07634 | 0 | 0 | 0.02624 | 0 | 0 | 0 | 0 | 0 | 0 | 0 | 0 | 0 | 0 | 0 | 0 | 0 | 0.05487 | 0 | 0.00477 | 0 | 0 | 0 | 0 | 0 |
| 71843 | GGT | 0 | 0 | 0 | 0 | 0 | 0 | 0 | 0 | 0 | 0 | 0 | 0 | 0.02784 | 0 | 0.17817 | 0 | 0 | 0 | 0 | 0 | 0 | 0 | 0 | 0 | 0 | 0 | 0.01114 | 0 | 0 | 0 | 0 | 0 | 0 |
| 70062 | CAC | 0 | 0 | 0.19411 | 0 | 0 | 0.04567 | 0.13274 | 0 | 0 | 0 | 0.43105 | 0 | 0 | 0 | 0 | 0 | 0 | 0 | 0 | 0 | 0 | 0 | 0 | 0 | 0 | 0 | 0 | 0 | 0.36396 | 0 | 0 | 0 | 0 |
| 45883 | CAT | 0.085 | 0 | 0 | 0 | 0.04577 | 0.06102 | 0.15256 | 0 | 0 | 0 | 0 | 0 | 0.45333 | 0 | 0 | 0 | 0 | 0 | 0 | 0 | 0 | 0 | 0 | 0 | 0 | 0 | 0 | 0 | 0 | 0.46204 | 0 | 0 | 0 |
| 20898 | ATA | 0 | 0 | 0 | 0 | 0 | 0 | 0 | 0.16269 | 0 | 0 | 0 | 0 | 0 | 0 | 0 | 0 | 0 | 0 | 0 | 1.13886 | 0 | 0 | 0 | 0.63642 | 0 | 0 | 0 | 0 | 0 | 0 | 0 | 0 | 0 |
| 139627 | ATC | 0 | 0 | 0 | 0 | 0 | 0 | 0 | 0 | 0 | 0 | 0 | 0 | 0 | 0.06947 | 0 | 0 | 0 | 0 | 0 | 0 | 0.15828 | 0 | 0 | 0 | 0.70832 | 0 | 0 | 0 | 0 | 0 | 0 | 0 | 0 |
| 60895 | ATT | 0.02135 | 0 | 0 | 0 | 0 | 0 | 0 | 0 | 0 | 0 | 0 | 0 | 0 | 0 | 0.02792 | 0 | 0 | 0 | 0 | 0 | 0 | 0 | 0.11495 | 0 | 0 | 0 | 0.80138 | 0 | 0 | 0 | 0 | 0 | 0 |
| 70629 | AAA | 0.04389 | 0 | 0 | 0 | 0 | 0.06088 | 0 | 0.15433 | 0 | 0 | 0 | 0 | 0 | 0 | 0 | 0 | 0 | 0 | 0 | 0.04389 | 0 | 0 | 0 | 0 | 0 | 0 | 0 | 0 | 0 | 0 | 0 | 0 | 0 |
| 161919 | AAG | 0.09387 | 0 | 0 | 0 | 0 | 0 | 0.04138 | 0 | 0.06979 | 0 | 0 | 0 | 0 | 0 | 0 | 0 | 0 | 0 | 0 | 0 | 0 | 0.02903 | 0 | 0 | 0 | 0 | 0 | 0 | 0 | 0 | 0 | 0.00432 | 0 |
| 26878 | CTA | 0 | 0.253 | 0 | 0 | 0 | 0.04465 | 0 | 0 | 0 | 0.11906 | 0 | 0 | 0 | 0 | 0 | 0 | 0 | 0 | 0 | 0 | 0 | 0 | 0 | 0.11534 | 0 | 0 | 0 | 0 | 0 | 0 | 0 | 0 | 0 |
| 142305 | CTC | 0 | 0 | 0.04708 | 0 | 0 | 0 | 0 | 0 | 0 | 0 | 0.07379 | 0 | 0 | 0 | 0 | 0 | 0 | 0 | 0 | 0 | 0 | 0 | 0 | 0 | 0.1033 | 0 | 0 | 0 | 0 | 0 | 0 | 0 | 0 |
| 101325 | CTG | 0 | 0 | 0 | 0.06218 | 0 | 0 | 0.07402 | 0 | 0 | 0 | 0 | 0.04145 | 0 | 0 | 0 | 0 | 0 | 0 | 0 | 0 | 0 | 0 | 0 | 0 | 0 | 0.1283 | 0 | 0 | 0 | 0 | 0 | 0 | 0 |
| 55859 | CTT | 0 | 0 | 0 | 0 | 0.0555 | 0 | 0 | 0 | 0 | 0 | 0 | 0 | 0.01969 | 0 | 0 | 0 | 0 | 0 | 0 | 0 | 0 | 0 | 0 | 0 | 0 | 0 | 0.14859 | 0 | 0 | 0 | 0 | 0 | 0 |
| 11387 | TTA | 0 | 0 | 0 | 0 | 0 | 0 | 0 | 0 | 0 | 0 | 0 | 0 | 0 | 0 | 0 | 0.47422 | 0 | 0 | 0 | 0 | 0 | 0 | 0 | 0.11417 | 0 | 0 | 0 | 0 | 0 | 0 | 0.00878 | 0 | 0.01756 |
| 78788 | TTG | 0 | 0 | 0 | 0 | 0 | 0 | 0 | 0 | 0 | 0 | 0 | 0 | 0 | 0 | 0 | 0 | 0 | 0.11423 | 0 | 0 | 0 | 0 | 0 | 0 | 0 | 0.09519 | 0 | 0.01777 | 0 | 0 | 0 | 0.00381 | 0 |
| 106678 | ATG | 0 | 0 | 0 | 0 | 0 | 0 | 0 | 0 | 0.03281 | 0 | 0 | 0 | 0 | 0 | 0 | 0 | 0 | 0 | 0 | 0 | 0 | 0.42745 | 0 | 0 | 0 | 0.30747 | 0 | 0 | 0 | 0 | 0 | 0 | 0 |
| 107930 | AAC | 2.9899 | 0 | 0 | 0 | 0 | 0 | 0 | 0 | 0 | 0 | 0 | 0 | 0 | 0.15195 | 0 | 0 | 0 | 0 | 0 | 0 | 0.1223 | 0 | 0 | 0 | 0 | 0 | 0 | 0 | 0.01668 | 0 | 0 | 0 | 0 |
| 61171 | AAT | 0 | 0 | 0 | 0 | 0 | 0 | 0 | 0 | 0 | 0 | 0 | 0 | 0 | 0 | 0.188 | 0 | 0 | 0 | 0 | 0 | 0 | 0 | 0.05395 | 0 | 0 | 0 | 0 | 0 | 0 | 0.02289 | 0 | 0 | 0 |
| 72641 | CCA | 0 | 0 | 0.31663 | 2.07321 | 0.60985 | 0.01927 | 0 | 0 | 0 | 0.03166 | 0 | 0 | 0 | 0 | 0 | 0.1473 | 0 | 0 | 0 | 0.03717 | 0 | 0 | 0 | 0 | 0 | 0 | 0 | 0 | 0 | 0 | 0 | 0 | 0 |
| 63677 | CCC | 0 | 0.27325 | 0 | 0.23713 | 0.70198 | 0 | 0 | 0 | 0 | 0 | 0.03141 | 0 | 0 | 0 | 0 | 0 | 0.21986 | 0 | 0 | 0 | 0.03298 | 0 | 0 | 0 | 0 | 0 | 0 | 0 | 0 | 0 | 0 | 0 | 0 |
| 78843 | CCG | 0 | 2.00398 | 0.22577 | 0 | 0.51368 | 0 | 0.04566 | 0 | 0 | 0 | 0 | 0.0241 | 0 | 0 | 0 | 0 | 0 | 0.14966 | 0 | 0 | 0 | 0.03932 | 0 | 0 | 0 | 0 | 0 | 0 | 0 | 0 | 0 | 0 | 0 |
| 60141 | CCT | 0 | 0.79314 | 0.6967 | 0.68173 | 0 | 0 | 0 | 0 | 0 | 0 | 0 | 0 | 0.00998 | 0 | 0 | 0 | 0 | 0 | 0.21616 | 0 | 0 | 0 | 0.06318 | 0 | 0 | 0 | 0 | 0 | 0 | 0 | 0 | 0 | 0 |
| 84681 | CAA | 0 | 0.02834 | 0 | 0 | 0 | 0 | 0.84907 | 0 | 0 | 0.1795 | 0 | 0 | 0 | 0 | 0 | 0 | 0 | 0 | 0 | 0 | 0 | 0 | 0 | 0 | 0 | 0 | 0 | 0 | 0 | 0 | 0.15588 | 0 | 0 |
| 106039 | CAG | 0 | 0 | 0 | 0.05375 | 0 | 0.70069 | 0 | 0 | 0 | 0 | 0 | 0.11034 | 0 | 0 | 0 | 0 | 0 | 0 | 0 | 0 | 0 | 0 | 0 | 0 | 0 | 0 | 0 | 0 | 0 | 0 | 0 | 0.24991 | 0 |
| 37686 | AGA | 0 | 0 | 0 | 0 | 0 | 0 | 0 | 0 | 0.73767 | 0.4909 | 0 | 0 | 0 | 0.16982 | 0.10083 | 0 | 0 | 0 | 0 | 0.16186 | 0 | 0 | 0 | 0 | 0 | 0 | 0 | 0 | 0 | 0 | 0 | 0 | 0.04246 |
| 39861 | AGG | 0 | 0 | 0 | 0 | 0 | 0 | 0 | 0.99847 | 0 | 0 | 0 | 0.71498 | 0 | 0.07275 | 0.06021 | 0 | 0 | 0 | 0 | 0 | 0 | 0.07777 | 0 | 0 | 0 | 0 | 0 | 0.07024 | 0 | 0 | 0 | 0 | 0 |
| 58510 | CGA | 0 | 0.04615 | 0 | 0 | 0 | 0.15211 | 0 | 0.40335 | 0 | 0 | 0.71612 | 0.64263 | 0.43411 | 0 | 0 | 0 | 0 | 0 | 0 | 0 | 0 | 0 | 0 | 0 | 0 | 0 | 0 | 0 | 0 | 0 | 0 | 0 | 0.08716 |
| 80514 | CGC | 0 | 0 | 0.03229 | 0 | 0 | 0 | 0 | 0 | 0 | 0.43471 | 0 | 0.18258 | 1.6494 | 0.05092 | 0 | 0 | 0 | 0 | 0 | 0 | 0 | 0 | 0 | 0 | 0 | 0 | 0 | 0 | 0 | 0 | 0 | 0 | 0 |
| 38584 | CGG | 0 | 0 | 0 | 0.04924 | 0 | 0 | 0.26436 | 0 | 0.71791 | 1.2881 | 0.39395 | 0 | 0.26177 | 0 | 0 | 0 | 0 | 0 | 0 | 0 | 0 | 0 | 0 | 0 | 0 | 0 | 0 | 0.19697 | 0 | 0 | 0 | 0 | 0 |
| 38270 | CGT | 0 | 0 | 0 | 0 | 0.05487 | 0 | 0 | 0 | 0 | 0.62712 | 3.33682 | 0.25869 | 0 | 0 | 0.07839 | 0 | 0 | 0 | 0 | 0 | 0 | 0 | 0 | 0 | 0 | 0 | 0 | 0 | 0 | 0 | 0 | 0 | 0 |
| 79886 | AGC | 0 | 0 | 0 | 0 | 0 | 0 | 0 | 0.04632 | 0.07636 | 0 | 0.09263 | 0 | 0 | 0 | 1.90772 | 0 | 0 | 0 | 0 | 0 | 0.10515 | 0 | 0 | 0 | 0 | 0 | 0 | 0 | 0 | 0 | 0 | 0 | 0 |
| 43894 | AGT | 0.20732 | 0 | 0 | 0 | 0 | 0 | 0 | 0.07746 | 0.20504 | 0 | 0 | 0 | 0.10252 | 3.09154 | 0 | 0 | 0 | 0 | 0 | 0 | 0 | 0 | 0.33718 | 0 | 0 | 0 | 0 | 0 | 0 | 0 | 0 | 0 | 0 |
| 44792 | TCA | 0 | 0.25228 | 0 | 0 | 0 | 0 | 0 | 0 | 0 | 0 | 0 | 0 | 0 | 0 | 0 | 0 | 0.54251 | 2.32408 | 0.45321 | 0.18977 | 0 | 0 | 0 | 0 | 0 | 0 | 0 | 0 | 0 | 0 | 0.0134 | 0 | 0.02902 |
| 87029 | TCC | 0 | 0 | 0.2413 | 0 | 0 | 0 | 0 | 0 | 0 | 0 | 0 | 0 | 0 | 0 | 0 | 0.28152 | 0 | 0.35965 | 0.8434 | 0 | 0.24704 | 0 | 0 | 0 | 0 | 0 | 0 | 0 | 0.02873 | 0 | 0 | 0 | 0 |
| 70303 | TCG | 0 | 0 | 0 | 0.18634 | 0 | 0 | 0 | 0 | 0 | 0 | 0 | 0 | 0 | 0 | 0 | 1.46366 | 0.41108 | 0 | 0.4125 | 0 | 0 | 0.13228 | 0 | 0 | 0 | 0 | 0 | 0.02134 | 0 | 0 | 0 | 0.02703 | 0 |
| 49899 | TCT | 0 | 0 | 0 | 0 | 0.17836 | 0 | 0 | 0 | 0 | 0 | 0 | 0 | 0 | 0 | 0 | 0.41684 | 1.15032 | 0.57115 | 0 | 0 | 0 | 0 | 0.16834 | 0 | 0 | 0 | 0 | 0 | 0 | 0.10221 | 0 | 0 | 0 |
| 60265 | ACA | 0 | 0.12113 | 0 | 0 | 0 | 0 | 0 | 0.11615 | 0 | 0 | 0 | 0 | 0 | 0 | 0 | 0.11283 | 0 | 0 | 0 | 0 | 0.42977 | 1.83523 | 0.53431 | 0 | 0 | 0 | 0 | 0 | 0 | 0 | 0 | 0 | 0 |
| 96192 | ACC | 0 | 0 | 0.68821 | 0 | 0 | 0 | 0 | 0 | 0 | 0 | 0 | 0 | 0 | 0.08109 | 0 | 0 | 0.18505 | 0 | 0 | 0.34202 | 0 | 0.35034 | 0.82647 | 0 | 0 | 0 | 0 | 0 | 0 | 0 | 0 | 0 | 0 |
| 77038 | ACG | 0 | 0 | 0 | 0.09216 | 0 | 0 | 0 | 0 | 0.02337 | 0 | 0 | 0 | 0 | 0 | 0 | 0 | 0 | 0.12332 | 0 | 1.6083 | 0.40759 | 0 | 0.55168 | 0 | 0 | 0 | 0 | 0 | 0 | 0 | 0 | 0 | 0 |
| 54237 | ACT | 0.09034 | 0 | 0 | 0 | 0.19544 | 0 | 0 | 0 | 0 | 0 | 0 | 0 | 0 | 0 | 0.29869 | 0 | 0 | 0 | 0.1475 | 0.62319 | 1.19107 | 0.68219 | 0 | 0 | 0 | 0 | 0 | 0 | 0 | 0 | 0 | 0 | 0 |
| 25561 | GTA | 0 | 0 | 0 | 0 | 0 | 0 | 0 | 0 | 0 | 0 | 0 | 0 | 0 | 0 | 0 | 0 | 0 | 0 | 0 | 0 | 0 | 0 | 0 | 0 | 1.46317 | 3.71269 | 0.28168 | 0 | 0 | 0 | 0 | 0 | 0 |
| 124963 | GTC | 0 | 0 | 0 | 0 | 0 | 0 | 0 | 0 | 0 | 0 | 0 | 0 | 0 | 0 | 0 | 0 | 0 | 0 | 0 | 0 | 0 | 0 | 0 | 0.3465 | 0 | 0.79303 | 0.72902 | 0 | 0 | 0 | 0 | 0 | 0 |
| 93445 | GTG | 0 | 0 | 0 | 0 | 0 | 0 | 0 | 0 | 0 | 0 | 0 | 0 | 0 | 0 | 0 | 0 | 0 | 0 | 0 | 0 | 0 | 0 | 0 | 1.11616 | 0.96634 | 0 | 0.20654 | 0 | 0 | 0 | 0 | 0 | 0 |
| 47363 | GTT | 0 | 0 | 0 | 0 | 0 | 0 | 0 | 0 | 0 | 0 | 0 | 0 | 0 | 0 | 0 | 0 | 0 | 0 | 0 | 0 | 0 | 0 | 0 | 0.15624 | 1.86221 | 0.46661 | 0 | 0 | 0 | 0 | 0 | 0 | 0 |
| 69288 | TGG | 0 | 0 | 0 | 0 | 0 | 0 | 0 | 0 | 0.03608 | 0 | 0 | 0.11546 | 0 | 0 | 0 | 0 | 0 | 0.02309 | 0 | 0 | 0 | 0 | 0 | 0 | 0 | 0 | 0 | 0 | 0 | 0 | 0 | 0.09092 | 0.04041 |
| 89733 | TAC | 0 | 0 | 0 | 0 | 0 | 0 | 0 | 0 | 0 | 0 | 0 | 0 | 0 | 0 | 0 | 0 | 0.36107 | 0 | 0 | 0 | 0 | 0 | 0 | 0 | 0 | 0 | 0 | 0 | 0 | 2.04941 | 0.02452 | 0.02786 | 0 |
| 37410 | TAT | 0.02138 | 0 | 0 | 0 | 0 | 0 | 0 | 0 | 0 | 0 | 0 | 0 | 0 | 0 | 0 | 0 | 0 | 0 | 0.14702 | 0 | 0 | 0 | 0 | 0 | 0 | 0 | 0 | 0 | 3.98824 | 0 | 0.03742 | 0.0401 | 0 |
| 1254 | TAA | 0 | 0 | 0 | 0 | 0 | 1.83413 | 0 | 0 | 0 | 0 | 0 | 0 | 0 | 0 | 0 | 0 | 0 | 0 | 0 | 0 | 0 | 0 | 0 | 0 | 0 | 0 | 0 | 0 | 0.31898 | 0.07974 | 0 | 0.79745 | 1.5949 |
| 2851 | TAG | 0 | 0 | 0 | 0 | 0 | 0 | 0.98211 | 0 | 0 | 0 | 0 | 0 | 0 | 0 | 0 | 0 | 0 | 0.07015 | 0 | 0 | 0 | 0 | 0 | 0 | 0 | 0 | 0 | 0.2806 | 0.24553 | 0.1403 | 0.1403 | 0 | 0 |
| 5507 | TGA | 0 | 0 | 0 | 0 | 0 | 0 | 0 | 0.09079 | 0 | 0.56292 | 0 | 0 | 0 | 0 | 0 | 0.18159 | 0 | 0 | 0 | 0 | 0 | 0 | 0 | 0 | 0 | 0 | 0 | 0.10895 | 0 | 0 | 0.43581 | 0 | 0 |
