# Supplementary material for: Codon optimization underpins generalist parasitism in fungi
Source: eLife. 2017 Feb 3;6:e22472. doi: 10.7554/eLife.22472 (PMC5315462; doi:10.7554/eLife.22472)
Supplement: Figure 5—source data 1. — DOI: http://dx.doi.org/10.7554/eLife.22472.018 [file elife-22472-fig5-data1.docx]

**Figure 5 – source data 1.** Summary of gene expression data used for the analysis of tAI in host-induced genes

| **Species** | **Code name** | **Transcriptome data source** | **Host range** | **Design** | **no. Induced** |
| --- | --- | --- | --- | --- | --- |
| *Aspergillus fumigatus* | Aspfu | Morton CO, Varga JJ, Hornbach A, Mezger M, Sennefelder H, Kneitz S, Kurzai O, Krappmann S, Einsele H, Nierman WC, Rogers TR, Loeffler J. The temporal dynamics of differential gene expression in Aspergillus fumigatus interacting with human immature dendritic cells in vitro. PLoS One. 2011 Jan 14;6(1):e16016. (Table S1) | Gen | Genes significantly induced by *A. fumigatus* (log2 fold >= 0.86) during a 12 h co-incubation with human immature dendritic cells at either 3h, 6h, 9h or 12h. Genes were identified by SAM (multi-class) analysis of whole genome transcription. | 130 |
| *Beauveria bassiana* | Beaba | Xiao G, Ying SH, Zheng P, Wang ZL, Zhang S, Xie XQ, Shang Y, St Leger RJ, Zhao GP, Wang C, Feng MG. Genomic perspectives on the evolution of fungal entomopathogenicity in Beauveria bassiana. Sci Rep. 2012;2:483. (Table S20) | Gen | Genes belonging to the 100 most expressed in Locust hind wings (LW) or in Cotton bollworm blood (CB) but not in Corn root exudates (RE) | 166 |
| *Blumeria graminis* | Blugr | Hacquard S, Kracher B, Maekawa T, Vernaldi S, Schulze-Lefert P, Ver Loren van Themaat E. Mosaic genome structure of the barley powdery mildew pathogen and conservation of transcriptional programs in divergent hosts. Proc Natl Acad Sci U S A. 2013 Jun 11;110(24):E2219-28. (Table S6) | Spe | Gene Blumeria graminis f. sp. hordei isolates A6 and K1 induced (log2>=1) during infection on Arabidopsis at either 12, 18 or 24 hours | 567 |
| *Botrytis cinerea* | Botci_v2 | Amselem J, Cuomo CA, van Kan JA, Viaud M, Benito EP, Couloux A, Coutinho PM, de Vries RP, Dyer PS, Fillinger S, Fournier E, Gout L, Hahn M, Kohn L, Lapalu N, Plummer KM, Pradier JM, Quévillon E, Sharon A, Simon A, ten Have A, Tudzynski B, Tudzynski P, Wincker P, Andrew M, Anthouard V, Beever RE, Beffa R, Benoit I, Bouzid O, Brault B, Chen Z, Choquer M, Collémare J, Cotton P, Danchin EG, Da Silva C, Gautier A, Giraud C, Giraud T, Gonzalez C, Grossetete S, Güldener U, Henrissat B, Howlett BJ, Kodira C, Kretschmer M, Lappartient A, Leroch M, Levis C, Mauceli E, Neuvéglise C, Oeser B, Pearson M, Poulain J, Poussereau N, Quesneville H, Rascle C, Schumacher J, Ségurens B, Sexton A, Silva E, Sirven C, Soanes DM, Talbot NJ, Templeton M, Yandava C, Yarden O, Zeng Q, Rollins JA, Lebrun MH, Dickman M. Genomic analysis of the necrotrophic fungal pathogens Sclerotinia sclerotiorum and Botrytis cinerea. PLoS Genet. 2011 Aug;7(8):e1002230. | Gen | Genes induced in sunflower cotyledons at 2dpi (vs in vitro grown mycelium) with p-value<0.1, fold induction >=1.5 | 220 |
| *Colletotrichum higginsianum* | Colhi | O'Connell RJ, Thon MR, Hacquard S, Amyotte SG, Kleemann J, Torres MF, Damm U, Buiate EA, Epstein L, Alkan N, Altmüller J, Alvarado-Balderrama L, Bauser CA, Becker C, Birren BW, Chen Z, Choi J, Crouch JA, Duvick JP, Farman MA, Gan P, Heiman D, Henrissat B, Howard RJ, Kabbage M, Koch C, Kracher B, Kubo Y, Law AD, Lebrun MH, Lee YH, Miyara I, Moore N, Neumann U, Nordström K, Panaccione DG, Panstruga R, Place M, Proctor RH, Prusky D, Rech G, Reinhardt R, Rollins JA, Rounsley S, Schardl CL, Schwartz DC, Shenoy N, Shirasu K, Sikhakolli UR, Stüber K, Sukno SA, Sweigard JA, Takano Y, Takahara H, Trail F, van der Does HC, Voll LM, Will I, Young S, Zeng Q, Zhang J, Zhou S, Dickman MB, Schulze-Lefert P, Ver Loren van Themaat E, Ma LJ, Vaillancourt LJ. Lifestyle transitions in plant pathogenic Colletotrichum fungi deciphered by genome and transcriptome analyses. Nat Genet. 2012 Sep;44(9):1060-5. (Supplementary Table 13) | Spe | Genes significantly upregulated (log2 fold >=3 and p-value<=0.01) in either in planta appressoria (PA), biotrophic phase (BP) or necrotrophic phase (NP) relative to in vitro appressoria (VA). using the mean number of reads measured for three biological replicates are presented. The number of reads for each gene in each sample was normalized relative to the total number of mapped reads in each sample. | 711 |
| *Cryptococcus neoformans* | Cryne | Chen Y, Toffaletti DL, Tenor JL, Litvintseva AP, Fang C, Mitchell TG, McDonald TR, Nielsen K, Boulware DR, Bicanic T, Perfect JR. The Cryptococcus neoformans transcriptome at the site of human meningitis. MBio. 2014 Feb 4;5(1):e01087-13. (file mbo001141726so2.xlsx) | Gen | Genes significantly upregulated (log2 fold >=1.9, p-value<=0.01) in vivo compared to ex vivo. Orthologs in JEC21 isolate genome were identified through a reciprocal BlastP search. | 161 |
| *Dothistroma septosporum* | Dotse | Bradshaw RE, Guo Y, Sim AD, Kabir MS, Chettri P, Ozturk IK, Hunziker L, Ganley RJ, Cox MP. Genome-wide gene expression dynamics of the fungal pathogen Dothistroma septosporum throughout its infection cycle of the gymnosperm host Pinus radiata. Mol Plant Pathol. 2016 Feb;17(2):210-24. (Table S6, S7 and S8) | Spe | Genes induced >=3 fold early in planta, mid in planta or late in planta compared to in vitro | 409 |
| *Fusarium graminearum* | Fusgr | Zhang XW, Jia LJ, Zhang Y, Jiang G, Li X, Zhang D, Tang WH. In planta stage-specific fungal gene profiling elucidates the molecular strategies of Fusarium graminearum growing inside wheat coleoptiles. Plant Cell. 2012 Dec;24(12):5159-76. (Supplemental Data set 1) | Gen | Genes significantly induced (>=2.6 fold) in planta at 16 hpi, 40 hpi or 64 hpi compared to in vitro grown conidia. | 309 |
| *Melampsora larici-populina* | Mellp | Duplessis S, Hacquard S, Delaruelle C, Tisserant E, Frey P, Martin F, Kohler A. Melampsora larici-populina transcript profiling during germination and timecourse infection of poplar leaves reveals dynamic expression patterns associated with virulence and biotrophy. Mol Plant Microbe Interact. 2011 Jul;24(7):808-18. (Supplementary Table 2) | Spe | Genes specifically expressed in at least one time-point during the time-course infection (24, 48, 96, 168 hours post-inoculation; in planta) and not detected in urediniospores (resting, USp; germinating, UspG) using custom oligoarrays, and showing at least 500 reads in planta. | 972 |
| *Metarhizium acridum* | Metac | Gao Q, Jin K, Ying SH, Zhang Y, Xiao G, Shang Y, Duan Z, Hu X, Xie XQ, Zhou G, Peng G, Luo Z, Huang W, Wang B, Fang W, Wang S, Zhong Y, Ma LJ, St Leger RJ, Zhao GP, Pei Y, Feng MG, Xia Y, Wang C. Genome sequencing and comparative transcriptomics of the model entomopathogenic fungi Metarhizium anisopliae and M. acridum. PLoS Genet. 2011 Jan 6;7(1):e1001264. (Table S19) | Gen | Genes belonging to the 100 most highly expressed genes dunring infection of locust or cockroach cuticles | 143 |
| *Moniliophthora roreri* | Monro | Bailey BA, Melnick RL, Strem MD, Crozier J, Shao J, Sicher R, Phillips-Mora W, Ali SS, Zhang D, Meinhardt L. Differential gene expression by Moniliophthora roreri while overcoming cacao tolerance in the field. Mol Plant Pathol. 2014 Sep;15(7):711-29. (mpp12134-sup-0002-as2.xlsx 'Diff expressed' sheet) | Spe | Genes induced at least 2 fold in the susceptible cacao clones Pound-7 or CATIE-1000 compared to the tolerant clones CATIE-R7 and CATIE-R4. | 477 |
| *Ophiocordyceps unilateralis* | Ophun | de Bekker C, Ohm RA, Loreto RG, Sebastian A, Albert I, Merrow M, Brachmann A, Hughes DP. Gene expression during zombie ant biting behavior reflects the complexity underlying fungal parasitic behavioral manipulation. BMC Genomics. 2015 Aug 19;16:620. (Additional file 7 Sheet 2) | Spe | up-regulated Ophiocordyceps unilateralis s.l. genes (2-fold and significant Q<0.05, minimum expression of 500) in live manipulated Camponotus castaneus ant heads (Manipulated Biting Behavior samples) compared to gene expression in culture (Fungal Baseline Expression samples) and significantly downregulated (2-fold) After manipulated bitting behavior. | 128 |
| *Puccinia graminis* | Pucgr | Duplessis S, Cuomo CA, Lin YC, Aerts A, Tisserant E, Veneault-Fourrey C, Joly DL, Hacquard S, Amselem J, Cantarel BL, Chiu R, Coutinho PM, Feau N, Field M, Frey P, Gelhaye E, Goldberg J, Grabherr MG, Kodira CD, Kohler A, Kües U, Lindquist EA, Lucas SM, Mago R, Mauceli E, Morin E, Murat C, Pangilinan JL, Park R, Pearson M, Quesneville H, Rouhier N, Sakthikumar S, Salamov AA, Schmutz J, Selles B, Shapiro H, Tanguay P, Tuskan GA, Henrissat B, Van de Peer Y, Rouzé P, Ellis JG, Dodds PN, Schein JE, Zhong S, Hamelin RC, Grigoriev IV, Szabo LJ, Martin F. Obligate biotrophy features unraveled by the genomic analysis of rust fungi. Proc Natl Acad Sci U S A. 2011 May 31;108(22):9166-71. (Table S13, S14, S15) | Spe | Non-redundant list of genes belonging to the Top 100 most highly expressed Puccinia graminis f. sp. tritici genes in infected wheat, or Top 100 most highly up-regulated Puccinia graminis f. sp. tritici genes in infected wheat compared to resting urediniospores (Usp) or Top 100 most highly up-regulated Puccinia graminis f. sp. tritici genes in germinating urediniospores (USpG) compared to resting urediniospores (USp) and induced at least 2-fold in Wheat. | 144 |
| *Sclerotinia sclerotiorum* | Sclsc_v2 | This work | Gen | Genes significantly upregulated (>=4 fold) on all three different host plants | 352 |
| *Zymoseptoria tritici* | Mycgr | Kellner R, Bhattacharyya A, Poppe S, Hsu TY, Brem RB, Stukenbrock EH. Expression profiling of the wheat pathogen Zymoseptoria tritici reveals genomic patterns of transcription and host-specific regulatory programs. Genome Biol Evol. 2014 May 14;6(6):1353-65. (Table S3) | Spe | Significantly differentially expressed genes (false discovery rate < 0.01) comparing in planta and axenic growth | 128 |
