# Supplementary material for: Codon optimization underpins generalist parasitism in fungi
Source: eLife. 2017 Feb 3;6:e22472. doi: 10.7554/eLife.22472 (PMC5315462; doi:10.7554/eLife.22472)
Supplement: Figure 5—source data 2. — DOI: http://dx.doi.org/10.7554/eLife.22472.019 [file elife-22472-fig5-data2.docx]

**Figure 5 – source data 2.** Distribution of host-induced genes according to tAI (as % of all host-induced genes)

| Generalist species | |  |  |  |  |  |  |  |  |
| --- | --- | --- | --- | --- | --- | --- | --- | --- | --- |
| tAI percentiles | | *Aspergillus fumigatus* | *Beauveria bassiana* | *Botrytis cinerea* | *Cryptococcus neoformans* | *Fusarium graminearum* | *Metarhizium acridum* | *Sclerotinia sclerotiorum* |  |
| 0 | 0.1 | 3.85 | 6.63 | 8.64 | 4.35 | 4.85 | 0.00 | 8.24 |  |
| 0.1 | 0.2 | 2.31 | 3.01 | 10.91 | 2.48 | 3.56 | 1.40 | 9.38 |  |
| 0.2 | 0.3 | 7.69 | 6.02 | 11.82 | 3.11 | 5.18 | 0.70 | 9.09 |  |
| 0.3 | 0.4 | 8.46 | 4.82 | 7.73 | 3.11 | 7.44 | 1.40 | 11.36 |  |
| 0.4 | 0.5 | 6.92 | 6.02 | 11.82 | 4.35 | 11.00 | 1.40 | 11.93 |  |
| 0.5 | 0.6 | 10.00 | 3.61 | 10.45 | 3.73 | 9.39 | 2.10 | 8.24 |  |
| 0.6 | 0.7 | 13.08 | 3.01 | 12.27 | 8.07 | 13.92 | 3.50 | 7.39 |  |
| 0.7 | 0.8 | 15.38 | 5.42 | 13.18 | 11.18 | 14.56 | 6.99 | 12.22 |  |
| 0.8 | 0.9 | 12.31 | 13.25 | 6.82 | 16.77 | 12.94 | 12.59 | 9.09 |  |
| 0.9 | 1 | 20.00 | 48.19 | 6.36 | 42.86 | 17.15 | 69.93 | 13.07 |  |

| Specialist species | |  |  |  |  |  |  |  |  |
| --- | --- | --- | --- | --- | --- | --- | --- | --- | --- |
| tAI percentiles | | *Blumeria graminis* | *Colletotrichum higginsianum* | *Dothistroma septosporum* | *Melampsora larici-populina* | *Moniliophthora roreri* | *Zymoseptoria tritici* | *Ophiocordyceps unilateralis* | *Puccinia graminis* |
| 0 | 0.1 | 17.21 | 8.72 | 15.89 | 10.30 | 5.03 | 11.72 | 14.84 | 7.64 |
| 0.1 | 0.2 | 14.99 | 7.88 | 10.27 | 12.21 | 5.45 | 7.81 | 13.28 | 9.72 |
| 0.2 | 0.3 | 11.24 | 9.85 | 10.02 | 9.66 | 5.03 | 8.59 | 10.94 | 13.19 |
| 0.3 | 0.4 | 9.20 | 9.99 | 11.49 | 7.86 | 7.76 | 10.16 | 9.38 | 11.11 |
| 0.4 | 0.5 | 8.69 | 10.55 | 14.18 | 11.25 | 7.13 | 7.03 | 10.94 | 8.33 |
| 0.5 | 0.6 | 9.54 | 9.42 | 9.29 | 8.81 | 8.18 | 14.84 | 6.25 | 8.33 |
| 0.6 | 0.7 | 6.30 | 11.53 | 8.07 | 9.13 | 12.37 | 11.72 | 9.38 | 11.11 |
| 0.7 | 0.8 | 7.33 | 11.25 | 7.82 | 9.45 | 11.53 | 15.63 | 6.25 | 11.11 |
| 0.8 | 0.9 | 5.28 | 10.55 | 7.33 | 10.40 | 15.09 | 7.03 | 9.38 | 7.64 |
| 0.9 | 1 | 10.22 | 10.27 | 5.62 | 10.93 | 22.43 | 5.47 | 9.38 | 11.81 |
