# Supplementary material for: Codon optimization underpins generalist parasitism in fungi
Source: eLife. 2017 Feb 3;6:e22472. doi: 10.7554/eLife.22472 (PMC5315462; doi:10.7554/eLife.22472)
Supplement: Figure 5—source data 3. — DOI: http://dx.doi.org/10.7554/eLife.22472.020 [file elife-22472-fig5-data3.docx]

**Figure 5 – source data 3.** Distribution of secreted protein genes according to tAI (as % of all host-induced genes)

| **Generalist species** | | |  |  |  |  |  |  |  |  |  |  |  |  | |  |  |  |  |
| --- | --- | --- | --- | --- | --- | --- | --- | --- | --- | --- | --- | --- | --- | --- | --- | --- | --- | --- | --- |
| tAI percentiles | | *Alternaria brassicicola* | *Aspergillus fumigatus* | *Batrachochytrium dendrobatidis* | *Beauveria bassiana* | *Botrytis cinerea* | *Colletotrichum graminicola* | *Cryptococcus neoformans* | *Fusarium graminearum* | *Metarhizium acridum* | *Penicillium digitatum* | *Pyrenophora tritici-repentis* | *Rhizopus oryzae* | *Rhizoctonia solani* | *Sclerotinia sclerotiorum* | | *Verticilium dahliae* |  |  |
| 0 | 0.1 | 4.39 | 5.12 | 14.89 | 5.87 | 11.19 | 5.74 | 6.81 | 4.64 | 6.47 | 5.47 | 5.33 | 5.88 | 6.37 | 10.23 | | 4.88 |  |  |
| 0.1 | 0.2 | 5.53 | 6.58 | 12.98 | 7.13 | 9.17 | 5.97 | 7.74 | 5.42 | 7.15 | 6.50 | 5.07 | 8.62 | 5.53 | 8.23 | | 6.66 |  |  |
| 0.2 | 0.3 | 5.82 | 6.58 | 10.38 | 6.86 | 8.42 | 7.33 | 8.36 | 6.86 | 6.58 | 7.68 | 5.07 | 6.70 | 5.95 | 7.90 | | 5.88 |  |  |
| 0.3 | 0.4 | 6.39 | 6.21 | 8.20 | 9.75 | 9.28 | 7.78 | 7.12 | 7.52 | 7.38 | 7.68 | 6.01 | 8.89 | 9.08 | 9.68 | | 7.44 |  |  |
| 0.4 | 0.5 | 7.82 | 8.53 | 8.20 | 9.21 | 8.00 | 8.08 | 7.43 | 7.71 | 8.51 | 11.08 | 7.13 | 6.16 | 9.81 | 9.34 | | 10.88 |  |  |
| 0.5 | 0.6 | 7.44 | 7.19 | 9.15 | 9.66 | 7.46 | 10.35 | 10.22 | 9.54 | 8.51 | 11.52 | 9.45 | 9.44 | 9.08 | 9.79 | | 9.77 |  |  |
| 0.6 | 0.7 | 9.83 | 11.45 | 8.74 | 10.38 | 8.85 | 12.31 | 12.07 | 10.72 | 11.69 | 11.67 | 8.51 | 7.80 | 12.42 | 9.12 | | 11.10 |  |  |
| 0.7 | 0.8 | 12.98 | 12.42 | 6.83 | 12.64 | 10.23 | 13.29 | 12.69 | 12.68 | 12.94 | 9.90 | 11.94 | 10.94 | 12.94 | 9.45 | | 12.99 |  |  |
| 0.8 | 0.9 | 16.32 | 17.30 | 9.70 | 16.70 | 11.09 | 13.97 | 17.34 | 15.75 | 15.78 | 14.03 | 16.75 | 15.05 | 13.99 | 10.57 | | 15.43 |  |  |
| 0.9 | 1 | 23.47 | 18.64 | 10.93 | 11.82 | 16.31 | 15.18 | 10.22 | 19.15 | 14.98 | 14.48 | 24.74 | 20.52 | 14.82 | 15.68 | | 14.98 |  |  |

| **Specialist species** | | |  |  |  |  |  |  |  |  |  |  |  | |  | |  |
| --- | --- | --- | --- | --- | --- | --- | --- | --- | --- | --- | --- | --- | --- | --- | --- | --- | --- |
| tAI percentiles | | *Blumeria graminis* | *Passalora fulva* | *Dothistroma septosporum* | *Erysiphe necator* | *Moniliophthora roreri* | Pseudocercospora fijiensis | Zymoseptoria tritici | *Nosema ceranae* | *Ophiocordyceps unilateralis* | *Puccinia graminis* | *Puccinia triticina* | *Rozella allomycis* | *Wolfiporia cocos* | |  |  |
| 0 | 0.1 | 17.95 | 12.50 | 10.33 | 7.88 | 4.88 | 7.98 | 9.46 | 11.52 | 15.77 | 7.59 | 9.81 | 15.43 | 6.74 | |  |  |
| 0.1 | 0.2 | 10.54 | 9.00 | 8.57 | 5.76 | 5.35 | 4.75 | 8.29 | 9.42 | 11.04 | 9.79 | 9.20 | 10.00 | 8.53 | |  |  |
| 0.2 | 0.3 | 11.40 | 8.65 | 7.86 | 7.58 | 5.89 | 8.59 | 6.31 | 10.99 | 12.27 | 10.36 | 10.01 | 8.57 | 5.69 | |  |  |
| 0.3 | 0.4 | 7.83 | 7.60 | 8.57 | 6.97 | 5.89 | 8.44 | 7.48 | 12.04 | 9.46 | 11.08 | 9.33 | 9.14 | 7.49 | |  |  |
| 0.4 | 0.5 | 9.26 | 8.57 | 9.04 | 8.18 | 7.07 | 8.44 | 7.48 | 9.95 | 9.35 | 10.08 | 9.74 | 9.14 | 12.87 | |  |  |
| 0.5 | 0.6 | 9.40 | 9.44 | 7.63 | 10.30 | 8.20 | 8.44 | 8.64 | 8.90 | 10.81 | 10.60 | 10.15 | 8.57 | 11.38 | |  |  |
| 0.6 | 0.7 | 6.41 | 11.89 | 9.74 | 9.39 | 9.81 | 9.82 | 10.28 | 10.47 | 7.55 | 11.32 | 11.38 | 11.14 | 12.43 | |  |  |
| 0.7 | 0.8 | 7.83 | 10.31 | 11.50 | 9.39 | 11.83 | 12.12 | 12.38 | 11.52 | 7.32 | 10.55 | 9.88 | 8.86 | 12.13 | |  |  |
| 0.8 | 0.9 | 7.41 | 13.46 | 12.09 | 13.94 | 16.59 | 14.11 | 11.33 | 12.04 | 7.09 | 9.74 | 9.06 | 10.57 | 12.43 | |  |  |
| 0.9 | 1 | 11.97 | 8.57 | 14.67 | 20.61 | 24.49 | 17.33 | 18.34 | 3.14 | 9.35 | 8.88 | 11.44 | 8.57 | 10.33 | |  |  |

| **Non parasitic species** | | |  |  |  |  |  |  |  |  |
| --- | --- | --- | --- | --- | --- | --- | --- | --- | --- | --- |
| tAI percentiles | | *Agaricus bisporus* | *Chaetomium globosum* | *Gonapodya prolifera* | *Laccaria bicolor* | *Myceliophthora thermophila* | *Oidiodendron maius* | *Rhodotorula toruloides* | *Serpula lacrymans* | *Tuber melanosporum* |
| 0 | 0.1 | 5.91 | 12.33 | 12.91 | 12.27 | 5.39 | 15.09 | 10.48 | 6.08 | 6.92 |
| 0.1 | 0.2 | 8.05 | 8.88 | 9.17 | 9.49 | 6.22 | 11.95 | 7.26 | 5.80 | 6.57 |
| 0.2 | 0.3 | 7.25 | 8.07 | 9.65 | 13.05 | 6.92 | 11.81 | 10.28 | 4.83 | 8.65 |
| 0.3 | 0.4 | 8.05 | 9.43 | 10.37 | 11.41 | 6.36 | 11.04 | 8.06 | 7.46 | 9.52 |
| 0.4 | 0.5 | 9.13 | 9.25 | 9.05 | 11.84 | 8.44 | 12.02 | 10.08 | 7.18 | 11.07 |
| 0.5 | 0.6 | 10.07 | 9.43 | 8.81 | 11.70 | 7.47 | 10.34 | 10.28 | 8.98 | 7.96 |
| 0.6 | 0.7 | 9.80 | 9.07 | 8.81 | 9.34 | 12.72 | 8.74 | 8.87 | 11.74 | 11.25 |
| 0.7 | 0.8 | 10.07 | 11.33 | 11.58 | 8.35 | 11.76 | 6.99 | 10.89 | 11.33 | 9.69 |
| 0.8 | 0.9 | 11.54 | 11.51 | 10.98 | 6.56 | 16.74 | 6.64 | 14.72 | 14.64 | 12.98 |
| 0.9 | 1 | 20.13 | 10.70 | 8.69 | 5.99 | 17.98 | 5.38 | 9.07 | 21.96 | 15.40 |
