# Supplementary material for: Codon optimization underpins generalist parasitism in fungi
Source: eLife. 2017 Feb 3;6:e22472. doi: 10.7554/eLife.22472 (PMC5315462; doi:10.7554/eLife.22472)
Supplement: Figure 5—source data 4. — Cat. Category; NP, non parasitic; Gen, generalist; Spe, specialist; No. Sec. Prot., Number of secreted proteins. DOI: http://dx.doi.org/10.7554/eLife.22472.021 [file elife-22472-fig5-data4.docx]

**Figure 5 – source data 4.** Codon optimization values in secreted and non-secreted proteins for each of the 45 fungal genomes analyzed in this work. Cat. Category; NP, non parasitic; Gen, generalist; Spe, specialist; No. Sec. Prot., Number of secreted proteins.

| Species | Cat. | No. Sec. prot. | S secreted | S non  secreted |
| --- | --- | --- | --- | --- |
| *Agaricus bisporus* | NP | 745 | 0.582 | 0.326 |
| *Alternaria brassicicola* | Gen | 1049 | 0.572 | 0.353 |
| *Aspergillus fumigatus* | Gen | 785 | 0.714 | 0.593 |
| *Batrachochytrium dendrobatidis* | Gen | 720 | 0.545 | 0.545 |
| *Beauveria bassiana* | Gen | 1078 | 0.546 | 0.622 |
| *Blumeria graminis* | Spe | 704 | 0.249 | 0.086 |
| *Botrytis cinerea* | Gen | 899 | 0.709 | 0.58 |
| *Chaetomium globosum* | NP | 1106 | 0.179 | 0.145 |
| *Passalora fulva* | Spe | 1145 | 0.028 | -0.02 |
| *Colletotrichum graminicola* | Gen | 1288 | 0.624 | 0.559 |
| *Colletotrichum higginsianum* |  | 1860 | 0.344 | 0.341 |
| *Cryptococcus neoformans* | Gen | 313 | 0.876 | 0.842 |
| *Dothistroma septosporum* | Spe | 820 | 0.404 | 0.187 |
| *Encephalitozoon intestinalis* |  | 84 | 0.31 | 0.311 |
| *Erysiphe necator* | Spe | 332 | 0.254 | 0.165 |
| *Fusarium graminearum* | Gen | 1534 | 0.852 | 0.771 |
| *Pseudogymnoascus destructans* | | 455 | 0.586 | 0.475 |
| *Gonapodya prolifera* | NP | 829 | 0.059 | 0.12 |
| *Laccaria bicolor* | NP | 1403 | 0.055 | -0.03 |
| *Magnaporthe oryzae* |  | 1576 | 0.404 | 0.431 |
| *Melampsora larici-populina* |  | 1483 | 0.273 | 0.182 |
| *Metarhizium acridum* | Gen | 861 | 0.613 | 0.648 |
| *Moniliophthora roreri* | Spe | 1685 | 0.668 | 0.353 |
| *Pseudocercospora fijiensis* | Spe | 633 | 0.09 | 0.234 |
| *Zymoseptoria tritici* | Spe | 828 | 0.073 | -0.04 |
| *Myceliophthora thermophila* | NP | 723 | 0.284 | 0.241 |
| *Nosema ceranae* | Spe | 182 | 0.034 | 0.042 |
| *Oidiodendron maius* | NP | 1433 | 0.165 | 0.157 |
| *Ophiocordyceps unilateralis* | Spe | 854 | 0.276 | 0.147 |
| *Penicillium digitatum* | Gen | 677 | 0.721 | 0.529 |
| *Puccinia graminis* | Spe | 2095 | -0.09 | -0.03 |
| *Puccinia triticina* | Spe | 1393 | 0.178 | 0.209 |
| *Pyrenophora tritici-repentis* | Gen | 1133 | 0.587 | 0.377 |
| *Rhizopus oryzae* | Gen | 731 | 0.697 | 0.62 |
| *Rhizoctonia solani* | Gen | 917 | 0.632 | 0.388 |
| *Rhodotorula toruloides* | NP | 496 | 0.49 | 0.32 |
| *Rozella allomycis* | Spe | 350 | 0.224 | 0.237 |
| *Sclerotinia sclerotiorum* | Gen | 851 | 0.615 | 0.514 |
| *Serpula lacrymans* | NP | 725 | -0.08 | 0.002 |
| *Sporisorium reilianum* |  | 788 | 0.48 | 0.476 |
| *Stagonospora nodorum* |  | 1390 | 0.305 | 0.129 |
| *Taphrina deformans* |  | 264 | 0.323 | 0.418 |
| *Tuber melanosporum* | NP | 578 | 0.54 | 0.203 |
| *Verticilium dahliae* | Gen | 878 | 0.616 | 0.519 |
| *Wolfiporia cocos* | Spe | 668 | 0.367 | 0.208 |
